# Pseudouridine-modified tRNA fragments repress aberrant protein synthesis and predict leukaemic progression in myelodysplastic syndrome

**DOI:** 10.1038/s41556-022-00852-9

**Published:** 2022-03-15

**Authors:** Nicola Guzzi, Sowndarya Muthukumar, Maciej Cieśla, Gabriele Todisco, Phuong Cao Thi Ngoc, Magdalena Madej, Roberto Munita, Serena Fazio, Simon Ekström, Teresa Mortera-Blanco, Monika Jansson, Yasuhito Nannya, Mario Cazzola, Seishi Ogawa, Luca Malcovati, Eva Hellström-Lindberg, Marios Dimitriou, Cristian Bellodi

**Affiliations:** 1 Division of Molecular Hematology, Department of Laboratory Medicine, Lund Stem Cell Center, Faculty of Medicine, Lund University, Lund, Sweden; 2 Center for Hematology and Regenerative Medicine, Department of Medicine, Karolinska Institute, Stockholm, Sweden; 3 Department of Molecular Medicine, University of Pavia, Pavia, Italy; 4 BioMS−Swedish National Infrastructure for Biological Mass Spectrometry, Lund University, Lund, Sweden; 5 Department of Pathology and Tumor Biology, Kyoto University, Kyoto, Japan; 6 Institute for the Advanced Study of Human Biology, Kyoto University, Kyoto, Japan; 7 Department of Hematology Oncology, Fondazione IRCCS Policlinico San Matteo, Pavia, Italy

**Keywords:** RNA, Translation, Cancer stem cells, Haematopoietic stem cells, Small RNAs

## Abstract

Transfer RNA-derived fragments (tRFs) are emerging small noncoding RNAs that, although commonly altered in cancer, have poorly defined roles in tumorigenesis^1^. Here we show that pseudouridylation (Ψ) of a stem cell-enriched tRF subtype^2^, mini tRFs containing a 5′ terminal oligoguanine (mTOG), selectively inhibits aberrant protein synthesis programmes, thereby promoting engraftment and differentiation of haematopoietic stem and progenitor cells (HSPCs) in patients with myelodysplastic syndrome (MDS). Building on evidence that mTOG-Ψ targets polyadenylate-binding protein cytoplasmic 1 (PABPC1), we employed isotope exchange proteomics to reveal critical interactions between mTOG and functional RNA-recognition motif (RRM) domains of PABPC1. Mechanistically, this hinders the recruitment of translational co-activator PABPC1-interacting protein 1 (PAIP1)^3^ and strongly represses the translation of transcripts sharing pyrimidine-enriched sequences (PES) at the 5′ untranslated region (UTR), including 5′ terminal oligopyrimidine tracts (TOP) that encode protein machinery components and are frequently altered in cancer^4^. Significantly, mTOG dysregulation leads to aberrantly increased translation of 5′ PES messenger RNA (mRNA) in malignant MDS-HSPCs and is clinically associated with leukaemic transformation and reduced patient survival. These findings define a critical role for tRFs and Ψ in difficult-to-treat subsets of MDS characterized by high risk of progression to acute myeloid leukaemia (AML).

## Abstract (web summary)

Bellodi, Dimitriou and colleagues report that pseudouridine-modified transfer-RNA fragments modulate the translation of transcripts sharing pyrimidine-enriched sequences at their 5′ untranslated regions and their dysregulation impacts myelodysplastic syndrome pathogenesis.

## Main

**Fig. 1:**
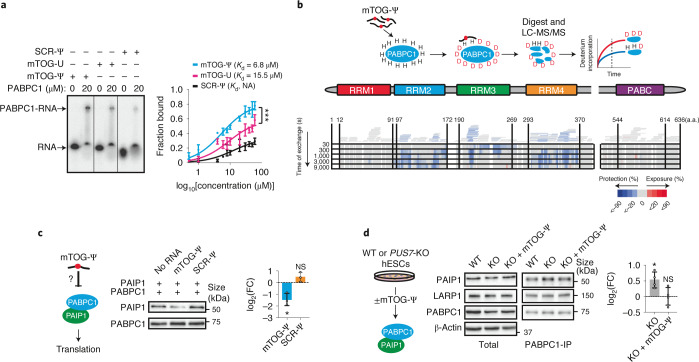
Molecular characterization of mTOG-Ψ PABPC1 binding reveals impairments in PAIP1 translation enhancer recruitment. **a**, Electrophoretic mobility shift assay showing direct interaction between mTOG-Ψ and PABPC1 (left). Unmodified mTOG (mTOG-U) and SCR-Ψ oligos are shown for comparison. Michaelis–Menten binding curve showing increased PABPC1 affinity for mTOG-Ψ (right). The fraction of RNA bound to PABPC1 is shown at increasing PABPC1 concentration. Data are the mean ± s.d. *K*_d_, dissociation constant; NA, not applicable. ***P = 0.0006, nonlinear regression; *n* = 3 biologically independent experiments. **b**, Schematic of the HDX-MS approach to delineate the molecular interactions between mTOG-Ψ and PABPC1 (top). The heat map shows difference in maximum deuterium (D) uptake ± mTOG-Ψ at different time points (bottom). Regions of PABPC1 protected or exposed from solvent exchange in the presence of mTOG-Ψ are shown in blue and red, respectively. Peptide coverage for PABPC1 is illustrated by bars above the heat map and is colour-coded based on the average deuterium uptake across all time points. Numbers correspond to the amino acid (a.a.) residues in PABPC1. LC-MS/MS, liquid chromatography with tandem mass spectrometry. **c**, The PAIP1–PABPC1 interaction is inhibited by mTOG-Ψ. Recombinant PABPC1 and PAIP1 were incubated in the presence or absence of mTOG-Ψ (left). The fraction of PAIP1 that co-precipitated with PABPC1 was determined by western blotting (middle) and quantified (right) as the log_2_-transformed FC of the PAIP1 fraction co-precipitated with PABPC1 (mean ± s.d.) in mTOG-Ψ or SCR-Ψ conditions normalized to control samples without RNA. *P = 0.0415; and NS, not significant; two-tailed Welch’s *t*-test; *n* = 3 biologically independent experiments. **d**, PAIP1 recruitment to PABPC1 in hESCs is impaired by mTOG-Ψ. Endogenous PABPC1 was immunoprecipitated in WT and PUS7-KO hESCs ± mTOG-Ψ (middle; schematic of the experiment on the left). LARP1 was used as a control. The level PAIP1 co-immunoprecipitation (mean ± s.d.), normalized to the WT, was determined (right). *P = 0.0286; and NS, not significant; two-tailed Mann–Whitney *U* test; *n* = 4 biologically independent experiments. Source data

**Extended Data Fig. 1:**
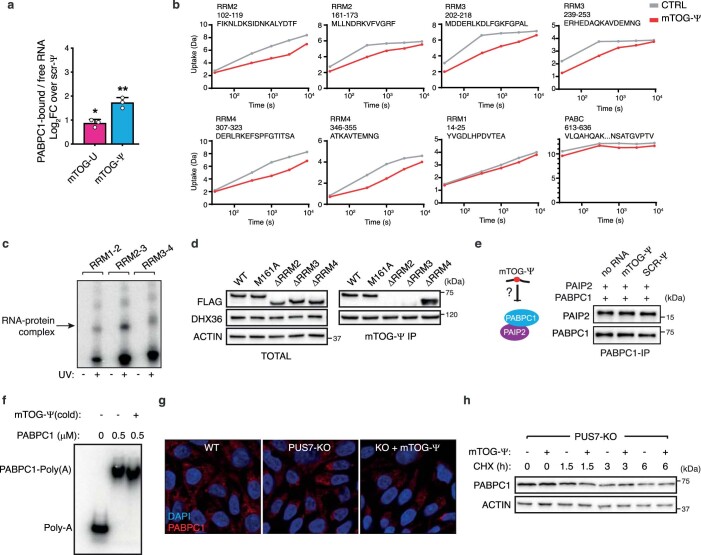
mTOG-Ψ bind to RRM2 and RRM3 within PABPC1. **a**, Graph shows fraction of PABPC1 bound to RNA normalized to unbound RNA as the log_2_ FC to SCR-Ψ oligo in EMSA experiments (*n*=3 independent experiments, mean ± SD). *p=0.0354; ***p=0.0002 (one-way ANOVA with multiple comparisons). **b**, Uptake plots show number of deuterium incorporated in selected peptides from indicated PABPC1 domains in HDX-MS time-course experiment in CTR or mTOG-Ψ conditions. Annotations indicate RRM, residue number and peptide sequence. Data points are mean of *n*=2 biological experiments. **c**, Binding assay shows significant and selective interaction between mTOG-Ψ and RRM2-3 upon UV-crosslinking. **d**, Immunoprecipitation experiments using biotinylated mTOG-Ψ oligos in cells transduced with FLAG-tagged full-length or different PABPC1 mutants. DHX36 is shown as a control mTOG-Ψ-bound protein^2^. **e**, mTOG-Ψ does not affect PABPC1-PAIP2 interaction. PAIP2 co-precipitated with PABPC1 is shown in the presence or absence of SCR and mTOG-Ψ oligos. **f**, mTOG-Ψ does not alter PABPC1 binding to poly(A). Representative EMSA shows radioactive-labelled poly(A) RNA incubated with recombinant PABPC1 in presence or absence of cold mTOG-Ψ. **g**, Immunofluorescence shows no changes in PABPC1 localization in *WT* and *PUS7-KO* hESC ± mTOG-Ψ. **h**, Protein stability analysis show no differences in PABPC1 levels ± mTOG-Ψ in hESCs following treatment with cycloheximide (CHX). Data in c-h represent *n*=3 independent experiments, except g, showing one representative experiment. Source data

A prominent Ψ ‘writer’ responsible for stress-inducible Ψ on different RNAs is the multi-substrate synthase PUS7 (refs. ^5–7^). PUS7-driven Ψ enables mTOG binding to PABPC1 and destabilization of the translation-initiation complex (eIF4F). This modulates translation and directly impacts embryonic and haematopoietic-stem-cell growth and fate commitment^2^. However, how this is achieved at the molecular level remains largely uncharacterized. We thus performed an electrophoretic mobility shift assay to determine the binding dynamics. This experiment unambiguously demonstrated that mTOG directly engaged PABPC1 in a Ψ-dependent manner, as revealed by a lower dissociation constant compared with unmodified mTOG or Ψ-modified scramble (SCR-Ψ) oligos (Fig. 1a and Extended Data Fig. 1a). Given that most of the RNA-binding capacity of PABPC1 lies within the modular amino (N)-terminal region consisting of four consecutive RRM domains^8^, we adapted hydrogen–deuterium exchange mass spectrometry (HDX-MS) to map the binding site of mTOG-Ψ to PABPC1 and reveal the molecular dynamics underlying their interaction^9^ (Fig. 1b). HDX-MS relies on the isotopic exchange of amide hydrogens on protein surfaces exposed to a deuterated solution. This enables the identification of protein–protein and protein–RNA interacting domains depending on the solvent accessibility and deuterium exchange rate in a given region. This unbiased approach uncovered a rapid binding between mTOG-Ψ and RRM3 of PABPC1, followed by decreased deuterium exchange in RRM2 and RRM4 with time, indicative of allosteric remodelling or additional mTOG-Ψ interactions in these regions (Fig. 1b, Extended Data Fig. 1b and Supplementary Table 1). Accordingly, in vitro binding assays validated the high and specific affinity of mTOG-Ψ for recombinant RRM2 and RRM3 (Extended Data Fig. 1c). Consistent with these and our previous results^2^, we performed pulldown experiments using biotinylated synthetic mTOG-Ψ oligos in cells transduced with full-length wild-type (WT) PABPC1, the PABPC1 deletion mutants ΔRRM2, ΔRRM3 and ΔRRM4 as well as a point mutant variant (PABPC1^M161A^) that abolishes eIF4G binding^8^, an essential component of the eIF4F complex. As expected, mTOG-Ψ efficiently associated with WT PABPC1, PABPC1^M161A^ and the ΔRRM4 mutant but failed to co-immunoprecipitate the ΔRRM2–3 mutants in vivo (Extended Data Fig. 1d).

Previous work illustrated that PABPC1 activity is modulated through critical interactions with PABP-interacting proteins 1 and 2—PAIP1 and PAIP2—which involve binding to regions within the RRM2 and RRM3 domains^10,11^. Specifically, PAIP1 stimulates translation through mutual binding of PABPC1 and eIF4A, resulting in mRNA circularization and closed-loop formation^3,10^. Conversely, PAIP2 reduces PABPC1 affinity for poly(A) tails and represses translation^11,12^. Motivated by our molecular findings, we investigated whether mTOG-Ψ could interfere with PAIP1 and PAIP2 recruitment to PABPC1. Strikingly, mTOG-Ψ strongly impaired recombinant PABPC1–PAIP1 binding without affecting the PABPC1–PAIP2 interaction, whereas no effects were observed following transfection with SCR-Ψ (Fig. 1c and Extended Data Fig. 1e). Significantly, PABPC1–PAIP1 binding was increased in *PUS7*-knockout (KO) human embryonic stem cells (hESCs) and was restored following mTOG-Ψ transduction (Fig. 1d). In contrast, mTOG-Ψ did not affect La-related protein 1 (LARP1) binding, a well-characterized PABPC1-interacting protein^13^ (Fig. 1d). Moreover, no effects on PABPC1 stability, localization and poly(A) tail affinity were noticeable following mTOG-Ψ treatment (Extended Data Fig. 1f–h).

**Fig. 2:**
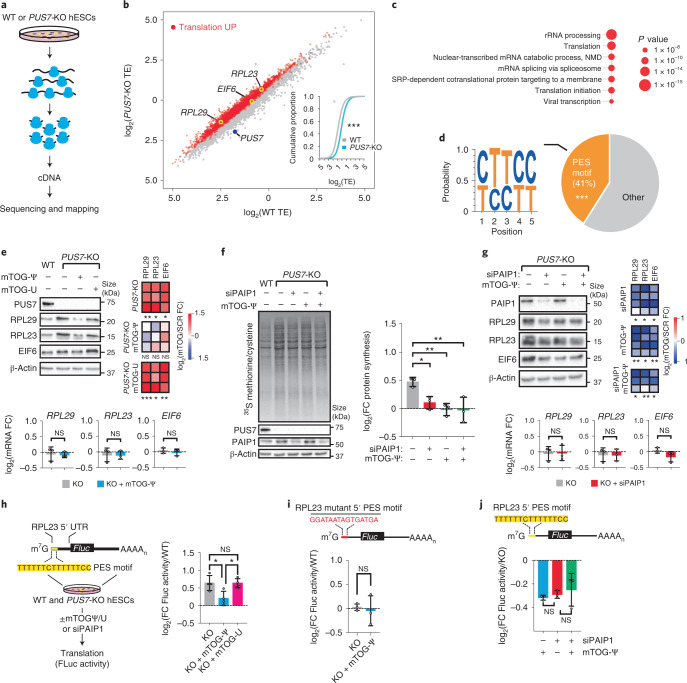
mTOG-Ψ controls the translation of select 5′ PES-containing mRNA subsets in a PAIP1–PABPC1-dependent manner. **a**,**b**, Transcriptome-wide analysis of TE in WT and *PUS7*-KO hESCs. **a**, Schematic of the ribosome profiling experiment. **b**, The 2,459 transcripts with log_2_(TE FC) > 1 and FDR < 0.2 are coloured (red). Inset: cumulative distribution of log_2_(TE) values for WT and *PUS7*-KO hESCs. ***P < 2.2 × 10^−16^; two-sided Wilcoxon signed-rank test. **c**, Gene ontology analysis of translationally upregulated mRNAs in *PUS7*-KO cells. **d**, Motif analysis (left) and pie chart (right) showing enrichment for 5′ PES scored within the first ten nucleotides from the transcription start site in translationally upregulated mRNAs. ***P < 0.001, hypergeometric test. **e**, Representative protein analysis of 5′ PES candidate genes from ribosome profiling in WT and *PUS7*-KO hESCs ± mTOG-Ψ or mTOG-U (top left). The heat maps show the FC in protein abundance (mTOG-Ψ or mTOG-U) relative to the WT (SCR-Ψ; top right); one-tailed Student’s *t*-test. *RPL29*, *RPL23* and *EIF6* mRNA levels in *PUS7-KO* hESCs ± mTOG-Ψ relative to the WT (bottom); one-way analysis of variance (ANOVA) with a multiple comparison test. **f**, Change in de novo protein synthesis rates in *PUS7*-KO hESCs ± siPAIP1 and mTOG-Ψ relative to the WT (right). *P = 0.0339, **P = 0.0059 (mTOG-Ψ) and **P = 0.0054 (siPAIP1 + mTOG-Ψ); one-way ANOVA with a multiple comparison test. **g**, Representative protein analysis of mTOG-regulated RPL29, RPL23 and EIF6 in WT and *PUS7*-KO hESCs following treatment with siPAIP1 ± mTOG-Ψ (top left). Heat map showing log_2_-transformed FC in protein levels normalized to *PUS7*-KO cells (top right); one-tailed paired Student’s *t*-test. *RPL29*, *RPL23* and *EIF6* mRNA levels in *PUS7*-KO hESCs ± siPAIP1 (bottom); one-way ANOVA with a multiple comparison test. **e**,**g**, *P *<* 0.05, **P < 0.01 and ***P < 0.001. **h**, Schematic depicting the translational reporter-based assay (left). Change in Firefly luciferase (Fluc) activity in *PUS7*-KO hESCs ± mTOG-Ψ or mTOG-U relative to the WT (right). *P = 0.0213 (KO versus KO + mTOG-Ψ) and *P = 0.0229 (KO + mTOG-Ψ versus KO + mTOG-U); one-way ANOVA with a multiple comparison test. **i**, Mutagenesis of the *RPL23* 5′ PES (top) hampers Fluc translation regulation in *PUS7*-KO ± mTOG-Ψ; two-tailed Student’s *t*-test. **j**, PAIP1 is required for mTOG-Ψ control of RPL23 5′ PES *cis*-regulatory activity. Change in Fluc activity in *PUS7*-KO hESCs treated with mTOG-Ψ or siPAIP1 relative to the *PUS7*-KO control. One-way ANOVA with a multiple comparison test. **e**–**j**, Data are the mean ± s.d. from *n* = 3 (**e**–**g**,**i**,**j**) or 4 (**h**) independent biological replicates; NS, not significant. **c**,**e**,**j**, Individual *P* values are provided. Source data

**Extended Data Fig. 2:**
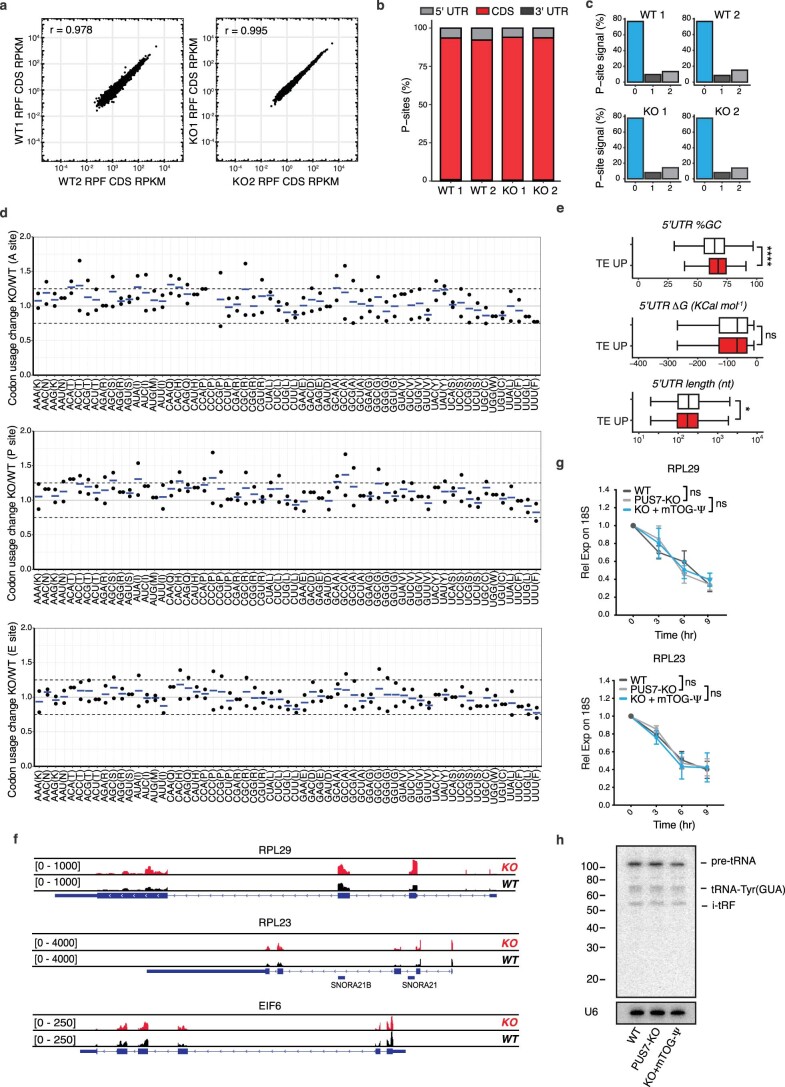
Ribosome profiling reveals translational regulation of 5’PES-containing mRNAs. **a**, Plots show reproducibility of ribosome profiling RPF libraries from *WT* and *PUS7-KO* hESCs. *r*, Pearson correlation coefficient. **b-c**, Percentage of P-sites in coding sequence (CDS) or untranslated regions (UTR) (b) and assigned to reading frames 0, 1 and 2 (c) are shown. **d**, Analysis of ribosome occupancy at individual codons for the A-, P- and E-site is shown as the ratio *PUS7-KO/WT*. Data are shown as values and mean of two independent experiments. **e**, 5’UTR analysis of translationally upregulated mRNAs in *PUS7-KO* hESCs shows increased GC percentage and decreased length compared to the transcriptome. Boxplots show the upper and lower whiskers (box boundaries), the upper and lower quartiles, and the median. ****p=1.63e−25; *p=0.01; ns, no statistical significance (two-sided Wilcoxon signed-rank test). TE UP *n*=2023 and, transcriptome *n*=16063. **f**, Genome browser tracks show representative reads from ribosome-protected fragments in *WT* and *PUS7-KO* for *RPL29*, *RPL23* and *EIF6* mRNAs. **g**, mTOG-Ψ do not affect RPL29 and RPL23 mRNA stability. Transcript levels were measured at indicated time points after Actinomycin-D treatment and normalized to the 18S rRNA. *n*=3 independent biological replicates at each time point, presented as mean±SD. **h**, tRNA-Tyr (GUA) northern blot analysis in hESCs *PUS7-KO* ± mTOG-Ψ U6 RNA is included as loading control, *n*=3 independent experiments. Source data

A wealth of studies have illustrated that eIF4F activity critically affects the translation of mRNA subsets characterized by complex 5′ UTRs or the presence of *cis*-regulatory sequences involved in development and tumorigenesis^4,14^. Intrigued by our findings that mTOG-Ψ impacts eIF4F assembly^2^, we investigated whether mTOG-driven regulation of translation initiation might steer distinct translational programmes in stem cells. To do this, we performed matched deep sequencing of total mRNA and ribosome-protected mRNA fragments isolated from WT or *PUS7*-KO hESCs harvested in the presence of the translation elongation inhibitor cycloheximide^15^ (Fig. 2a). The results from duplicate experiments were highly reproducible (Pearson’s coefficient, *r* > 0.98) and exhibited triplet periodicity with enrichment for coding sequences in ribosome-bound mRNAs without differences in the codon occupancy (Extended Data Fig. 2a–d). Comparative analysis of the translation efficiency (TE)—a metric for estimating the number of ribosomes associated with each transcript normalized to the total mRNA abundance—revealed a global TE increase in *PUS7*-KO hESCs, consistent with increased de novo protein synthesis rates in these cells^2^ (Fig. 2b). To further characterize the translatome regulated by PUS7 and mTOG in stem cells, we analysed mRNAs with a log_2_-transformed fold change (FC) in TE of more than one (>twofold) and false-detection rate (FDR) < 0.2 with no transcriptional differences (that is, |log_2_(FC mRNA KO/WT)| < 1) in *PUS7*-KO cells compared with the WT controls (Fig. 2b and Supplementary Table 2). Importantly, gene ontology analysis of 2,459 differentially translated mRNAs in the PUS7-depleted cells revealed a remarkable enrichment for terms related to translation and ribosome biogenesis (Fig. 2c). Building on these findings, we reasoned that a common feature within the regulatory regions of these mRNAs might account for the increased sensitivity to PUS7/mTOG translation regulation. Remarkably, 5′ UTR analysis of mRNAs differentially translated in *PUS7*-KO cells revealed higher GC content, shorter 5′ UTR lengths and a significant enrichment (41%) for mRNAs containing a 5′ PES near the transcription start site (Fig. 2d and Extended Data Fig. 2e). These included bona fide 5′ TOP and 5′ TOP-like mRNAs encoding components of the translation machinery such as ribosomal proteins and translation initiation factors that are involved in protein synthesis regulation and frequently altered in disease^16,17^ (Fig. 2e and Extended Data Fig. 2f).

**Extended Data Fig. 3:**
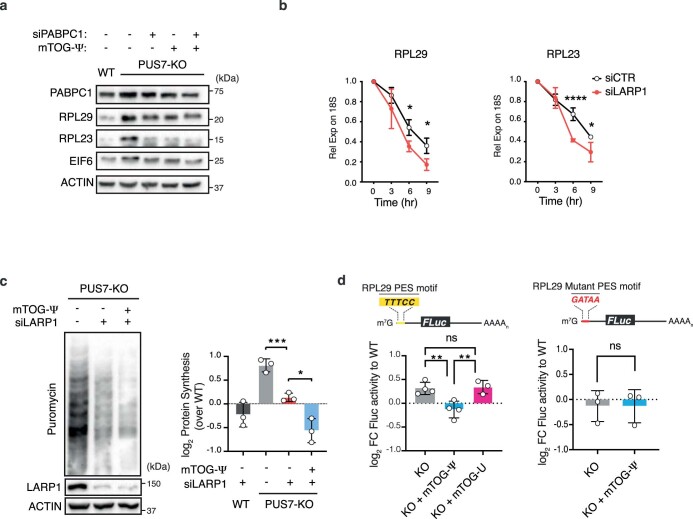
mTOG-Ψ-mediated translational control is PABPC1-dependent and LARP1-independent. **a**, Protein analysis of selected 5’PES mRNA candidates in *WT*, *PUS7-KO* hESCs. *n*=3 independent experiments. **b**, LARP1 stabilizes 5’PES-containing RPL29 and RPL23 transcripts. Corresponding mRNA levels measured at different time points following Actinomycin-D treatment and normalized to 18S rRNA are shown. For RPL23, *p=0.0353, and *p=0.0348; RPL29 ****p<0.0001, and *p=0.0177 (nonlinear regression analysis). *n*=3 independent biological replicates at each time point, presented as mean ± SD. **c**, mTOG-Ψ translation repression is LARP1-independent. Global protein synthesis measured by puromycin incorporation in *WT* and *PUS7-KO* hESC transfected with CTR or LARP1-targeting siRNA ± mTOG-Ψ. Graph shows log_2_ FC protein synthesis to WT, mean ± SD. *n*=3 independent biological replicates. ***p=0.001; *p=0.0112 (one-way ANOVA with multiple comparison). **d**, RPL29 5’UTR translational reporter with WT (left) and mutant (right) PES motif employed to assess the functional effect of mTOG-Ψ. Graph shows log_2_ FC FLuc activity to WT (mean ± SD) in *PUS7-KO* hESC ± mTOG-Ψ/U. *n*=3 independent biological replicates. **p=0.0030, **p=0.0030; ns, no statistical significance (one-way ANOVA with multiple comparison). Source data

Given that translation of 5′ PES-containing mRNAs is highly responsive to the assembly and function of the cap-binding translation initiation complex (eIF4F), we evaluated the direct contribution of mTOG-Ψ to the translation of selected transcripts identified through ribosome profiling in *PUS7*-KO cells. These included 5′ TOP and 5′ TOP-like candidates such as RPL29, RPL23 and EIF6 with roles in development and disease^18–22^, further examined as prototype 5′ PES mRNAs potentially regulated by mTOG-Ψ. Consistent with our translation analysis, the protein levels of RPL29, RPL23 and EIF6 were drastically increased in *PUS7*-KO hESCs and could be specifically restored only following transfection of mTOG-Ψ (Fig. 2e). These effects were independent of changes in mRNA transcription and stability, and were uncoupled from differences in the precursor and mature tRNA-Tyr(GUA), a well-established PUS7 substrate in eukaryotic cells^6^ (Fig. 2e and Extended Data Fig. 2g,h). As mTOG-Ψ modulates the PAIP1–PABPC1 axis, we theorized that a reduction in the cellular levels of PAIP1 might mimic mTOG-mediated translation repression in stem cells. We found that short interfering RNA (siRNA)-mediated PAIP1 depletion restored the de novo protein synthesis rates and completely phenocopied the mTOG effects in *PUS7*-KO cells (Fig. 2f). Next, we investigated whether translation of mTOG-responsive mRNAs was directly affected by downregulation of PABPC1 and PAIP1. To this end, we systematically examined the effects of siRNA-mediated PAIP1 and PABPC1 knockdown (KD) on the levels of RPL29, RPL23 and EIF6 in *PUS7*-KO hESCs in the absence or presence of mTOG-Ψ. Strikingly, depletion of either PABPC1 or PAIP1 selectively rescued increased translation of these mRNAs with no additional mTOG-Ψ-dependent translation repression observed (Fig. 2g and Extended Data Fig. 3a). This provides further support for our data showing that mTOG-Ψ critically modulates the PAIP1–PABPC1 interaction. Accordingly, we found that mTOG-Ψ-dependent repression of 5′ PES mRNA translation was independent of LARP1, which was previously shown to impact the stability and translation of this class of transcripts^23^. LARP1 depletion significantly affected the stability of mTOG-responsive transcripts and rescued translation in *PUS7*-KO cells (Extended Data Fig. 3b,c). However, we found that mTOG-Ψ treatment further repressed protein synthesis in LARP1-depleted *PUS7*-KO cells (Extended Data Fig. 3c). This indicates that mTOG and LARP1 regulate PABPC1 and translation through a distinct mechanism, consistent with findings that mTOG-Ψ does not affect LARP1 binding to PABPC1 (Fig. 1d). Next, we investigated whether translation control by mTOG-Ψ directly involves the 5′ PES motif embedded in the mRNAs identified through ribosome profiling (Fig. 2b). Using translation reporters that harboured RPL23 and RPL29 5′ UTRs, we demonstrated that the mTOG-Ψ repressive function was reliant on the presence of the 5′ PES sequences (Fig. 2h,i and Extended Data Fig. 3d). As expected, mTOG-Ψ regulation of 5′ PES *cis*-regulatory activity was PAIP1-dependent (Fig. 2j). These findings support recent evidence that the translation of 5′ PES-containing transcripts integrates multiple cell-type-specific control mechanisms^24,25^.

**Fig. 3:**
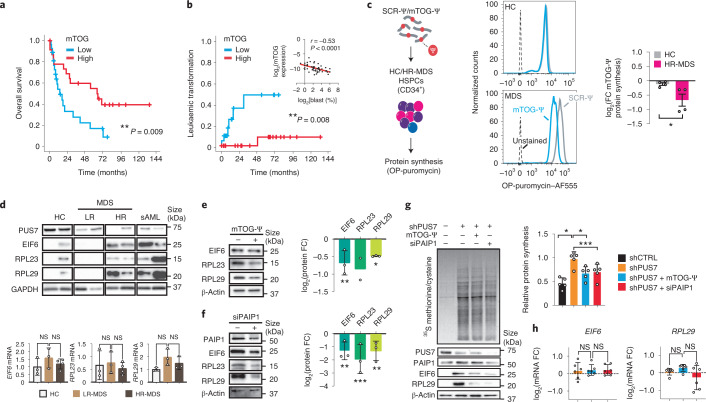
Dysregulation of mTOG-Ψ-driven translation control in HR-MDS-HSPCs predicts leukaemic progression. **a**,**b**, Patients with reduced HSPC mTOG levels have decreased overall survival (**a**; *n* = 50) and increased risk of AML progression (**b**; *n* = 43). Log-rank test. **b**, Inset: inverse correlation (Pearson’s *r*) between the relative HSPC mTOG expression levels and BM blast counts in patients with MDS (*n* = 50). **c**, mTOG-Ψ represses translation in MDS-HSPCs. Representative flow cytometric analysis (middle) of de novo protein synthesis in HSPCs from healthy controls (HC) and MDS-HSPCs following treatment with SCR-Ψ or mTOG-Ψ as illustrated in the schematic (left). The changes in protein levels, determined through OP-puromycin labelling, of mTOG-Ψ relative to SCR-Ψ (mean ± s.e.m.) in four independent HC individuals and patients with MDS are shown (right). *P = 0.0311, Welch’s two-tailed *t*-test. **d**, Protein analysis of patient-derived BMMCs demonstrated increased expression of mTOG-Ψ targets in HR-MDS and sAML compared with the HC and LR-MDS groups (top). No differences in the relative *EIF6*, *RPL23* and *RPL29* mRNA levels in patient-derived BMMCs relative to the HC group were observed (bottom). One-way ANOVA with a multiple comparison test; *n* = 3 or 4, each dot represents a patient. **e**,**f**, Delivery of synthetic mTOG-Ψ (**e**) and siPAIP1 (**f**) selectively rescues the translation of 5′ PES-containing *RPL29*, *RPL23* and *EIF6* mRNA in patient-derived HR-MDS cells (left). The changes in protein abundance in mononuclear cells from different patients with HR-MDS treated with mTOG-Ψ (**e**) and siPAIP1 (**f**) relative to the controls (SCR-Ψ and siCtrl, respectively) are shown (right). Two-way ANOVA with a multiple comparison test; *n* = 3 patient samples, except for RPL23 in **e**, where *n* = 2; each dot represents a patient. **e**, **P = 0.0015 and *P = 0.0195. **f**, **P = 0.0025 and ***P = 0.0005. **g**, The mTOG-Ψ–PAIP1 axis modulates translation in PUS7-depleted MDS-L cells. Representative de novo protein synthesis and analysis of mTOG-Ψ-regulated 5′ PES-containing mRNA in MDS-L cells infected with shRNA targeting *PUS7* (shPUS7) with or without mTOG-Ψ or siPAIP1 (20 nM) treatment (left). Relative protein synthesis in *PUS7*-KD MDS-L cells ± siPAIP1 or mTOG-Ψ (right). *P = 0.0215, *P = 0.0190 and ***P = 0.0001; one-way ANOVA with a multiple comparison test; *n* = 5 independent biological replicates. **h**, No changes in *EIF6* and *RPL29* transcription, relative to the control (shCTRL), were observed in the treatment groups in **g**. One-way ANOVA with a multiple comparison test; *n* = 6 independent biological replicates. **d**–**h**, Data are the mean ± s.d. NS, not significant. Source data

**Extended Data Fig. 4:**
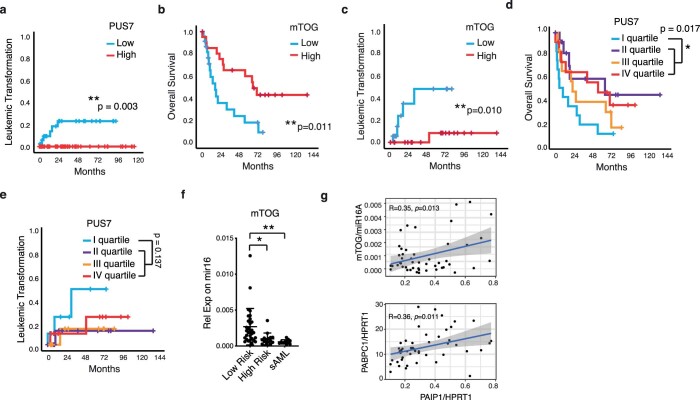
Clinical assessment of PUS7 and mTOG dysregulation in MDS. **a**, Increased risk of AML progression in MDS patients with low PUS7 expression (HR=102, *n*=100) evaluated in HSPC from a previously published study^29^. **b**, Decreased overall patient survival (*n*=44, HR=3.1, 95% CI, 1.4-6.9) and **c**, increased risk of AML progression (*n*=38, HR=10.1, 95% CI, 1.2-88) in patients with reduced HSPC mTOG expression. Patients with −7/del(7q) were excluded from the analysis. p-values were calculated using log-rank test. **d-e**, PUS7 levels in primary MDS-HSPC. Low PUS7 mRNA expression (I quartile) is associated with decreased overall survival (HR = 2.4, 95% CI 1.14–5.04, *n*=50). p-values were calculated using log-rank test. **f**, mTOG levels are reduced in high risk MDS and secondary AML (sAML) HSPC. mTOG levels were quantified by RT-qPCR and normalized to mir16 levels. *p =0.0205; **p=0.0082 (one-way ANOVA with multiple comparison). *n*=30 LR, 15 HR and 11 AML individual patient samples, presented as mean ± SD. **g**, Pairwise correlation between mTOG and PAIP1 or PABPC1 mRNA levels in primary MDS-HSPC (*n*=50). Expression of each of the RNAs was quantified by ddPCR and normalized to miR16 (mTOG) or HPRT1 levels (PAIP1 and PABPC1). The shaded region in each graph represents the confidence interval. Source data

**Extended Data Fig. 5:**
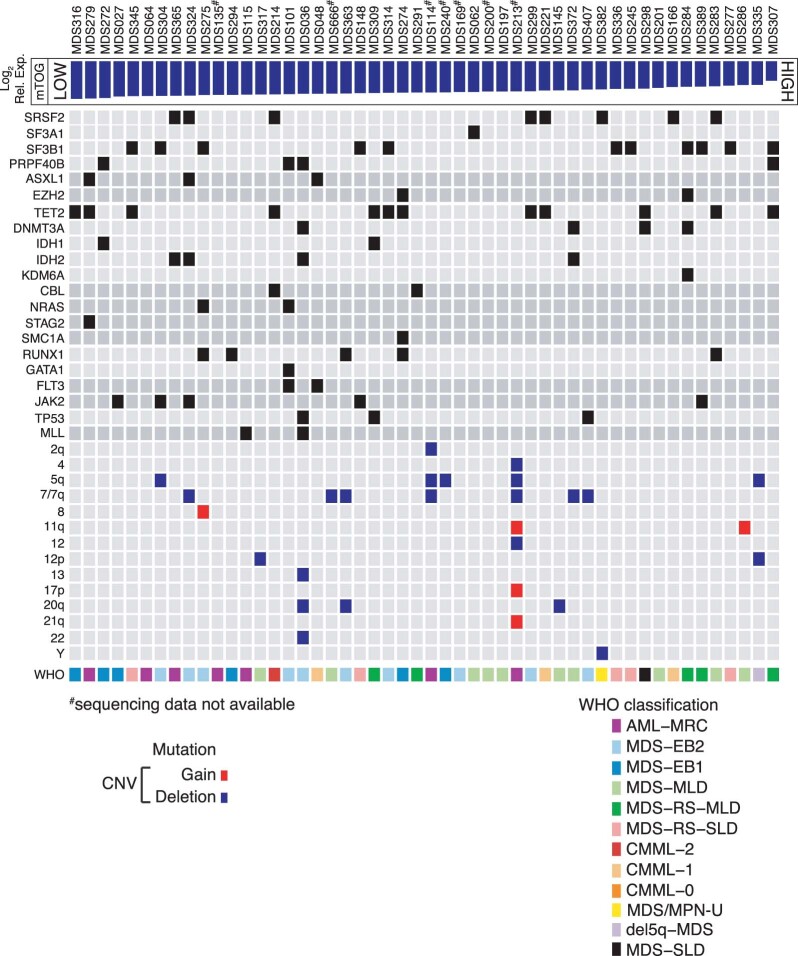
mTOG expression is independent from recurrent mutations and cytogenetic abnormalities in MDS. Mutational landscape of the MDS patient cohort (*n*=50) used for this study. Patients are stratified based on mTOG levels from low to high. mTOG levels were independent from recurrent MDS genetic abnormalities (Mann-Whitney U test). Source data

Dysregulation of PUS7 and mTOG leads to aberrant protein synthesis and impaired haematopoietic differentiation, and may have implications for the pathogenesis of high-risk MDS (HR-MDS) subtypes with chromosome 7 aberrations, including monosomy 7 (−7) or del(7q), that exhibit *PUS7* loss-of-heterozygosity and a high risk of leukaemic transformation^2,26^. Patients with MDS-derived secondary AML (sAML) indeed have an overall poorer prognosis compared with those with de novo AML, highlighting an unmet therapeutic need^27,28^. However, whether dysfunction of PUS7 and mTOG steers protein synthesis to promote leukaemogenesis remains unknown. To establish the clinical implications of PUS7 and mTOG dysfunctions in MDS, we initially assessed transcriptomic data of bone marrow (BM)-derived CD34^+^ HSPCs from the largest patient cohort available to date^29^. Importantly, low PUS7 expression was strongly associated with AML progression (*P* = 0.003; Extended Data Fig. 4a), suggesting broad implications for leukaemogenesis. Hence, we examined mTOG abundance as a direct readout of PUS7 activity in primary HSPCs from 50 patients representing the spectrum of MDS subgroups, including chronic myelomonocytic leukaemia and MDS-derived sAML, with clinical follow-up (Supplementary Table 3). Strikingly, our analysis revealed that low mTOG levels were strongly associated with reduced survival [hazard ratio (HR) = 2.6 and 95% confidence interval (CI), 1.2–5.5; *P* = 0.009] and a drastically increased risk for leukaemic transformation (HR = 10.8 and 95% CI, 1.3–92; *P* = 0.008; Fig. 3a,b). These effects were significant even after exclusion of the −7 and del(7q) cases (Extended Data Fig. 4b,c). Thus, mTOG depletion was superior in predicting a poor disease outcome compared with PUS7 mRNA expression alone, which was more variable between patients (Extended Data Fig. 4d,e). Importantly, mTOG levels were independent of other common genetic alterations in MDS (Extended Data Fig. 5 and Supplementary Table 4) and a robust inverse correlation with blast count was observed (*P* < 0.0001; Fig. 3b, Extended Data Fig. 4f and Supplementary Table 5). A multivariate analysis indicated that mTOG and PABPC1 have independent and additive effects on survival (Supplementary Table 6). Notably, we found a positive correlation (*P* < 0.05) between PAIP1 and the other pathway components—mTOG and PABPC1—in our patient cohort (Extended Data Fig. 4g). Together, these results compellingly suggest that tRF dysfunction may represent a truly unique molecular feature of malignant MDS-HSPCs^30^.

**Extended Data Fig. 6:**
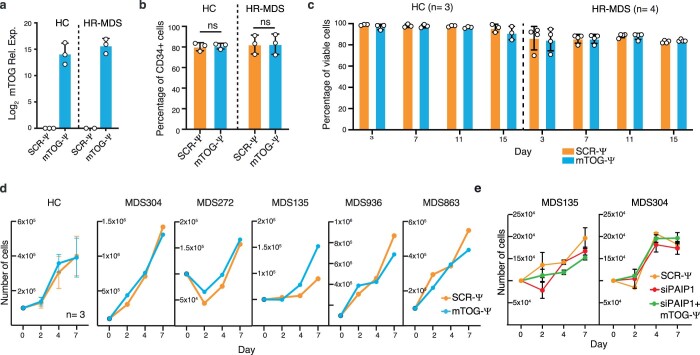
Effects of mTOG-Ψ and siPAIP1 on HC and HR-MDS HSPC *in vitro* expansion. **a**, Graph shows mTOG quantification in HC and HR-MDS HSPC cells after transfection with mTOG-Ψ or SCR-Ψ oligo (3 days). *n*=3 independent biological experiments (except for SCR-Ψ of HR-MDS, where *n*=2) subject to limited patient sample availability. **b**, Graph shows quantification of CD34^+^ cells in HC and MDS-HSPC transduced with mTOG-Ψ or SCR. ns, no statistically significant difference was observed (Welch’s two-tailed t test). *n*=3 independent biological experiments. **c**, Graph shows no changes in HC and MDS-HSPC cell viability in the presence of mTOG-Ψ or SCR-Ψ oligo. **d**, Steady-state HC and MDS-HSPC proliferation is not affected by mTOG-Ψ in culture. Graphs show number of HC and MDS-HSPC at different days in the presence or absence of mTOG-Ψ. **e**, No significant changes in proliferation of HR-MDS HSPC were detected at different days upon SCR-Ψ, siPAIP1 and the combination mTOG-Ψ/siPAIP1 treatment For each patient, *n*=3 independent experimental replicates. Data is shown as mean ± SD. Source data

**Extended Data Fig. 7:**
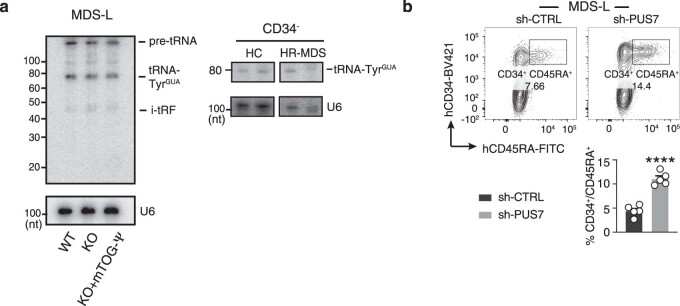
Effects of PUS7 depletion and mTOG-Ψ treatment on tRNA levels in MDS-L and HR-MDS cells. **a**, Northern blot analysis shows no differences in the levels of precursor and mature tRNA-Tyr (GUA) in *PUS7-KD* MDS-L ± mTOG-Ψ (left) and CD34- MNC from two aged-matched HC and HR-MDS patients (bottom right). U6 RNA is included as a loading control. Data represent *n*=3 independent experiments for MDS-L, except when *n*=1 due to limited patient material availability. **b**, Representative FACS analysis shows increase immature MDS propagating cells (CD34^+^/CD45RA^+^/CD123^+^) in *PUS7-KD* MDS-L compared to controls. Graph shows % of CD34^+^/CD45RA^+^/CD123^+^ cells in *CTRL* and *PUS7-KD* MDS-L lines. *n*=5 independent biological replicates, ****p<0.0001 (two-sided t test). Data is shown as mean ± SD. Source data

A major clinical challenge in the management of patients with HR-MDS is progression to AML, which is driven by dysplastic HSPCs with distinct metabolic changes, such as activation of protein synthesis, that render these cells highly sensitive to translation inhibitors^31^. Given that PUS7-mediated pseudouridylation of mTOG is integral to an epitranscriptomic programme that directs translation rates and haematopoietic differentiation both in vitro and in vivo^2^, we conjectured that mTOG-Ψ might target the metabolic and phenotypic properties of MDS-HSPCs. Hence, we delivered synthetic Ψ-modified mTOG and SCR oligos to HSPCs isolated from four distinct patients with HR-MDS and MDS-derived sAML, all characterized by low levels of mTOG and PUS7, and four healthy controls (HC; Fig. 3c, Extended Data Fig. 6a–e and Supplementary Table 7). Critically, mTOG-Ψ selectively modulated protein synthesis in the MDS-HSPC cultures without changes in viability, proliferation or CD34 expression (Fig. 3c and Extended Data Fig. 6b–d). Consistent with previous observations that increased protein synthesis downstream of eIF4F is associated with the activation of distinct mRNA classes, including 5′ PES, and promotes tumorigenesis^17^, we uncovered that translation of mTOG-sensitive transcripts was dramatically increased solely in BM mononuclear cells (BMMCs) derived from patients with HR-MDS and sAML exhibiting reduced levels of mTOG and PUS7 (Fig. 3d and Supplementary Table 6). This included alterations in RPL23 and EIF6 previously implicated in MDS pathogenesis^20–22^ and occurred without transcriptional changes (Fig. 3d). Importantly, increased translation of these 5′ PES-containing mRNAs could be readily reduced following the addition of mTOG-Ψ or siRNA-mediated PAIP1 downregulation in primary HR-MDS BMMCs (Fig. 3e,f). Modulation of the mTOG–PAIP1 axis rescues global and 5′ PES mRNA translation, including RPL29 and EIF6, following short hairpin RNA (shRNA)-mediated *PUS7* KD in the human MDS-derived cell line^32^, MDS-L, which was established from a patient with a chromosome 5q deletion, del(5q), MDS and refractory anaemia with ring sideroblasts. These effects occurred without changes in transcription and tRNA levels (Fig. 3g,h and Extended Data Fig. 7a,b), further highlighting the epistatic interaction between mTOG-Ψ and PAIP1 critical for balancing translation in MDS cells.

**Fig. 4:**
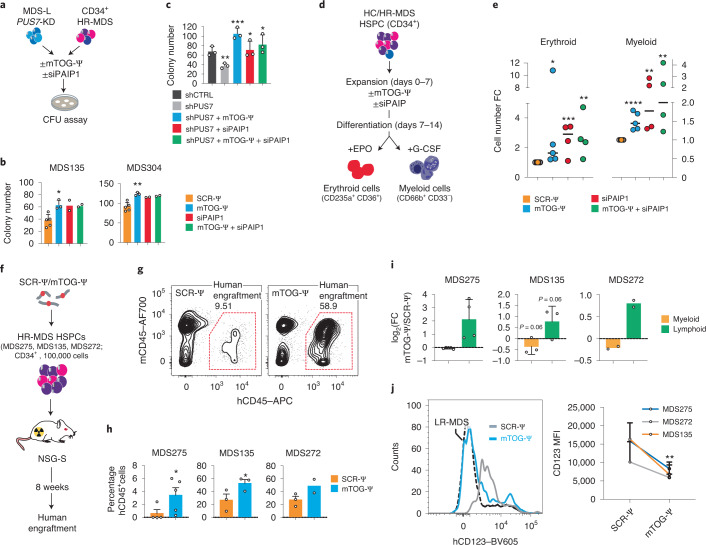
mTOG-Ψ treatment improves differentiation and engraftment of malignant MDS-HSPCs. **a**, Experimental approach employed to examine the effects of mTOG-Ψ and PAIP1 on the CFU potential of *PUS7*-KD MDS-L cells and patient-derived HR-MDS-HSPCs. **b**, Number of colonies obtained from HSPCs from two patients with HR-MDS (MDS135 and MDS304) 15 d following treatment with SCR-Ψ, mTOG-Ψ, siPAIP1, and mTOG-Ψ and siPAIP1. MDS135, *P = 0.0129; and MDS304, **P = 0.0016; one-way ANOVA with multiple comparison; *n* = 2–5 independent biological experiments, indicated as individual dots, subject to material availability. **c**, Number of colonies in shRNA control (shCTRL)-treated and *PUS7*-KD MDS-L cells on day 15 following transduction with SCR-Ψ, mTOG-Ψ, siPAIP1, and mTOG-Ψ and siPAIP1. Data are the mean ± s.d. of *n* = 3 independent biological replicates. **P = 0.0093; ***P = 0.0009; *P = 0.0348 (shPUS7 + siPAIP1) and *P = 0.0197 (shPUS7 + mTOG-Ψ + siPAIP1); two-tailed Student’s *t-*test. **d**, Schematic showing the experimental conditions used for HSPC differentiation in the presence of erythropoietin (EPO) or granulocyte colony-stimulating factor (G-CSF). **e**, Number of differentiated erythroid and myeloid cells, relative to the SCR-Ψ control, obtained following different treatments. The experiments were performed in duplicate or triplicate; *n* = 4 patients, except for mTOG-Ψ, where *n* = 5 patients; each dot represents a patient. Erythoid, *P = 0.047 (mTOG-Ψ), ***P = 0.0003 (siPAIP1), **P = 0.0016 (siPAIP1 + mTOG-Ψ); myeloid, ****P = 0.000012 (mTOG-Ψ), **P = 0.0011 (siPAIP1), **P = 0.0036 (siPAIP1 + mTOG-Ψ); one-tailed Student’s *t*-test. **f**,**g**, Schematic of the HR-MDS-HSPC xenotransplantation experiment (**f**) and representative fluorescence-associated-cell-sorting plots showing the expression levels of human CD45 (hCD45) in the BM of the NSG-S mice (**h**). **g**, The percentages of hCD45 cells in the red gates are shown. **h**, Percentage of human engraftment (hCD45^+^) in mice transplanted with HSPCs from three patients with HR-MDS (MDS275, MDS135 and MDS272). *P = 0.0299 and *P = 0.0283; two-tailed Student’s *t*-test; *n* = 2–5 mice, each dot represents a transplanted mouse. **i**, Changes in the percentage of myeloid (CD33^+^) and lymphoid (CD19^+^) cells in each mTOG-Ψ-treated patient-derived xenotransplant; one-tailed Student’s *t*-test. **j**, Flow cytometric analysis of human CD123 in hCD45^+^hCD34^+^hCD45RA^+^ cells from littermates transplanted with HR-MDS-HSPCs ± SCR-Ψ or mTOG-Ψ (left). The levels of CD123 in the same cell population from an NSG-S mouse transplanted with LR-MDS-HSPCs are shown for comparison (dashed black line). Mean fluorescence intensity (MFI) of CD123 in hCD45^+^hCD34^+^hCD45RA^+^ cells from the patient-derived xenotransplantation experiments (right). **P = 0.0047; two-tailed Student’s *t-*test. **b**,**c**,**h**,**i**, Data are the mean ± s.d. Source data

**Extended Data Fig. 8:**
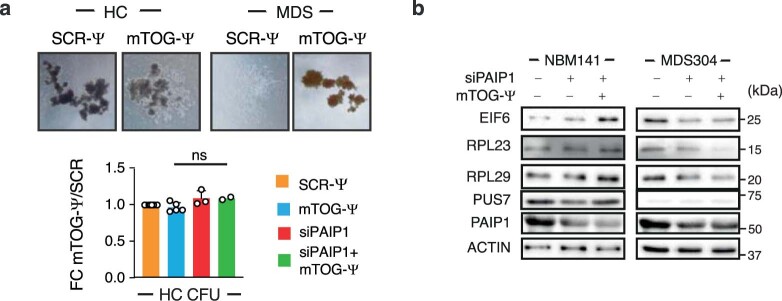
Effects of PUS7 depletion and mTOG-Ψ treatment on HC HSPC colony-forming capacity. **a**, Top, representative images of CFUs obtained using HSPC isolated from HC and HR-MDS patients treated with mTOG-Ψ or SCR-Ψ oligos. Bottom, graph shows FC colony number obtained HC HSPC transduced with SCR-Ψ, mTOG-Ψ, siPAIP1 and the combination mTOG-Ψ/siPAIP1 at day 15. ns, no statistical significance (one-way ANOVA with multiple comparison). Data is shown as mean ± SD. *n*=2-8 patient samples as indicated, dots show individual patients. **b**, Protein analysis show reduced levels of EIF6, RPL23 and RPL29 in CFUs obtained from a HSPC of an HR-MDS patient (MDS304) in the presence of mTOG-Ψ and siPAIP1. In contrast, no differences were detected in CFUs obtained from HC HSPC (NBM141). Data represent two independent healthy and MDS patients in duplicate experiments. Source data

**Extended Data Fig. 9:**
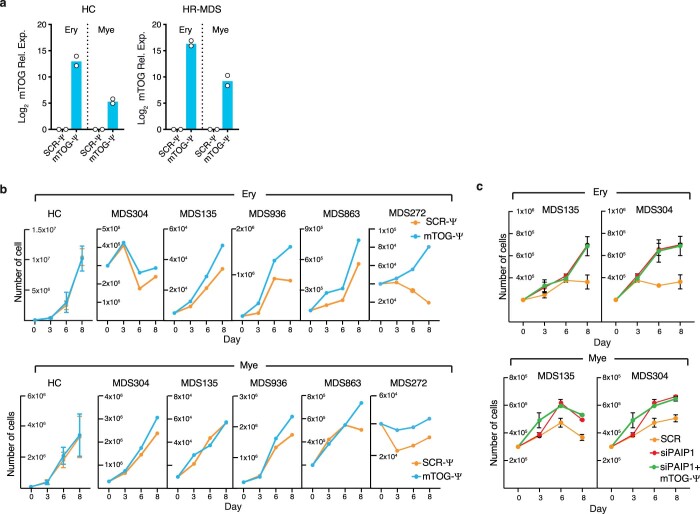
mTOG-Ψ promote HR-MDS HSPC multi-lineage differentiation. **a**, Graph shows mTOG quantification in HC and HR-MDS HSPC cells after transfection with mTOG-Ψ or SCR-Ψ oligo following erythroid (Ery) and myeloid (Mye) differentiation (day 15). Data show two independent biological experiments. **b**, mTOG-Ψ promote expansion of HR-MDS cells in erythroid (top) and myeloid (bottom) differentiation media. No effects were observed in *n*=3 individual HC patient samples, (two-sided t test). Graphs show number erythroid (CD235a+/CD36+) and myeloid (CD66b+/CD33-) cells. during differentiation in the presence or absence of mTOG-Ψ. **c**, PAIP1 downregulation increase erythroid (top) and myeloid (bottom) HR-MDS cells expansion. Differentiation is not further enhanced by co-transfection with mTOG-Ψ oligos. For each patient, *n*=3 independent experimental replicates. Data is represented as mean ± SD. Source data

**Extended Data Fig. 10:**
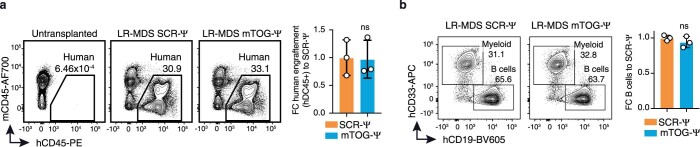
mTOG-Ψ does not affect LR-MDS HSPC engraftment and multi-lineage differentiation. **a**, Representative FACS plots illustrates hCD45 expression in the BM of NSG-S mice untransplanted and transplanted with LR-MDS HSPC ± mTOG-Ψ. Graphs show FC human engraftment to SCR-Ψ-treated mice in 8 weeks post-transplantation (*n*=3 mice). ns, no statistical significance (two-sided t test). **b**, FACS plot shows the expression of myeloid (CD33) and B cell (CD19) markers in the BM of transplanted mice. Graph shows FC lymphoid (CD19^+^) cells to SCR-Ψ in individual mice transplanted with LR-MDS HSPC ± mTOG-Ψ. ns, no statistical significance (two-sided t test). Dots represent individual mice (*n*=3 mice) transplanted with cells from two LR-MDS patient samples. Data is shown as mean ± SD. Source data

Building on previous studies demonstrating that metabolic alteration of protein synthesis affects the function of haematopoietic stem cells and may provide a unique cancer susceptibility in MDS^2,31,33^, we sought to determine whether changes to the levels of mTOG-Ψ and PAIP1 could overcome the differentiation defects in primary patient-derived MDS-HSPCs^27^ (Fig. 4a). Critically, mTOG-Ψ strongly enhanced the colony-forming unit (CFU) potential of patient-derived HR-MDS-HSPCs, with qualitative and quantitative effects, compared with SCR-Ψ oligos and without affecting the differentiation of the HC cells (Fig. 4b and Extended Data Fig. 8a,b). Similarly, PAIP1 downregulation increased the CFU capacity of these primary stem cells, an effect that was not further enhanced with mTOG-Ψ co-treatment, supporting the functional interaction between these factors in HR-MDS cells. Notably, the RPL23, RPL29 and EIF6 protein levels were selectively modulated only in the colonies generated from HR-MDS cells transduced with mTOG-Ψ and siRNA to PAIP1 (siPAIP1; Extended Data Fig. 8b). Accordingly, mTOG-Ψ and PAIP1 promoted differentiation in *PUS7*-KD MDS-L cells (Fig. 4c and Extended Data Fig. 7b). We extended these results using differentiation-inducing culture conditions^34^ and observed remarkable mTOG-Ψ- and PAIP1-dependent expansion of erythroid (CD235a^+^CD36^+^) and myeloid cells (CD66b^+^CD33^−^) for all tested patient-derived HR-MDS-HSPCs in comparison to controls (Fig. 4d,e). The mTOG-Ψ proliferative effects in differentiation-inducing cultures were consistent between patient samples; however, minor variations in response to mTOG-Ψ and siPAIP1 treatment, indicative of individual genetic and clonal heterogeneity, were also observed^35^ (Extended Data Fig. 9a–c). To further demonstrate the specific contribution of mTOG-Ψ towards MDS pathophysiology in vivo, we performed xenotransplantation using HR-MDS-HSPCs with mTOG levels below median that were derived from three different patients (Fig. 4f and Supplementary Tables 5,7). The cells were transfected with mTOG-Ψ or SCR-Ψ oligos and injected into sub-lethally irradiated immunocompromised NSG-S mice (Fig. 4f), which support human MDS engraftment with clinically relevant myeloid differentiation profiles^36^. Strikingly, mTOG-Ψ transfection robustly enhanced human engraftment (approximately twofold) in the BM of the mice injected with HSPCs from the three patients with HR-MDS at 8 weeks post-transplantation compared with the SCR-Ψ-injected group (Fig. 4g,h). Furthermore, the mTOG effects were specific to HR-MDS specimens and were accompanied by a significant reduction (approximately twofold) in malignant CD123^+^ stem and progenitor cells (CD34^+^CD45RA^+^CD123^+^) as well as improved non-leukaemic multi-lineage differentiation with a predominant increase in lymphoid (CD19^+^) but not myeloid (CD33^+^) cells for all tested patients (Fig. 4i,j). Conversely, mTOG-Ψ did not affect the engraftment of low-risk (LR)-MDS-HSPCs (Extended Data Fig. 10a,b). These results indicate that mTOG-Ψ may selectively target a population of CD123^+^ leukaemic stem and progenitor cells characterized by abnormally high levels of protein synthesis associated with increased expression of translation-related gene pathways^31^.

This study unravels a key function for mTOG and Ψ in governing stem cell-associated translation programmes, further highlighting the importance of PUS7-mediated Ψ for translation control during development and leukaemogenesis. Homeostatic modulation of protein synthesis has a paramount role in haematopoietic stem cell differentiation and transformation^33,37^. In this context, mTOG-driven translational repression may critically counteract the metabolic changes involved in MDS-to-AML progression^31,38,39^. This is mediated through the selective control of translation-associated gene-expression programmes that may sustain high protein synthesis rates in subpopulations of MDS and leukaemic stem cells^31,40^. Future work will be required to decipher the oncogenic translational programmes, including specific downstream effector genes governed by PUS7 and mTOG and the impact on MDS aetiology. In summary, our results suggest that uncovering the contribution of tRF epitranscriptomic modifications may hold great promise for future novel therapeutic strategies in haematological malignancies characterized by protein synthesis and stem cell dysfunctions.

## Methods

Experimental procedures involving animals were approved by the Lund University Ethical Committee (Dnr. 5.8.18-02978/2020). Patients were enrolled at the Karolinska University Hospital with informed consent. Specimens were collected and analysed according to the ethical approval by ethical committees for clinical research in Sweden.

### Patients and samples

The patients with MDS and MDS-derived sAML (*n* = 53) participating in this study were enrolled at the Karolinska University Hospital, Stockholm, Sweden at diagnosis or first referral. Informed consent was obtained from all patients. Clinical data from electronic charts were reviewed and updated. All diagnoses were reclassified according to the 2016 revision to the WHO classification of myeloid neoplasms and acute leukaemia and risk-classified according to the revised International Prognostic Scoring System (IPSS-R)^41^. Clinical characteristics including demographic data, diagnosis onset, cytogenetic abnormalities, complete blood counts, disease-modifying treatment (that is, allogeneic transplantation) and survival were also included in the statistical analyses. A detailed description of the patient cohort used for the expression analysis is included in Supplementary Table 3. Nine healthy individuals donated BM samples for use as controls in the functional experiments.

### Mouse strains

NSG-S mice were purchased from the Jackson laboratory and maintained in individually ventilated cages under controlled climate and enriched environmental conditions. The animals had unlimited access to sterile food and water. Four males and 22 females (11–14 weeks old) were used for the xenotransplantation experiments. Littermates of the same sex were randomized to experimental groups.

### Cell culture

H9 hESCs with a normal 46, XX karyotype were acquired from the WiCell Research Institute. The H9 cells were cultured in feeder or feeder-free conditions, depending on the experimental procedure, as described elsewhere^2^. MDS-L cells were maintained in RPMI-1640 medium (Thermo Fisher Scientific) supplemented with 10% FBS, 1% penicillin–streptomycin and 15 ng ml^−1^ recombinant human interleukin (IL)-3 (Peprotech). HEK293T cells were purchased from the American Type Culture Collection and maintained in DMEM medium (Thermo Fisher Scientific) with 10% FBS and 1% penicillin–streptomycin. All cells were cultured at 37 °C with 5% CO_2_ and routinely tested for mycoplasma infection (Universal Mycoplasma Detection Kit, American Type Culture Collection).

### Electrophoretic mobility shift assay

Recombinant PABPC1 (20 μM) was incubated with approximately 250 nM radiolabelled mTOG in EMSA binding buffer (20 mM Tris pH 7.5, 2 mM MgCl_2_, 0.1% BSA, 3% Ficoll 400 and 0.01% NP-40). The reaction was set up at room temperature and blocked after 30 min by adding DNA loading buffer (Thermo Scientific). The reactions were run on an 8% TBE gel. The gel was dried and exposed for autoradiography. The *K*_d_ value was determined using an increasing concentration of recombinant PABPC1 (500 nM to 60 μM). For the poly(A) displacement experiment, 15 nM of radioactively labelled 18-nucleotide poly(A) RNA was incubated with 0.5 μM PABPC1 in the absence or presence of 1 μM of cold mTOG-Ψ.

### HDX-MS

HDX-MS was performed on recombinant PABPC1 at a concentration of 2.4 μg μl^−1^ in 50 mM HEPES, 300 mM NaCl, 5% glycerol and 2 mM DDT, pH 7.5 with and without ligand, and 100 μM mTOG-Ψ. The PABPC1 samples, which had been stored at −80 °C, were thawed at room temperature; as a precaution against sample deterioration, all samples were filled in such a way that no samples were left sitting in the autosampler for longer than 12 h. The HDX-MS analysis was performed using automated sample preparation on a LEAP H/D-X PAL platform interfaced to a liquid chromatography with tandem mass spectrometry system, comprising an Ultimate 3000 micro-LC coupled to an Orbitrap Q Exactive Plus mass spectrometer. The control samples consisted of 2 μl PABPC1 and 3 μl H_2_O, and the interaction analysis samples of 2 μl PABPC1 mixed with 3 μl mTOG-Ψ. The samples were diluted with 25 μl of 50 mM HEPES, 300 mM NaCl and 2 mM DDT, pH 7.2 or HDX labelling buffer of the same composition prepared in D_2_O, pH 6.9. The HDX labelling reactions were carried out for *t* = 0, 30, 300, 1,000, 3,000 and 9,000 s at room temperature. The labelling reactions were quenched by dilution with 25 μl of 1% trifluoroacetic acid, 0.4 M TCEP and 4 M urea, pH 2.5 at 1 °C. The quenched samples (50 μl) were directly injected and subjected to online pepsin digestion at 4 °C (Life Technologies; pepsin column, 2.1 × 30 mm). The online digestion and trapping were performed for 4 min using a flow rate of 50 µl min^−1^ in 0.1% formic acid (FA), pH 2.5. The peptides generated by pepsin digestion were subjected to online solid phase extraction on a PepMap300 C18 trap column (1 mm × 15 mm) and washed with 0.1% FA for 60 s. Thereafter, the trap column was switched in-line with a reversed-phase analytical column (Hypersil GOLD; particle size, 1.9 µm; 1 × 50 mm) and separation was performed at 1 °C using a gradient of 5–50% solution B over 8 min and then from 50 to 90% solution B for 5 min; the mobile phases were 0.1% FA (solution A) and 95% acetonitrile with 0.1% FA (solution B). Following the separation, the trap and column were equilibrated at 5% organic content until the next injection. The needle port and sample loop were cleaned three times after each injection with mobile phase 5% MeOH with 0.1% FA, followed by 90% MeOH with 0.1% FA and a final wash of 5% MeOH with 0.1% FA. After each sample and blank injection, the pepsin column was washed by injecting 90 μl of pepsin wash solution (1% FA, 4 M urea and 5% MeOH). To minimize carry-over, a full blank was run between each sample injection. The separated peptides were analysed on a Q Exactive Plus mass spectrometer, equipped with a HESI source operated at a capillary temperature of 250 °C with sheath gas 12, auxilliary gas 2 and sweep gas 1 (au). For undeuterated samples (*t* = 0 s), one injection was acquired using data-dependent MS/MS higher-energy C-trap dissociation for the identification of the generated peptides. For HDX analysis (all labelled samples and one *t* = 0 s sample), MS full scan spectra were collected under the following settings: 70,000 resolution; automatic gain control, 3 × 10^6^; maximum injection time, 200 ms; and scan range, 300–2,000.

### HDX-MS data analysis

PEAKS Studio 8.5 (Bioinformatics Solutions Inc.) was used for peptide identification after pepsin digestion of the undeuterated samples (*t* = 0 s). The search was conducted on a FASTA file with only the PABPC1 sequence; the search criteria were: mass error tolerance, 15 ppm; fragment mass error tolerance, 0.05 Da; variable modifications, oxidation (M), 15.99; and allowing for fully unspecific cleavage by pepsin. Peptides identified by PEAKS with a peptide score value of log(*P*) > 25 and no oxidation were used to generate a peptide list containing the peptide sequence, charge state and retention time for the HDX analysis. The HDX data analysis and visualization were performed using HDExaminer, version 2.5.1 (Sierra Analytics Inc.). The analysis allowed only for unimodal exchange kinetics, and the two first residues of a peptide were assumed to be unable to hold deuteration. Due to the comparative nature of the measurements, the deuterium incorporation levels for the peptic peptides were derived from the observed mass difference between the deuterated and non-deuterated peptides without back-exchange correction using a fully deuterated sample. The HDX data were normalized to 100% D_2_O content, with an estimated average deuterium recovery of 80%. The presented deuteration data are the average of all high- and medium-confidence results. The allowed retention-time window was ±0.5 min. The heat-map settings were uncoloured proline, heavy smoothing and the difference heat map was drawn using the residual plot as significance criterion (±0.5 Da). The spectra for all time points were manually inspected; low-scoring peptides, obvious outliers and peptides with retention-time correction that could not be made consistent were removed. In the performed bottom-up labelling HDX-MS, the structural resolution is limited by the degree of overlap of the peptides generated by pepsin digestion. After manual curation, the HDX analysis is based on 300 peptides in the medium- to high-confidence interval, of which 97 (74.1% sequence coverage) peptides were in the high-confidence interval. The average peptide length was 14.6 ± 7.9 (s.d.) with an average redundancy of 6.9.

### DNA constructs

Codon-optimized full-length human *PABPC1* was cloned into the pET26b(+) plasmid between the Nde1 and Xho1 restriction sites, with an additional introduction of an N-terminal 6×histidine tag, followed by a TEV protease site. The carboxy (C)-terminal histidine tag inherent to the vector was not used. The RRM motifs (1–2, 2–3 and 3–4) were cloned in pET28a with an N-terminal histidine tag using gateway cloning. Codon-optimized full-length PAIP1 was cloned into pET21e-DEST with an N-terminal 6×histidine tag, followed by a TEV protease site. This was followed by a 2×StrepII tag. The C-terminal histidine tag from the vector was not used. Codon-optimized full-length PAIP2 was cloned into pGEX-SG-DEST with an N-terminal glutathione *S*-transferase tag, followed by an HRV-3C protease site.

### Protein expression and purification

All proteins were expressed in *Escherichia coli* BL21(DE3) cells (Thermo Scientific) in LB medium and induced with isopropyl β-d-1-thiogalactopyranoside at 20 °C overnight. For the purification of full-length PABPC1 and the individual RRMs, the cells were lysed by sonication in buffer containing 20 mM Tris pH 8.0, 300 mM NaCl, 5% glycerol and 25 mM imidazole supplemented with protease inhibitors (Roche), 1 mg ml^−1^ lysozyme and 5 µg ml^−1^ DNaseI (GE Healthcare). The proteins were isolated from the cleared lysate by binding to a nickel-charged HiTrap IMAC column (GE Healthcare) and eluted from the column by a linear gradient to the same buffer supplemented with 500 mM imidazole. The tags were then cleaved off by overnight incubation with recombinant TEV protease. Post cleavage, the proteins were diluted to a salt concentration of 100 mM NaCl and loaded on a HiTrap Heparin column (GE Healthcare) equilibrated with buffer containing 20 mM Tris pH 8.0, 100 mM NaCl, 5% glycerol and 2 mM dithiothreitol (DTT). The pooled fractions were concentrated and applied to a Superose 6 10/300 column (GE Healthcare) equilibrated in a buffer containing 20 mM HEPES/NaOH pH 7.0, 300 mM NaCl, 5% glycerol and 2 mM DTT. Following purification, the proteins were concentrated using Amicon centrifugal filter units (Merck Millipore) and used immediately for experiments or flash-frozen in liquid nitrogen and stored at −80 °C. For the purification of PAIP1, cells were lysed by sonication in buffer containing 50 mM HEPES/NaOH pH 7.5, 150 mM NaCl, 5% glycerol and 2 mM DTT. The proteins were eluted in the same buffer containing 5 mM d-desthiobiotin and cleaved with TEV protease. After cleavage, the protein was passed over a nickel-charged IMAC column to remove any uncleaved species and the flow-through was concentrated and flash-frozen. For PAIP2, cells were lysed in the same buffer and the cleared lysate was batch purified on glutathione agarose. The proteins were eluted using 25 mM glutathione and flash-frozen without tag cleavage.

### UV crosslinking

Proteins at 1 µM (final concentration) were mixed with approximately 250 nM (final concentration) of radiolabelled mTOG-Ψ in a buffer containing 20 mM HEPES/NaOH pH 7.5, 50 mM NaCl and 5 mM MgCl_2_ in a total reaction volume of 20 µl. The samples were placed on a pre-cooled rack on ice and irradiated by a 254 nm ultraviolet (UV) light source (UVP Crosslinker, AnalytikJena) at a total energy dose of 2,400 mJ cm^−2^. Subsequently, 2×denaturing protein loading buffer was added and the samples were resolved on a 4–12% NuPAGE bis-Tris gel (Thermo Fisher Scientific). The gel was dried and exposed for autoradiography.

### Pulldown assays

Recombinant 6×histidine-tagged PABPC1 (200 nM final concentration) was incubated with 30 µl of Ni-IMAC MagneZoom beads (A.G. Scientific) in binding buffer (50 mM Tris–HCl pH 8, 300 mM NaCl, 0.01% Tween 20, 10 mM immidazole and 10 mM MgCl_2_) at 4 °C. Following a 30 min incubation, the beads were washed three times with binding buffer. PAIP1/PAIP2 (1 µM final concentration) was then added in pulldown buffer (20 mM Tris–HCl pH 8.0, 50 mM NaCl, 0.01% Tween 20 and 10 mM MgCl_2_) to the PABPC1-coated beads. For the reactions with RNA, 2 µM (final concentration) of the respective oligo was added and the mixture was incubated at 4 °C for 1 h. Finally, the beads were washed four times with binding buffer, the bound proteins were eluted into 1×denaturing protein loading buffer and analysed by SDS–PAGE.

### In vivo PABPC1 co-immunoprecipitation

Protein-A-conjugated Sepharose beads (GE Healthcare) were washed in IP buffer (50 mM Tris–HCl pH 7.4, 150 mM NaCl, 1% NP-40 and 1×protease inhibitor) and incubated overnight with 1 μg PABPC1 antibody (Abcam, ab21060) or IgG control (Abcam) at 4 °C with gentle rotation. A 10 cm plate of hESCs with the indicated genotype was harvested in IP buffer. The cells were lysed for 30 min on ice, centrifuged and the supernatant was pre-cleared by incubating with IgG-pre-coated Protein-A-conjugated Sepharose beads for 2 h at 4 °C with gentle rotation. A volume of 20 μl was recovered and used as the input loading control. The supernatant was recovered and added to anti-PABPC1-coated beads and incubated overnight at 4 °C with gentle rotation. The beads were washed four times in IP buffer. To elute the protein, the beads were boiled in Laemmli buffer containing 10 mM DTT with shaking at 700 r.p.m. The supernatants were recovered and analysed by western blotting.

### mTOG pulldown

Biotinylated mTOG-Ψ and exogenous FLAG-tagged PABPC1 (WT, M161A or the ΔRRM deletion mutants) were co-transfected in a 10 cm plate of HEK293T cells using Lipofectamine 3000 (Thermo Scientific). Before harvesting, the cells were UV-crosslinked at 200 mJ cm^−2^. The cells were lysed in lysis buffer (50 mM Tris–HCl pH 7.4, 100 mM NaCl, 0.5% Triton X-100, 0.5% sodium deoxycholate, 0.1% SDS, 5 mM EDTA and protease inhibitors), sonicated and cleared by centrifugation. The supernatants were added to 25 μl of pre-washed Streptadivin-coated Dynabeads (Thermo Scientific) and incubated for 1 h at 4 °C with gentle rotation. The beads were washed twice in lysis buffer, twice in HS buffer (50 mM Tris–HCl, 1,000 mM NaCl, 0.5% Triton X-100, 0.25% sodium deoxycholate, 1 M urea, 5 mM EDTA and 1 mM DTT) and twice in Wash buffer (20 mM Tris–HCl pH 7.4, 10 mM MgCl_2_ and 0.2% Tween 20), followed by elution through the addition of 40 μl Laemmli buffer containing 10 mM DTT with shaking at 700 r.p.m. The supernatants were recovered and analysed by western blotting. Exogenous PABPC1 was detected using anti-FLAG (Sigma).

### Reporter assays

The full-length 5′ UTR sequence of *RPL23* and *RPL29* containing the 5′ PES motif based on the H1 hESC CAGE database (ENCODE RIKEN CAGE) was cloned upstream of the Fluc gene. Luciferase reporters were co-transfected with a pSV-β-galactosidase plasmid (Promega) as the internal control for transfection, using Lipofectamine 3000 (Thermo Scientific). The cells were washed and harvested using Reporter lysis buffer (Promega) 24 h post transfection. Luciferase and β-galactosidase were measured using the Luciferase (Promega) and β-Galactosidase enzyme (Promega) assay systems, respectively, following the manufacturer’s instruction using a Glow-Max plate reader (Promega). The Fluc-to-β-galactosidase signal ratio was calculated for each sample to normalize for differences in transfection efficiency. Candidate-specific 5′ PES mutagenesis was performed using a Q5 site-directed mutagenesis kit (NEB).

### Ribosome profiling

Ribosome profiling was performed as previously descibed^15^ using an ARTseq ribosome profiling kit (mammalian; Epicenter) following the manufacturer’s instructions. Briefly, hESCs were cultured to 60% confluency on 10 cm plates. The cells were treated with cyclohexamide (0.1 mg ml^−1^) for 1 min and then harvested in Mammalian polysome buffer. The lysates were split for total RNA and ribosome footprinting. To obtain ribosome footprints, 5 U of ARTseq nuclease was used per *A*_260_ of lysate, which was purified using MicroSpin S-400 columns. Ribosomal RNA was depleted using Ribo-Zero Gold (Illumina) and the resulting mRNA footprints were isolated on a 15% urea PAGE gel. Isolated mRNA fragments were prepared for sequencing as detailed in the ARTseq kit protocol and sequenced on a HiSeq Illumina system (50 cycles single read). The raw sequence data were clipped using the 3′ adaptor sequence AGATCGGAAGAGCACACGTCT by fastx_clipper of FASTX-Toolkit v.0.0.14 (http://hannonlab.cshl.edu/fastx_toolkit/). The sequence reads were aligned to an rRNA reference using Bowtie v.1.1.2 (ref. ^42^). The unaligned reads were collected and the rRNA alignments were discarded to reduce rRNA contamination. TopHat v.2.1.0 (ref. ^43^) was used to align the non-rRNA sequencing reads to hg38. The .bam files from TopHat and a custom well-supported protein-coding gene annotation constructed with one transcript for each gene from GENCODE v.35 were used to determine P-site offsets for the ribosome profiling data, count the number of read alignments and calculate the read densities (reads per kilobase million, RPKM) per gene for exons, 5′ UTRs, coding regions and 3′ UTRs using Plastid^44^. Ribowaltz^45^ was used for quality control of the data, including the percentage of P-sites falling into the annotated transcript regions and the trinucleotide periodicity of ribosome footprints along coding sequences. The TE was measured as the ratio of ribosome footprints (RPKM of coding regions) to mRNA fragments (RPKM of exons). The cutoffs of log_2_(FC TE KO/WT) > 1, |log2FC mRNA| < 1 and statistical significance of FDR ribosome-protected mRNA fragments < 0.2 were used to identify the differently translated genes. 5′ PES motifs were assigned for the first 10 bp of the 5′ UTR sequences; 5′ PES motifs were significantly enriched in the 5′ UTR of up-translated genes with hypergeometric *P* < 0.00001. Codon occupancy for the E, P and A sites was determined using the RiboWaltz function ‘codon_usage_psite’ with the parameter ‘site’ set to ‘esite’, ‘psite’ and ‘asite’, respectively^45^.

### Western blotting

Cells were washed with ice-cold PBS and lysed in ice-cold RIPA lysis buffer (150 mM NaCl, 1% NP-40, 0.5% sodium deoxycholate, 0.1% SDS and 10 mM Tris–HCl pH 8.0) supplemented with phosphatase and protease inhibitor cocktails (Sigma-Aldrich). The lysates were incubated on ice for 30 min, with occasional vortexing, and centrifuged at 12,000 g for 10 min at 4 °C. The supernatants were transferred and assayed for protein concentration using a Quick Start Bradford protein assay kit (Bio-Rad). Equal amounts of proteins (20–80 μg) were subjected to SDS–PAGE and transferred to polyvinylidene fluoride membranes (Bio-Rad). Antibodies to the following proteins were used: PABPC1 (Cell Signaling Technology), LARP1 (Cell Signaling Technology), PAIP1 (Thermo Scientific), PAIP2 (Santa Cruz Biotechnology), RPL29 (Thermo Scientific), PUS7 (Sigma-Aldrich), DHX36 (Protein Tech), FLAG (Sigma-Aldrich) and puromycin (Merck Millipore). Either β-actin (Sigma-Aldrich) or GAPDH (Abcam) were used as a loading control. The antibody dilutions can be found in the Reporting Summary.

### RNA stability

Human ESCs were treated with actinomycin-D (5 μg ml^−1^) for the indicated time periods. RNA was harvested in TRIzol and extracted using Direct-zol miniprep columns (Zymo Research). The RNA was treated with Turbo DNase (Thermo Scientific), retrotranscribed using high-capacity reverse transcriptase (Thermo Scientific) and quantitative PCR was performed using SYBR Green supermix (Bio-Rad). The mRNA levels were normalized to *18S* rRNA.

### Protein stability

To halt translation elongation, hESCs were treated with cycloheximide (50 μg ml^−1^) for the indicated time. The cells were then harvested in RIPA lysis buffer and proteins were extracted and analysed by western blotting.

### siRNA- and shRNA-mediated gene KD

For the KD experiments, hESCs, MDS-L and CD34^+^ cells were transfected with 20 nM of a mixture of four siRNAs targeting *PAIP1* or *PABPC1* (ON-TARGETplus, Dharmacon) using RNAiMAX reagent (Thermo Fisher) on the day of plating. Control cells were transfected with 20 nM of non-targeting siRNA. After transfection (48 h), the cells were harvested according to the experimental procedure. For lentiviral shRNA, control and *PUS7* shRNA oligos were purchased (Eurofins) and subcloned into pLKO.1 TRC U6-shRNA PGK-eGFP lentiviral vectors^2^. MDS-L cells were transduced twice every 24 h with lentiviral particles in the presence of polybrene (4 μg ml^−1^; Santa Cruz Biotechnology).

### Global protein synthesis measurements

The protein synthesis rate was determined as previously described using either ^35^S radioactive metabolic labelling or a Puromycin incorporation assay^2^. Briefly, for ^35^S methionine/cysteine labelling, cells were starved in methionine-free media supplemented with 10% dialysed FBS for 45 min. After starvation, the cells were incubated for 40 min in starvation medium supplemented with 30 μCi ml^−1^ protein labelling mix (EasyTag protein labeling mix, Perkin Elmer). For the puromycin incorporation assay, the cells were treated for 30 min in culture medium supplemented with 1 μM puromycin. After treatment, the cells were harvested, lysed in RIPA buffer and the labelled proteins were run on an SDS–PAGE gel and blotted on a polyvinylidene fluoride membrane. The membranes were either exposed to autoradiography films (GE Healthcare) or incubated with anti-puromycin (clone 12D10, Merck Millipore).

### PUS7 quantification

CD34^+^ cells were isolated from BM samples of all patients using MACS columns (Miltenyi Biotec), as previously described^3^, and stored in TRIzol (Thermo Scientific) at −80 °C. Total RNA was extracted using Direct-zol microprep (Zymo Research) according to the manufacturer’s instructions. To quantify the *PUS7* RNA expression levels, complementary DNA was synthesized using a High-capacity cDNA reverse transcription kit (Applied Biosystems). The relative expression levels of *PUS7* were evaluated by droplet digital PCR (Bio-Rad), using TaqMan probes (Thermo Fisher) for *PUS7* (Hs01031425_m1), *PAIP1* (Hs01925976_sl), *PABPC1* (Hs00743792_sl) and normalized to *HPRT1* (Hs02800695_m1) expression. More specifically, droplets were prepared according to the manufacturer’s instructions on a QX200 droplet generator (Bio-Rad). Emulsified PCR reactions were run on a thermal cycler (Bio-Rad) where the plates were incubated at 95 °C for 10 min, followed by 40 cycles at 94 °C for 30 s and 60 °C for 60 s, and a 10 min incubation at 98 °C. The temperature ramp increment was 2.5 °C s^−1^ for all steps. The plates were read on a QX200 droplet reader (Bio-Rad) and the results were analysed using the QuantaSoft v.1.5.38.1118 software (Bio-Rad). The QuantaSoft software was used to calculate the ratio of *PUS7*, *PAIP1* and *PABPC1* relative to *HPRT1*.

### mTOG quantification

The mTOG levels were quantified using stem-loop retro-transcription (RT) quantitative PCR (RT–qPCR) as described previously^2^. Briefly, RNA was treated with Turbo DNase (Invitrogen) as per the manufacturer’s instructions. The RNA was quantified by spectrophotometry (Nanodrop) and 120 ng were used for the RT reaction. The RNA was incubated with 50 nM mTOG-specific RT primer, 50 nM miR16-specific RT primer and 250 μM dNTPs (Invitrogen) at 65 °C for 5 min (the indicated concentrations are the final concentrations in the RT reaction). The reaction was placed on ice for 2 min and RT mix was added to each sample (2.5 U μl^−1^ Superscript III (Thermo Fisher), 0.2 U μl^−1^ RNaseOUT (Thermo Fisher), 5×First-strand buffer (Thermo Fisher) and 10 mM DTT (Thermo Fisher)). The reactions were incubated in a thermocycler using the following protocol: 16 °C for 30 min; 60 cycles of 30 °C for 30 s, 42 °C for 30 s and 50 °C for 1 s; and RT inactivation at 80 °C for 5 min. Finally, the cDNA was diluted 1:4 and qPCR was performed using Sso Advanced Universal SYBR Green supermix (Bio-Rad). The raw data for the mTOG quantifications are provided in Supplementary Table 4.

### Northern blotting

Northern blot analysis was performed using 1 μg of total RNA on a 10% TBE–urea gel (Thermo Fisher). The RNA was transferred to a Hybond-N+ membrane (GE Healthcare) post UV crosslinking. The membrane was dried and pre-hybridized at 68 °C for 30 min in PerfectHyb plus hybridization buffer (Sigma) and 0.1 mg ml^−1^ herring sperm DNA (Thermo Fisher). During hybridization, 1 × 10^6^ c.p.m. ml^−1^ of ^32^P-labelled LNA/DNA probe was added^2^. The membrane was hybridized at 68 °C overnight. Subsequently, the membrane was washed once in low stringency buffer (2×SSC and 0.1% SDS) at room temperature for 5 min and twice in high stringency buffer (0.5×SSC and 0.1% SDS) at 68 °C for 20 min. Quantification was performed overnight using a phosphorimager (Fuji film FLA3000). After exposure, the membranes were incubated in boiling stripping buffer (0.1% SDS and 5 mM EDTA) and blotted with U6 probe for the loading control.

### Targeted DNA sequencing

Characterization of recurrent somatic mutation was performed in 43 of 50 cases used for mTOG, *PUS7*, *PABPC1* and *PAIP1* gene-expression analysis (no sequencing data are available for patients MDS114, MDS135, MDS169, MDS200, MDS213, MDS240 and MDS666). A core panel of 42 genes recurrently mutated in myeloid neoplasms was analysed using an Illumina HiSeq platform, and sequence-variant detection, filtering criteria for mutational calling as well as variant allele frequency were performed as previously described^34,46^.

### mTOG transfection in HSPCs

CD34^+^ cells were transfected with 20 nM of SCR or mTOG RNA oligos as previously described^2^. Briefly, the oligos were incubated with Lipofectamine 2000 in IMDM medium for 20 min at room temperature. The mixtures were added dropwise to the cells.

### Measurement of protein synthesis in HSPCs

Protein synthesis was measured using OP-puromycin (Medchem Source) as previously described^6^. Briefly, 2 d following mTOG transfection, CD34^+^ cells were incubated in culturing medium supplemented with 20 μM OP-puromycin for 30 min at 37 °C. After incubation, the cells were fixed in 3.7% formaldehyde in PBS for 15 min at room temperature. The cells were washed and permeabilized in 0.5% Triton X-100 in PBS for 20 min at room temperature. A Click-IT reaction was performed using a Click-IT plus Alexa Fluor 555 picolyl azide toolkit (Invitrogen) following the manufacturer’s instructions. OP-puromycin incorporation was analysed by flow cytometry using LSRFortessa or LSRII (BD Biosciences) flow cytometers. The mean fluorescence intensity was calculated using the FlowJo analysis software and data are presented as the log_2_-transformed value of the FC of mTOG over SCR-transfected cells.

### In culture differentiation of CD34^+^ cells

For the in culture differentiation assay, cells were transfected with mTOG or SCR oligos on days 0 and 7 of the protocol. The differentiation assay was performed as previously described^34^. Briefly, the cells were expanded for 7 d in stem cell medium (IMDM (Sigma), 15% BIT (StemCell Technologies), 1% GlutaMAX (Invitrogen), 10 ng ml^−1^ IL-3 (Peprotech), 10 ng ml^−1^ IL-6 (Peprotech) and 25 ng ml^−1^ SCF (Peprotech); the cells were maintained at a concentration of 0.1 × 10^6^ cells ml^−1^. After 7 d, the cultures were divided for erythroid and myeloid differentiation. Erythroid differentiation of cells at a concentration of 0.2 × 10^6^ cells ml^−1^ was induced using stem cell medium supplemented with 2 U ml^−1^ erythropoietin (Peprotech). Myeloid differentiation of cells at 0.3 × 10^6^ cells ml^−1^ was induced in Myelocult H5100 medium (StemCell Technologies) supplemented with 1 × 10^−6^ M hydrocortison (StemCell Technologies) and 20 ng ml^−1^ G-CSF (Peprotech). Differentiation was evaluated in healthy control BM samples by May–Grünwald Giemsa staining and flow cytometry as a positive readout. For erythroid and myeloid assessment, the following antibodies were used: anti-CD36–PE (1:200; BioLegend), anti-CD235a–PECy7 (1:1,000; BioLegend), anti-CD33–APC (1:200; BioLegend), anti-CD66b–FITC (1:200; BD Biosciences) and anti-CD34–BV421 (1:100; BioLegend).

### Flow cytometric analysis

FACS was performed following standard procedures. Briefly, cells were washed in FACS buffer (PBS + 3% FBS) and stained in the appropriate antibody mix for 30 min on ice in the dark. The cells were washed and analysed on LSRII or LSRFortessa (BD Biosciences) flow cytometers.

### Colony-forming assay

For the colony-forming assay, CD34^+^ cells were transfected with SCR or mTOG oligos on thawing. The following day, the cells were plated in H4435 methylcellulose (StemCell Technologies) soaked with either SCR or mTOG oligos. For NBM, 1 × 10^3^ CD34^+^ cells per replicate were plated in methylcellulose. For the MDS-HSPCs, different cell numbers were plated, ranging from 5 × 10^3^ to 20 × 10^3^ cells, to ensure optimal colony density. The CFU number and cellularity were assessed after 14 d.

### Xenotransplantation

HSPCs (CD34^+^) derived from individuals with LR-MDS and HR-MDS were transfected with 20 nM of SCR or mTOG-Ψ RNA oligos. The cells were harvested 6 h post transfection and 1 × 10^5^ cells were injected into sub-lethally irradiated (250 cGy) NSG-S mice (11–14 weeks old) via the tail vein. Human engraftment was followed in the peripheral blood of the transplanted mice every 2 weeks. After 8 weeks, the mice were killed and their BM cells were harvested, treated with ammonium chloride solution (StemCell Technologies) to lyse the red blood cells, and stained. The following antibodies were used: anti-mouse CD45–Alexa Fluor 700 (1:200; BioLegend), anti-human CD45–APC (1:200; BioLegend), anti-human CD19–BV605 (1:200; BD Biosciences), anti-human CD15–PE (1:200; BioLegend), anti-human CD33–PE (1:200; BD Biosciences), anti-human CD34–BV421 (1:100; BioLegend), anti-human CD123–BV605 (1:50; BioLegend) and anti-human CD45RA–FITC (1:200; Thermo Scientific). The cells were then washed, resuspended in 1 μg ml^−1^ 7-AAD (BioLegend) in PBS + 3% FBS and analysed using an LSRFortessa X-20 flow cytometer (BD Biosciences).

### Statistics and reproducibility

The experiments presented in this study were performed as multiple biologically independent replicates, as indicated in the figure legends, and no inconsistent results were observed. Data are plotted as bar graphs with individual values, the mean ± s.d. and statistical significance, performed using a one-way ANOVA or Student’s *t*-test, unless specified otherwise. Details of the particular statistical analyses used, exact *P* values, number of independent biological replicates and sample sizes for all of the graphs are indicated in the figures or figure legends. The patient cohort used for quantification of mTOGs, PUS7, PAIP1 and PABPC1 was balanced for the various categories of MDS and AML with myelodysplasia-related changes according to the WHO-2016 classification. The mTOG expression levels or risk classifications were used as selection criteria for functional studies. Numerical variables were compared using a Student’s *t*-test or Pearson’s correlation in accordance with variable characteristics, unless otherwise stated. Overall survival was computed from diagnosis to the last follow-up or death. Time to AML progression (that is, leukaemic transformation) was computed from diagnosis to the last follow-up or AML progression. Both the overall-survival and time-to-AML-progression analyses were performed using the Kaplan–Meier method and a log-rank test. Multivariate analyses were performed using Cox proportional hazards regression for overall survival with stepwise selection based on the Akaike information criterion score. All survival analyses accounted for left censoring at the time of molecular assessment and right censoring in the case of a disease-modifying treatment (for example, allogeneic transplantation). No data were excluded. Animals were randomized into experimental groups for all xenotransplantation experiments. The investigators were blinded to the scoring of the CFU assay, otherwise no formal blinding to allocation during experiments and outcome assessment was implemented.

### Reporting Summary

Further information on research design is available in the Nature Research Reporting Summary linked to this article.

## Online content

Any methods, additional references, Nature Research reporting summaries, source data, extended data, supplementary information, acknowledgements, peer review information; details of author contributions and competing interests; and statements of data and code availability are available at 10.1038/s41556-022-00852-9.

## Supplementary information

Supplementary Information Supplementary Fig. 1.

Reporting Summary

Peer Review Information

Supplementary Tables 1–7 Supplementary Table 1. Peptide list related to HDX-MS analysis. Supplementary Table 2. Ribosome profiling data in hESCs. The table reports read counts and RPKM values for total RNA and ribosome-protected fragments (RPF) of translationally upregulated mRNAs in *PUS7*-KO cells. Ribosome profiling data have been deposited in the Gene Expression Omnibus under the accession code GSE162050. Supplementary Table 3. Description of experimental cohort of patients with MDS used for the gene-expression analysis. Supplementary Table 4. Distribution of mTOG levels according to recurrent mutations in MDS. Supplementary Table 5. Quantification of the mTOG levels in HSPCs from the cohort of patients with MDS. Supplementary Table 6. Multivariate Cox model analysis of PUS7, mTOG, PABPC1 and age on outcome in the cohort of patients with MDS. Supplementary Table 7. Description of cohort of patients with MDS and the HCs used for the functional assays.

## Data Availability

Ribosome profiling data that support the findings in this study have been deposited in the Gene Expression Omnibus under the accession number GSE162050. HDX-MS data have been deposited in the PRIDE database with the accession number PXD023122. All other data are available from the corresponding authors on reasonable request. Source data are provided with this paper.

## Source data

Source Data Fig. 1 Unprocessed western blots and/or gels.
Source Data Fig. 1 Statistical source data.
Source Data Fig. 2 Unprocessed western blots and/or gels.
Source Data Fig. 2 Statistical source data.
Source Data Fig. 3 Unprocessed western blots and/or gels.
Source Data Fig. 3 Statistical source data.
Source Data Fig. 4 Statistical source data.
Source Data Extended Data Fig. 1 Unprocessed western blots and/or gels.
Source Data Extended Data Fig. 1 Statistical source data.
Source Data Extended Data Fig. 2 Statistical source data.
Source Data Extended Data Fig. 3 Unprocessed western blots and/or gels.
Source Data Extended Data Fig. 3 Statistical source data.
Source Data Extended Data Fig. 4 Statistical source data.
Source Data Extended Data Fig. 5 Statistical source data.
Source Data Extended Data Fig. 6 Statistical source data.
Source Data Extended Data Fig. 7 Unprocessed western blots and/or gels.
Source Data Extended Data Fig. 7 Statistical source data.
Source Data Extended Data Fig. 8 Unprocessed western blots and/or gels.
Source Data Extended Data Fig. 8 Statistical source data.
Source Data Extended Data Fig. 9 Statistical source data.
Source Data Extended Data Fig. 10 Statistical source data.
