# Supplementary material for: Pseudouridine-modified tRNA fragments repress aberrant protein synthesis and predict leukaemic progression in myelodysplastic syndrome
Source: Nat Cell Biol. 2022 Mar 15;24(3):299–306. doi: 10.1038/s41556-022-00852-9 (PMC8924001; doi:10.1038/s41556-022-00852-9)

nature portfolio

https://doi.org/10.1038/s41556-022-00852-9

Supplementary information

Pseudouridine-modified tRNA fragments repress aberrant protein synthesis and predict leukaemic progression in myelodysplastic syndrome

In the format provided by the authors and unedited

nature portfolio

https://doi.org/10.1038/s41556-022-00852-9

Supplementary information

Pseudouridine-modified tRNA fragments repress aberrant protein synthesis and predict leukaemic progression in myelodysplastic syndrome

In the format provided by the authors and unedited

Supplementary figure: Gating strategies used for the respective panels indicated below

Related to Figure 3c

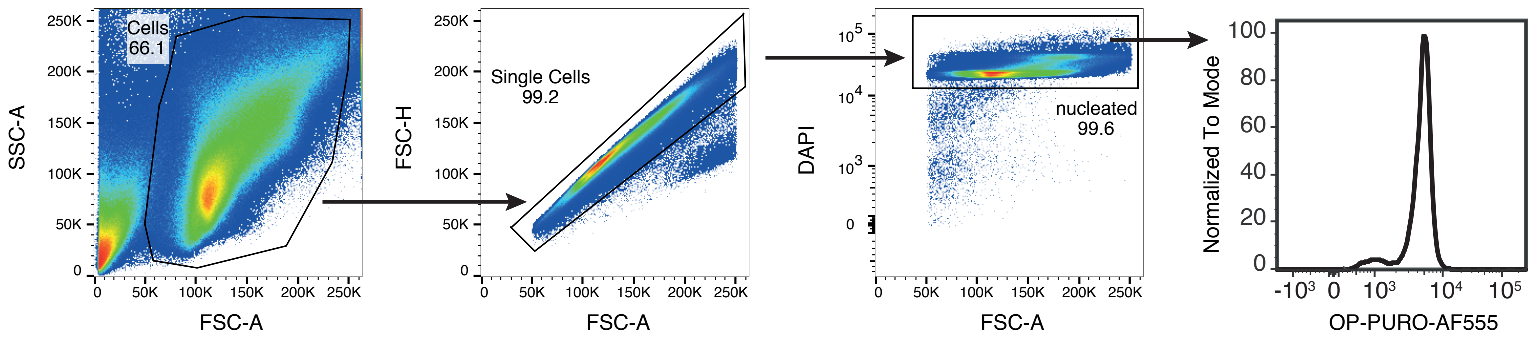

Related to Figure 4e

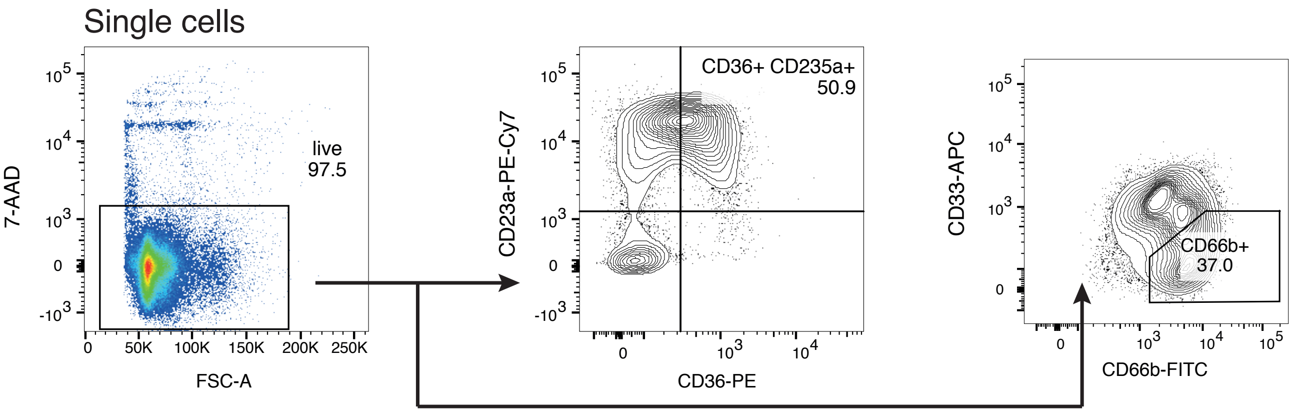

Related to Figure 4g

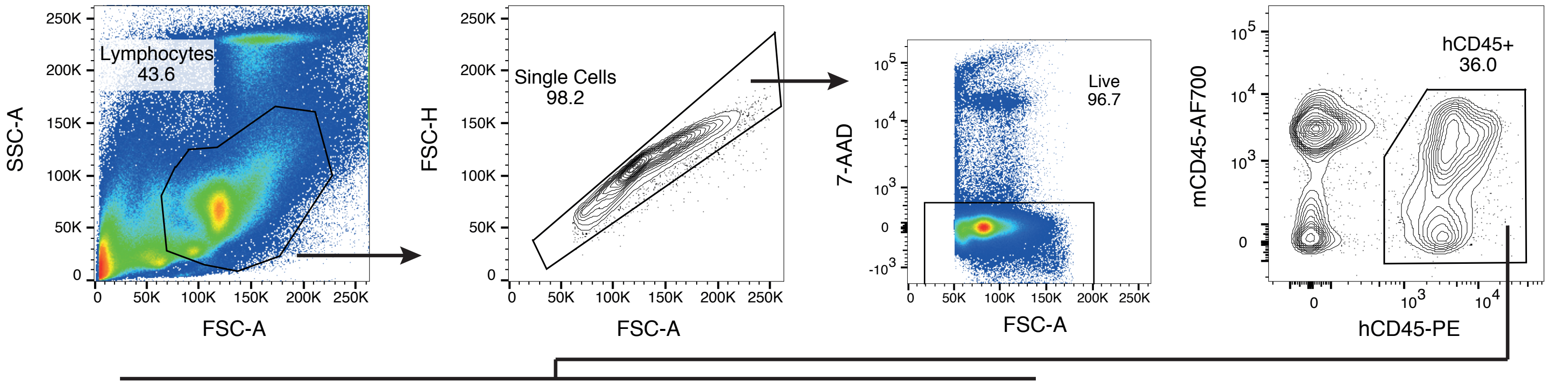

Related to Figure 4i

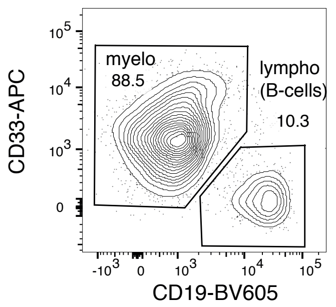

Related to Figure 4j

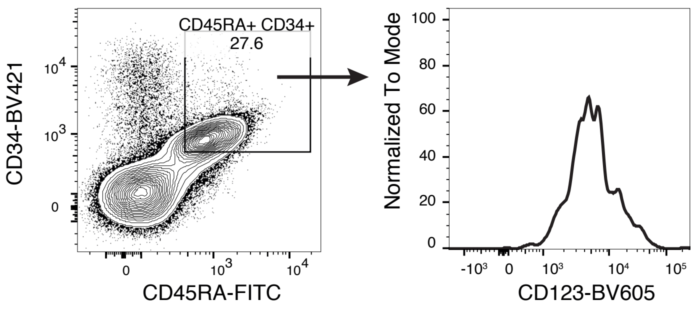

Supplement: Supplementary file 1 — Supplementary Fig. 1. [file 41556_2022_852_MOESM1_ESM.pdf]
