# Supplementary material for: Pseudouridine-modified tRNA fragments repress aberrant protein synthesis and predict leukaemic progression in myelodysplastic syndrome
Source: Nat Cell Biol. 2022 Mar 15;24(3):299–306. doi: 10.1038/s41556-022-00852-9 (PMC8924001; doi:10.1038/s41556-022-00852-9)
Supplement: Supplementary file 2 — Reporting Summary [file 41556_2022_852_MOESM2_ESM.pdf]

nature research

Corresponding author(s): Cristian Bellodi and Marios Dimitriou

Last updated by author(s): Dec 17, 2021

Reporting Summary

Nature Research wishes to improve the reproducibility of the work that we publish. This form provides structure for consistency and transparency in reporting. For further information on Nature Research policies, see our Editorial Policies and the Editorial Policy Checklist.

Statistics

For all statistical analyses, confirm that the following items are present in the figure legend, table legend, main text, or Methods section.

n/a	Confirmed	
☐	☒	The exact sample size (*n*) for each experimental group/condition, given as a discrete number and unit of measurement
☐	☒	A statement on whether measurements were taken from distinct samples or whether the same sample was measured repeatedly
☐	☒	The statistical test(s) used AND whether they are one- or two-sided<br>*Only common tests should be described solely by name; describe more complex techniques in the Methods section.*
☐	☒	A description of all covariates tested
☐	☒	A description of any assumptions or corrections, such as tests of normality and adjustment for multiple comparisons
☐	☒	A full description of the statistical parameters including central tendency (e.g. means) or other basic estimates (e.g. regression coefficient) AND variation (e.g. standard deviation) or associated estimates of uncertainty (e.g. confidence intervals)
☐	☒	For null hypothesis testing, the test statistic (e.g. *F*, *t*, *r*) with confidence intervals, effect sizes, degrees of freedom and *P* value noted<br>*Give P values as exact values whenever suitable.*
☒	☐	For Bayesian analysis, information on the choice of priors and Markov chain Monte Carlo settings
☒	☐	For hierarchical and complex designs, identification of the appropriate level for tests and full reporting of outcomes
☐	☒	Estimates of effect sizes (e.g. Cohen's *d*, Pearson's *r*), indicating how they were calculated

Our web collection on statistics for biologists contains articles on many of the points above.

Software and code

Policy information about availability of computer code

Data collection	Imaging data were collected using ChemiDoc MP Imaging System (Biorad)<br>Ribosome Profiling data were collected using an HiSeq2000 (Illumina)<br>Flow cytometry data were collected using BD LSRFortessa (BD Bioscience), BD LSRFortessa X-20 (BD Bioscience), BD LSRII (BD Bioscience).<br>Data were collected using FACS Diva software v9.0 (BD Bioscience) and analyzed using Flow-Jo v10.5.2<br>Immunofluorescence data were collected using a Confocal Microscope Zeiss LSM 780<br>Mass spectrometry data were collected using an Orbitrap Q Exactive Plus MS (Thermo Scientific)<br>qPCR data were collected using a Real-Time PCR CFX96 (Biorad)<br>Droplet digital PCR data were collected using ddPCR QX200 (Biorad)
Data analysis	HDX-MS data were analyzed as follows: PEAKS Studio 8.5 Bioinformatics Solutions Inc. (BSI, Waterloo, Canada) was used for peptide identification after pepsin digestion of undeuterated samples (t= 0 s.). The search was done on a FASTA file with only the PABPC1 sequence, search criteria was a mass error tolerance of 15 ppm and a fragment mass error tolerance of 0.05 Da Variable Modifications: Oxidation (M): 15.99, and allowing for fully unspecific cleavage by pepsin. Peptides identified by PEAKS with a peptide score value of log P > 25 and no oxidation were used to generate a peptide lists containing peptide sequence, charge state and retention time for the HDX analysis. HDX data analysis and visualization was performed using HDExaminer, version 2.5.1 (Sierra Analytics Inc., Modesto, US). The analysis allowed only for EX2 and the two first residues of a peptide were assumed unable to hold deuteration. Due to the comparative nature of the measurements, the deuterium incorporation levels for the peptic peptides were derived from the observed mass difference between the deuterated and non-deuterated peptides without back-exchange correction using a fully deuterated sample. HDX data was normalized to 100% D2O content with an estimated average deuterium recovery of 80%. The presented deuteration data is the average of all high and medium confidence results. The allowed retention time window was ± 0.5 min. Heat-map settings were uncoloured proline, heavy smoothing and the difference heat-map was drawn using the residual plot as significance criterion (± 0.5 Da). The spectra for all timepoints were manually inspected; low scoring peptides, obvious outliers and peptides were retention time correction could not be made consistent were removed. In the performed bottom-up labeling HDX-MS the structural resolution is limited by the degree of overlap of the peptides generated by pepsin digestion. After manual curation the HDX analysis is based on 300 peptides in the medium to high confidence interval, of which 97 (74.1% sequence coverage)

peptides were in the high confidence interval. Average peptide length was 14.6 ± 7.9 SD and an average redundancy of 6.9.

Ribosome profiling data were analyzed as follows: the raw sequence data were clipped using the 3' adaptor sequence (AGATCGGAAGAGCACACGTCT) by fastx_clipper of FASTX-Toolkit v0.0.14 (http://hannonlab.cshl.edu/fastx_toolkit/). The sequence reads were aligned to an rRNA reference using Bowtie v1.1.2. The unaligned reads were collected while the rRNA alignments were discarded to reduce rRNA contamination. TopHat v2.1.0 was used to align the non-rRNA sequencing reads to hg38. The .bam files from TopHat and well-supported protein coding gene annotation from GENCODE v35 with transcript support level 1 and 2 were used to determine P-site offsets for the ribosome profiling data, count the number of read alignments, and calculate read densities (RPKM) per gene for exons, 5’ UTRs, coding regions, and 3’ UTRs by Plastid. Ribowaltz was used for quality control the data including the percentage of P-sites falling into the annotated transcript regions and the trinucleotide periodicity of ribosome footprints along coding sequences. Translational efficiency (TE) was measured by the ratio of ribosome footprints (FPKM of coding regions) to mRNA fragments (FPKM of exons). 5’PES motifs were assigned for first 10bp of 5’-UTR sequences. 5’PES motifs were significantly enriched in 5’-UTR of up-translated genes with hypergeometric p value < 0.00001

PUS7, PAIP1 and PABC1 quantification in MDS-derived HSPC: PUS7, PAIP1 and PABC1 relative expression was evaluated by droplet digital PCR (Biorad), using TaqMan probes (Thermo Fisher) for PUS7 (Hs01031425_m1), PAIP1 (Hs01925976_s1), and PABPC1 (Hs00743792_s1) and normalized to HPRT1 (Hs02800695_m1) expression. More specific, droplets were prepared according to manufacturer’s instructions on a QX200 droplet generator (Bio-Rad). Emulsified PCR reactions were run on a thermal cycler (Bio-Rad) incubating the plates at 95 °C for 10 min followed by 40 cycles at 94 °C for 30 sec and 60 °C for 60 sec, followed by 10 min incubation at 98 °C. The temperature ramp increment was 2.5 °C/sec for all steps. Plates were read on a QX200 droplet reader (Bio-Rad) and results analyzed using QuantaSoft v1.5.38.1118 software (Bio-Rad). QuantaSoft software was used to calculate the ratio of PUS7, PAIP1 and PABC1 relative to HPRT1.

For manuscripts utilizing custom algorithms or software that are central to the research but not yet described in published literature, software must be made available to editors and reviewers. We strongly encourage code deposition in a community repository (e.g. GitHub). See the Nature Research guidelines for submitting code & software for further information.

Data

Policy information about availability of data

All manuscripts must include a data availability statement. This statement should provide the following information, where applicable:
- Accession codes, unique identifiers, or web links for publicly available datasets
- A list of figures that have associated raw data
- A description of any restrictions on data availability

Ribosome profiling (Ribo-seq) data that support findings in this study have been deposited in the Gene Expression Omnibus (GEO) under the accession number GSE162050. HDX-MS data have been deposited in PRIDE with the accession no. PXD02312. Source data for all the Figs and Extended Data Figs are presented with the paper.

Field-specific reporting

Please select the one below that is the best fit for your research. If you are not sure, read the appropriate sections before making your selection.

☒ Life sciences ☐ Behavioural & social sciences ☐ Ecological, evolutionary & environmental sciences

For a reference copy of the document with all sections, see nature.com/documents/nr-reporting-summary-flat.pdf

Life sciences study design

All studies must disclose on these points even when the disclosure is negative.

Sample size

For mice experiments number of mice is indicated in corresponding figure.
For cell lines experiments, approx. 15-20 million cells were used per replicate per ribosome profiling experiment. 20 million cells were used for endogenous PABPC1 pulldown. 1 million cells were used for western blot and metabolic labeling experiments.

The patient cohort consisted of 53 patients and was balanced for the various MDS and AML-MRC categories according to WHO-2016 classification.

For experiment using MDS patient-derived cells: for CFU different density of CD34+ cells were plated in a range between 5000 to 20000. For in vitro differentiation 100000 CD34+ cells were used at the start of the protocol. For xenotransplantation, 100000 CD34+ cells were injected per mouse replicate.

The sample size was predetermined in accordance to published standards, availability and 3Rs rule for animals in case of in vivo experiments. Sample size is indicated in the figure legends, supplementary methods and text for all experiments.

Data exclusions

No data were excluded.

Replication

The experiments shown in this study were performed as multiple biologically independent replicates, as indicated in the figure legends, and no inconsistent results were observed. Details of the particular statistical analyses used, exact p values, statistical significance, number of independent biological replicates and sample sizes for all of the graphs are indicated in the figures or figure legends. For the in vivo experiments, multiple animals per group were used.

Randomization

22 females and four males (age 11-14 weeks) were used for the xenotransplantation experiments. Littermates of the same sex were randomly selected to experimental groups.

	All relevant information on the MDS/sAML patients cohort included in the study can be found in the Supplementary Table 3, Supplementary Table 7, and relevant parts of the Methods section ('Patients and samples'). The patient cohort used for quantification of mTOGs, PUS7, PAIP1 and PABC1 was balanced for the various MDS and AML-MRC categories according to WHO-2016 classification. As indicated in relevant manuscript sections, patient selection for functional experiment was based on mTOG level and/or risk classification.
Blinding	Colony counts was double-blinded. For all the other experiments, cell type and treatment were known, as information of material was required for performing the experiment and analysis.

Reporting for specific materials, systems and methods

We require information from authors about some types of materials, experimental systems and methods used in many studies. Here, indicate whether each material, system or method listed is relevant to your study. If you are not sure if a list item applies to your research, read the appropriate section before selecting a response.

Materials & experimental systems

n/a	Involved in the study
☐	☒ Antibodies
☐	☒ Eukaryotic cell lines
☒	☐ Palaeontology and archaeology
☐	☒ Animals and other organisms
☐	☒ Human research participants
☒	☐ Clinical data
☒	☐ Dual use research of concern

Methods

n/a	Involved in the study
☒	☐ ChIP-seq
☐	☒ Flow cytometry
☒	☐ MRI-based neuroimaging

Antibodies

Antibodies used

Western blot: anti-human PAIP1 1:800 (Thermo Scientific, PA5-41818), anti-human PABPC1 1:1000 (CST, 4992), anti-human LARP1 1:1000 (CST, 14763S), anti-human Actin 1:2000 (Sigma, A1978), anti-human RPL29 1:5000 (Thermo Scientific, PA5-27545), anti-human RPL23 1:1000 (Protein Tech, 16086-1-AP), anti-human PUS7 1:500 (Sigma, SAB4100174), anti-FLAG 1:1000 (Sigma, F1804), anti-human DHX36 1:1000 (Protein Tech, 13159-1-ap), anti-human PAIP2 1:200 (Abcam, ab237803), mouse anti Puromycin 1:1000 (Merck Millipore clone 12D10, MABE343), rabbit anti-GAPDH 1:1000 (Sigma Aldrich, G9545-100UL).

Pulldown: anti-human PABPC1 (also used for immunofluorescence (Abcam, ab21060)), anti FLAG magnetic beads (Sigma, M8823), anti Streptadivin magnetic beads (Termo Scientific, 65001)

Flow cytometry: anti-mouse CD45-AF700 (BioLegend, 103128), anti-human CD45-PE (BioLegend, 304012), anti-human CD34-BV421 (BioLegend, 343610), anti-human CD45RA-FITC (Termo Scientific, MHCD45RA01), anti-human CD123-BV605 (BioLegend, 306026), anti-human CD15-PE (BioLegend, 301906), anti-human CD33-PE (BD Bioscience, 555450), anti-human CD19-BV605 (BD Bioscience, 562653), Click-iT™ Plus Alexa Fluor™ 555 Picolyl Azide Toolkit (Thermo Scientific, C10642), anti-human CD33-APC (Thermo Scientific, 17-0338), anti-human CD66b-FITC (BD Bioscience, 561927), anti-human CD36-PE (BioLegend, 336205), anti-human CD235a-PE-Cy7 (BioLegend, 349112).

Validation

PABPC1: Cell Signalling Technology, 4992 antibody has been validated in 34 publications (https://www.cellsignal.com/products/primary-antibodies/pabp1-antibody/4992)
LARP1: Cell Signalling Technology, 14763 has been validated in 2 publications (https://www.cellsignal.com/products/primary-antibodies/larp1-antibody/14763)
PAIP1: Thermo Scientific, PA5-41818 has been validated for use in western blots (https://www.thermofisher.com/antibody/product/PAIP1-Antibody-Polyclonal/PA5-41818)
RPL29: Thermo Scientific, PA5-27545 has been validated for use in western blots (https://www.thermofisher.com/antibody/product/RPL29-Antibody-Polyclonal/PA5-27545)
PUS7: Sigma-Aldrich, SAB4100174 antibody has been validated in 6 publications (https://www.sigmaaldrich.com/catalog/product/sigma/sab4100174?lang=en&region=DK)
beta-ACTIN: Sigma-Aldrich, A1978 antibody has been validated in 2722 publications (https://www.sigmaaldrich.com/catalog/product/sigma/a1978?lang=en&region=DK)
PAIP2: Santa Cruz Biotechnology, sc-365317 has been validated for use in western blots (https://www.scbt.com/p/paip2-antibody-a-2)
DHX36:Protein Tech, 13159-1-ap has been validated in 5 publications (https://www.ptglab.com/products/DHX36-Antibody-13159-1-AP.htm)
FLAG: Sigma Aldrich, F3165 has been validated in 4990 publications (https://www.sigmaaldrich.com/catalog/product/sigma/f3165?lang=en&region=DK)
Anti Puromycin: Merck Millipore clone 12D10 , MABE343 has been validated in 231 publications (https://www.sigmaaldrich.com/catalog/product/mm/mabe343?lang=en&region=DK)
GAPDH: Sigma Aldrich, G9545-100UL has been validate in 46 publications (https://www.sigmaaldrich.com/SE/en/product/sigma/g9545?gclid=Cj0KCQiAnuGNBhCPARIsACbnLzqPpdpviw5mON8lJ2fCXAXinX4dZ-YAutlf2RNzlCy_IgNqy3xGPSYaAjlOEALw_wcB)

mouse CD45-AF700: Clone 30-F11, BioLegend 103128 has been validated in 15 publications (https://www.biolegend.com/en-us/products/purified-anti-mouse-cd45-antibody-102?GroupID=BLG1932)
human CD45-PE: Clone HI30, BioLegend 304012 has been validated in 5 publications (https://www.biolegend.com/en-us/search-results/purified-anti-human-cd45-antibody-710)
human CD34-BV421: Clone 561, BioLegend 343610 has been validated in 1 publication (https://www.biolegend.com/en-us/products/

purified-anti-human-cd34-antibody-6034)
human CD45RA: Clone MEM-56, Thermo Scientific MHCD45RA01 (https://www.thermofisher.com/antibody/product/CD45RA-Antibody-clone-MEM-56-Monoclonal/MHCD45RA01)
human CD123: Clone 6H6, BioLegend 306026 (biolegend.com/en-us/products/brilliant-violet-605-anti-human-cd123-antibody-8545?GroupID=BLG2027)
human CD15: Clone HI98, BioLegend 301906 has been validated in 4 publications (https://www.biolegend.com/en-us/products/pe-anti-human-cd15-ssea-1-antibody-713?GroupID=BLG5911)
human CD33: BD Biosciences 555450 has been validated in 5 publications (https://www.bdbiosciences.com/en-us/products/reagents/flow-cytometry-reagents/research-reagents/single-color-antibodies-ruo/pe-mouse-anti-human-cd33.561816)
human CD19: SJ25C1, BD Biosciences 562653 has been validated in 1 publication (https://www.bdbiosciences.com/en-us/products/reagents/flow-cytometry-reagents/research-reagents/single-color-antibodies-ruo/bv605-mouse-anti-human-cd19.562654)
human CD33: Clone WM-53 (WM53), Thermo Scientific 17-0338-41 has been validated in 2 publications (https://assets.thermofisher.com/TFS-Assets/LSG/certificate/Certificates-of-Analysis/17033841_2251965.PDF)
human CD66b: BD Bioscience 561927 (https://www.bdbiosciences.com/en-us/products/reagents/flow-cytometry-reagents/research-reagents/single-color-antibodies-ruo/fitc-mouse-anti-human-cd66b.555724)
human CD36: Clone 5-271, BioLegend 336225 (https://www.biolegend.com/en-us/products/totalseq-a0407-anti-human-cd36-antibody-16651)
human CD235a: Clone HI264, BioLegend 349112 has been validated in 2 publications (biolegend.com/en-us/products/pe-cyanine7-anti-human-cd235a-glycophorin-a-antibody-9003)

In addition western blot antibodies against PABPC1, PAIP1, LARP1 and PUS7 were validated using targeting siRNAs.

Eukaryotic cell lines

Policy information about cell lines

Cell line source(s)	Human embryonic stem cells H9 (WA09) were acquired from the WiCell Research Institute (Madison, Wisconsin, USA) - SLA agreement no. 16-W0062. HEK293t (CRL-3216, ATCC) cells were purchased from ATCC.<br><br>MDS-L cell line was a kind gift from Kaoru Tohyama (Department of Laboratory Medicine, Kawasaki Medical School, Okayama, Japan).
Authentication	Human embryonic stem cells H9 were validated and routinely assessed by alkaline phosphatase staining (Sigma), immunofluorescence analysis of pluripotent marker OCT4, and RT-qPCR analysis of multiple pluripotent factors, as detailed in Guzzi et al., 2018, Cell.<br><br>PUS7-KO hESC clones were screened for editing of the targeting exon, which was confirmed by Sanger sequencing and western blot analysis. Results were published in Guzzi et al., 2018, Cell.
Mycoplasma contamination	All cell lines used in this study were routinely tested and were negative for mycoplasma.
Commonly misidentified lines (See ICLAC register)	No misindentified cell lines were used in this study.

Animals and other organisms

Policy information about studies involving animals; ARRIVE guidelines recommended for reporting animal research

Laboratory animals	NSG-S mice, mus musculus, strain info (The Jackson Laboratory, 013062) , 22 females and 4 males, age 11-14 weeks.
Wild animals	This study did not involved wild animals.
Field-collected samples	The study did not involve samples collected from the field.
Ethics oversight	Experimental procedures involving animals were approved by the Lund University Ethical Committee.

Note that full information on the approval of the study protocol must also be provided in the manuscript.

Human research participants

Policy information about studies involving human research participants

Population characteristics	Consecutive MDS/AML patients (n=53; 50 out of 53 for expression analysis and 10 out of 53 for functional experiments) were enrolled in this study at Karolinska University Hospital, Stockholm, Sweden. Clinical data from electronic charts were reviewed and updated. All diagnoses were reclassified according to the 2016 revision to the WHO classification of myeloid neoplasms and acute leukemia and risk-classified according to the revised International Prognostic Scoring System (IPSS-R). Clinical characteristics including demographic data, diagnosis onset, cytogenetic abnormalities, complete blood count, disease modifying treatment (i.e., allogeneic transplantation), and survival were also included in the statistical analysis. Characteristics of the patients cohorts used for the expression analysis as well as for the functional experiments can be found in Supplementary Table 3 and Supplementary Table 7 respectively.<br>Healthy individuals donated bone marrow (n=9) and used as healthy controls for the functional experiments.

Recruitment	All patients were enrolled in this study at the time of diagnosis or first referral at Karolinska University Hospital, Stockholm, Sweden.
Ethics oversight	Specimens were collected and analyzed according to the ethical approval by ethical committees for clinical research in Sweden.

Note that full information on the approval of the study protocol must also be provided in the manuscript.

Flow Cytometry

Plots

Confirm that:

☒ The axis labels state the marker and fluorochrome used (e.g. CD4-FITC).

☒ The axis scales are clearly visible. Include numbers along axes only for bottom left plot of group (a 'group' is an analysis of identical markers).

☒ All plots are contour plots with outliers or pseudocolor plots.

☒ A numerical value for number of cells or percentage (with statistics) is provided.

Methodology

Sample preparation	For OP-Puro experiment, CD34+ cells were treated with 20uM OP-Puromycin (MedChem Source, JA-1024) for 30 min at 37°C. Cells were washed, fixed, permeabilized and stained as for manufacturer's instruction Click-iT™ Plus Alexa Fluor™ 555 Picolyl Azide Toolkit (Thermo Scientific, C10642). Cells were resuspended in FACS buffer supplemented with 1ug/mL DAPI as viability stain.<br><br>For MDS patient-derived HSPC in vitro differentiation, cells were washed in FACS buffer (PBS, 3% FBS, 1 mM EDTA), and stained with specified antibody cocktail. Cells were resuspended in FACS buffer supplemented with 1ug/mL 7-AAD as viability stain.<br><br>For NSG-S experiment, mice were sacrificed and bone marrow cells were harvested by crushing. Cells were collected and treated with ammonium chloride solution (StemCell Technologies) to lyse red blood cells, for 2 min on ice. Cells were stained using cocktail of specified antibodies for 30 min on ice in dark. Cells were resuspended in FACS buffer supplemented with 1ug/mL 7-AAD as viability stain.
Instrument	Data were collected using the following instruments: BD LSRFortessa (BD Bioscience), BD LSRFortessa X-20 (BD Bioscience), BD LSRII (BD Bioscience)
Software	Data were collected using FACS Diva software v9.0 (BD Bioscience) and analyzed using Flow-Jo v10.5.2
Cell population abundance	No cell sorting was performed in this study
Gating strategy	For OP-Puro experiment, cell singlets were gated using FSC-A/FSC-H and nucleated cells were gated based on DAPI staining. OP-Puro-untreated cells were used as a negative control to set gates.<br><br>For MDS patient-derived HSPC in vitro differentiation, cell singlets were gated using FSC-A/FSC-H, live cells were gated as 7-AAD negative. Cells were then gated based on CD36/CD235a and CD33/CD66b expression for erythroid and myeloid differentiation respectively. Gates were set using FMO controls.<br><br>For MDS-L CD34/CR45RA immunophenotyping, cell singlets were gated using FSC-A/FSC-H, live cells were gated as 7-AAD negative. Extended Data Figure 7b shows CD34/CD45RA plots, gated on live cells.<br><br>For NSG-S experiment, cell singlets were gated using FSC-A/FSC-H, live cells were gated as 7-AAD negative. Human cells were gated on mCD45/hCD45, using untransplanted mice to set positive gates. CD34/CD45ra double positive cells were gated using FMOs. CD123 positive cells were gated using FMOs. For myeloid and lymphoid lineages cells were gated within hCD45-positive cells based on expression of CD33 and CD19.

☒ Tick this box to confirm that a figure exemplifying the gating strategy is provided in the Supplementary Information.
