# Supplementary figures and images for: Pseudouridine-modified tRNA fragments repress aberrant protein synthesis and predict leukaemic progression in myelodysplastic syndrome
Source: Nat Cell Biol. 2022 Mar 15;24(3):299–306. doi: 10.1038/s41556-022-00852-9 (PMC8924001; doi:10.1038/s41556-022-00852-9)

Related to Figure 1

Related to Figure 1a

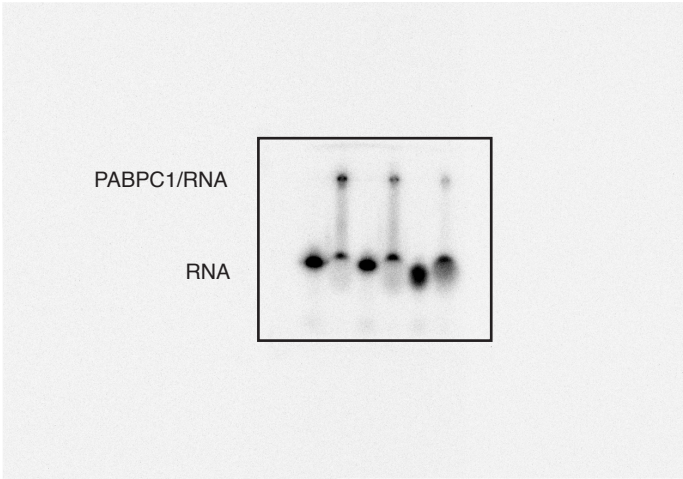

Related to Figure 1c

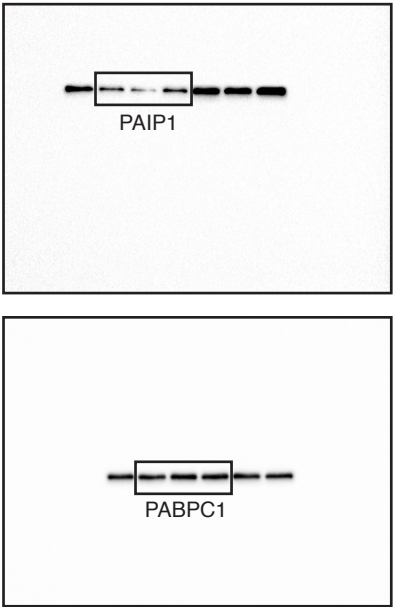

Related to Figure 1d

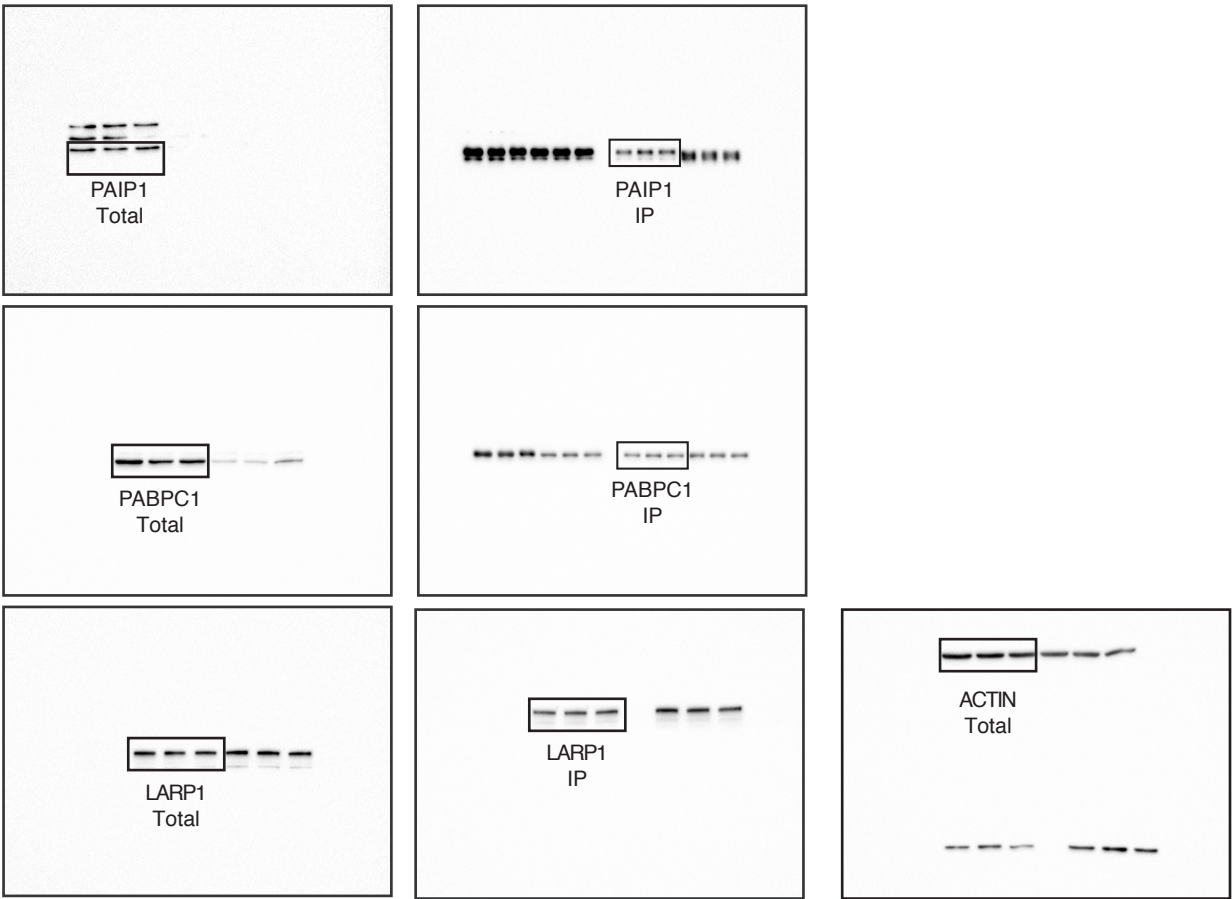

Supplement: Source Data Fig. 1 — Unprocessed western blots and/or gels. [file 41556_2022_852_MOESM5_ESM.pdf]

Related to Figure 2

Related to Figure 2e

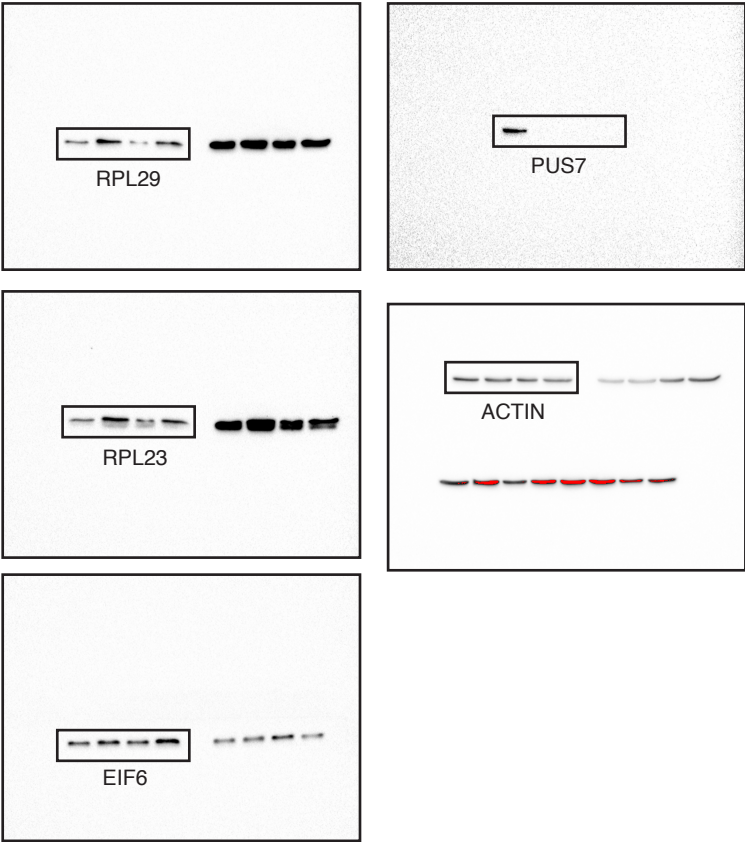

Related to Figure 2f

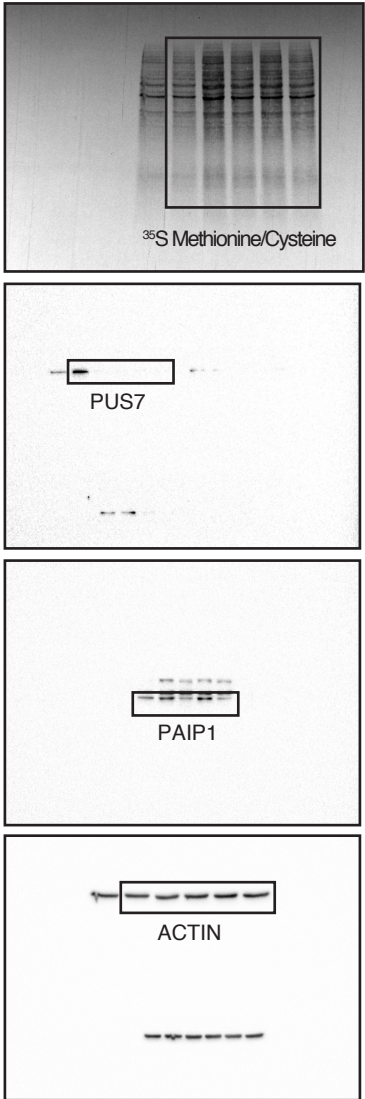

Related to Figure 2g

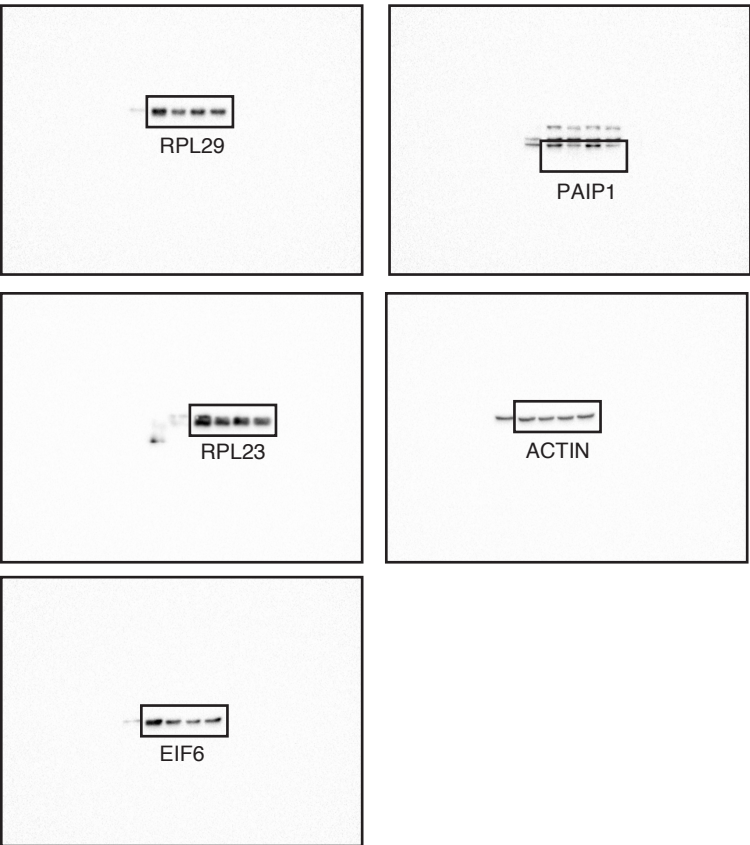

Supplement: Source Data Fig. 2 — Unprocessed western blots and/or gels. [file 41556_2022_852_MOESM7_ESM.pdf]

# Related to Extended data Fig. 1

c

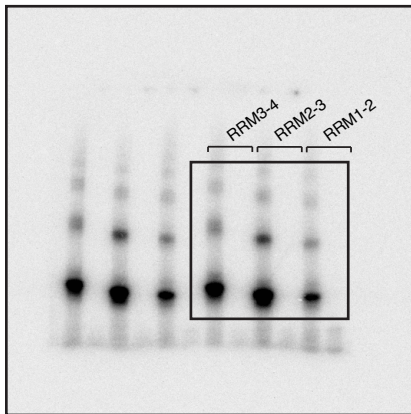

d

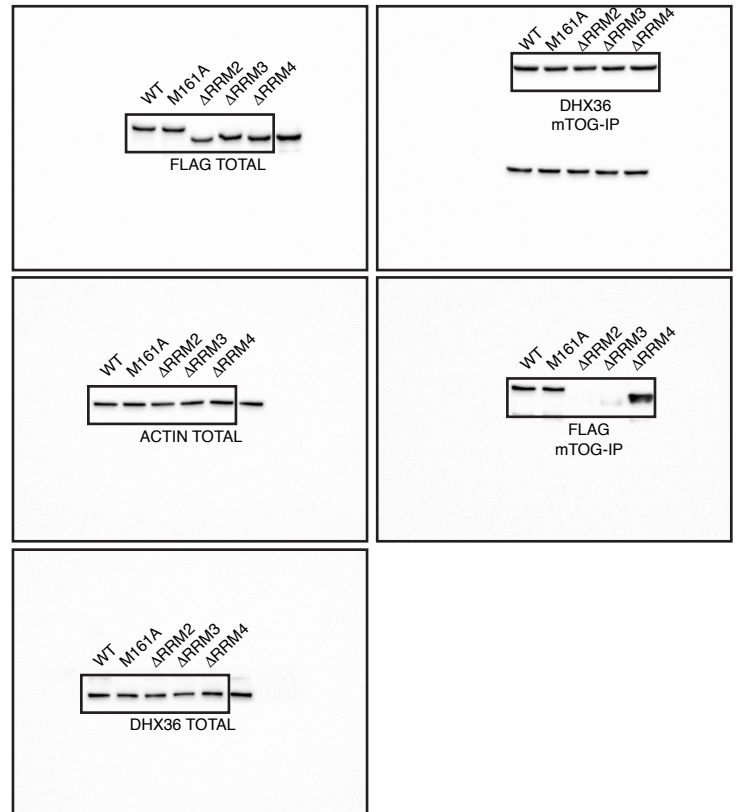

e

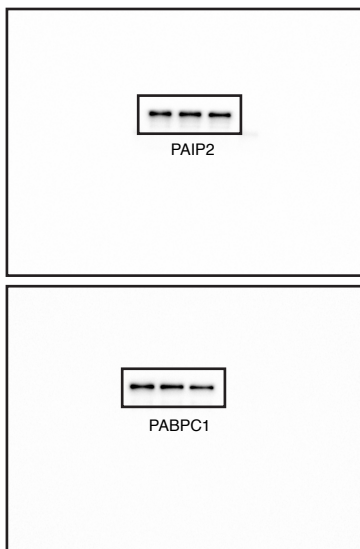

f

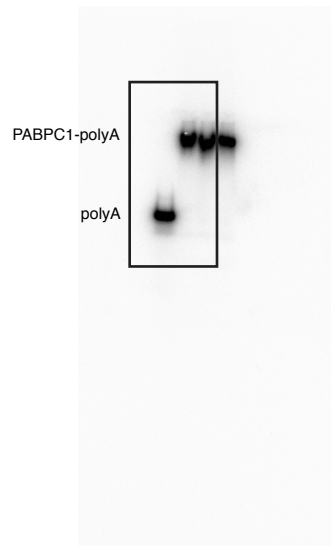

h

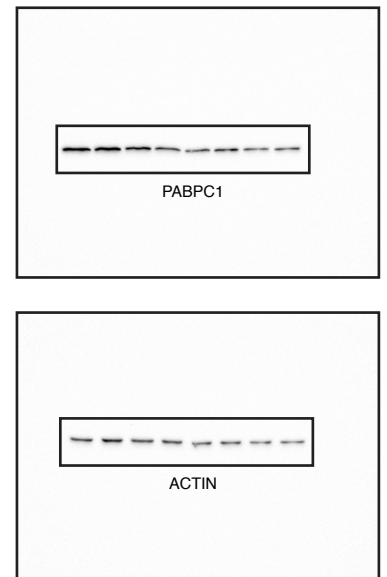

Supplement: Source Data Extended Data Fig. 1 — Unprocessed western blots and/or gels. [file 41556_2022_852_MOESM12_ESM.pdf]

Related to Extended data Fig. 3

a

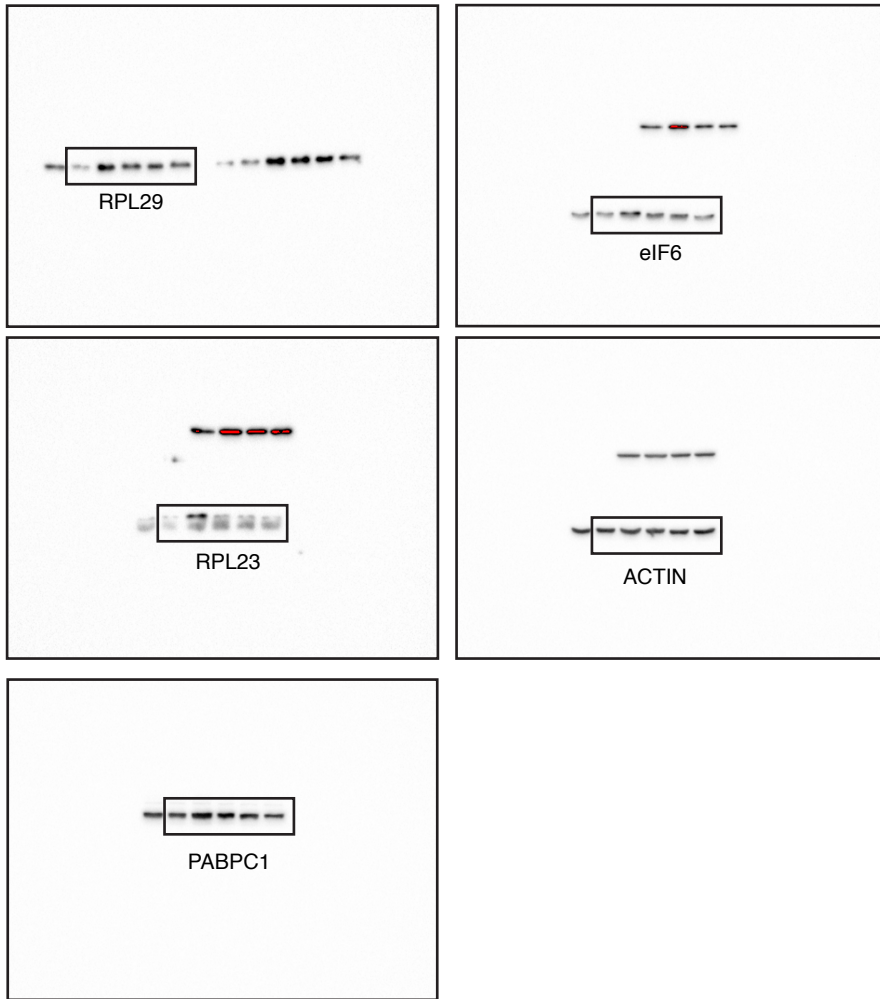

c

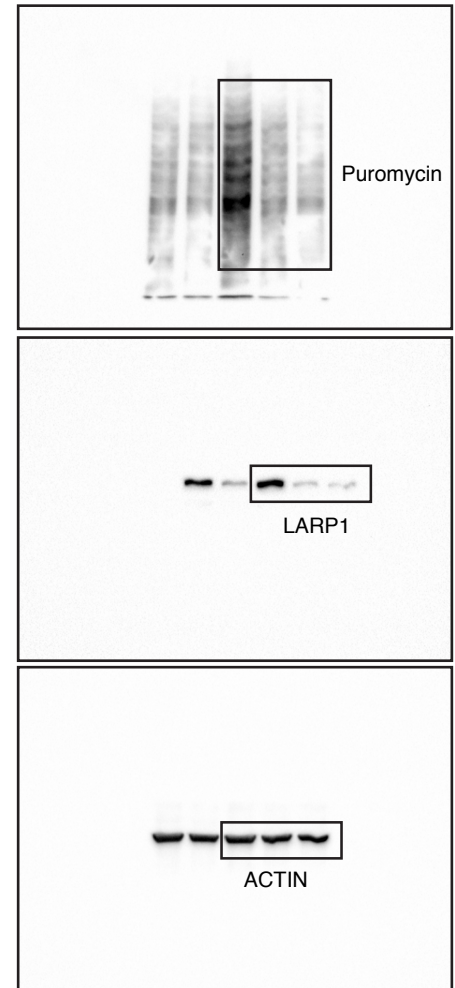

Supplement: Source Data Extended Data Fig. 3 — Unprocessed western blots and/or gels. [file 41556_2022_852_MOESM15_ESM.pdf]

## Related to Extended data Fig. 7

**a**

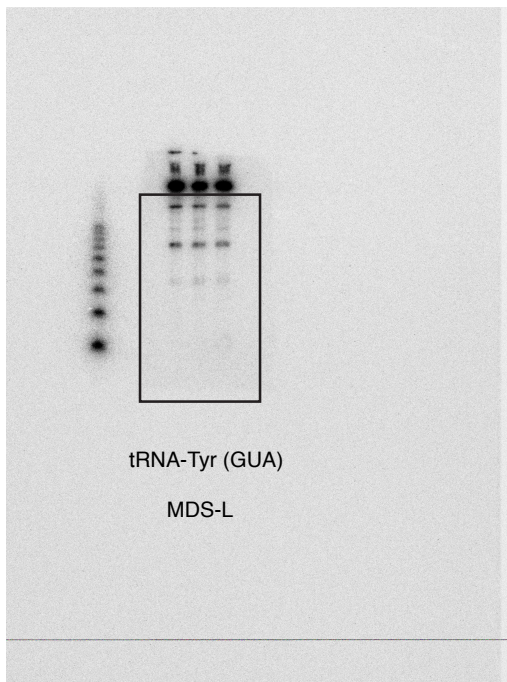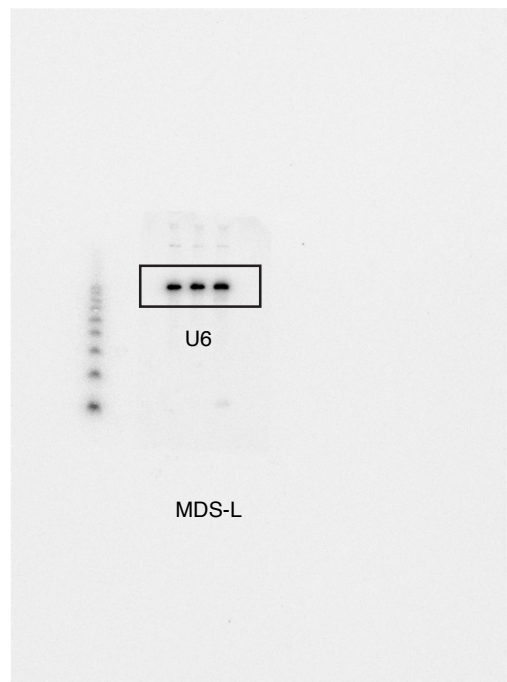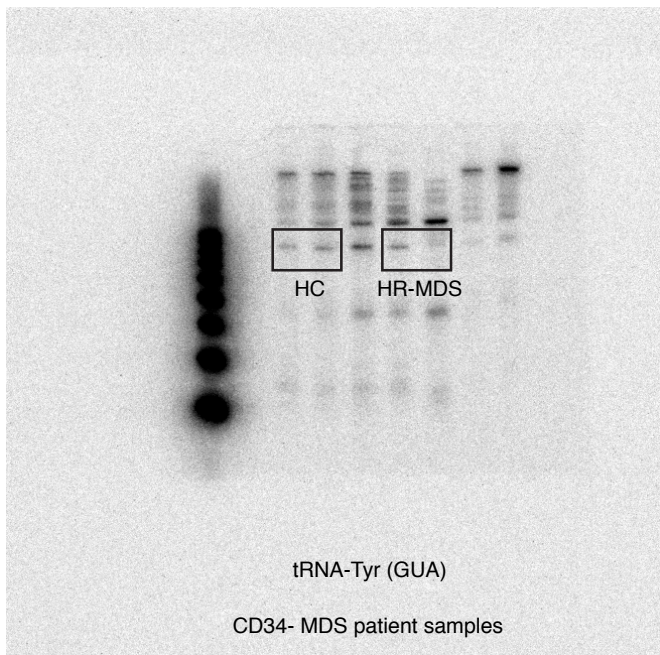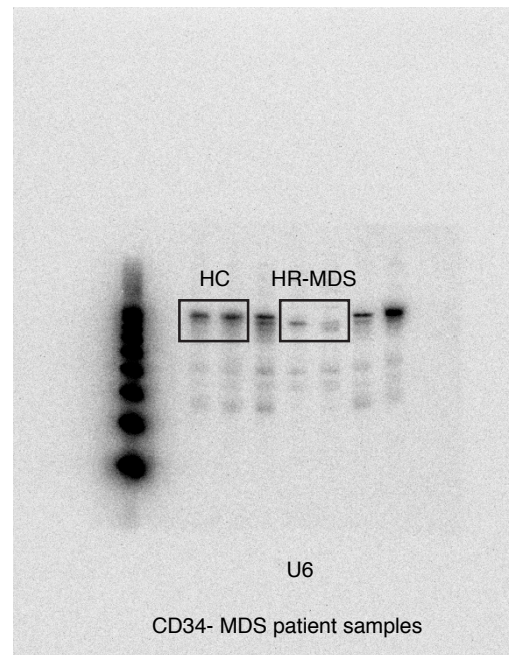

Supplement: Source Data Extended Data Fig. 7 — Unprocessed western blots and/or gels. [file 41556_2022_852_MOESM20_ESM.pdf]

Related to Extended Figure 8b

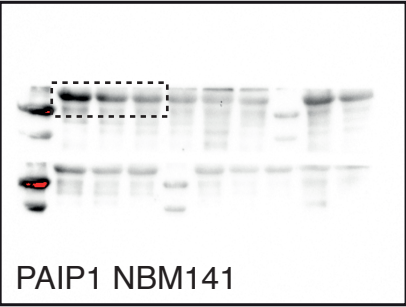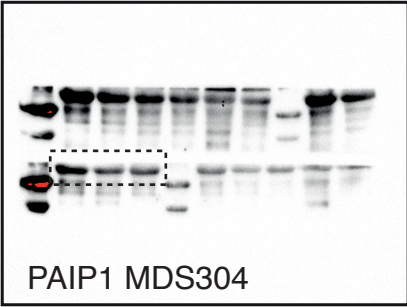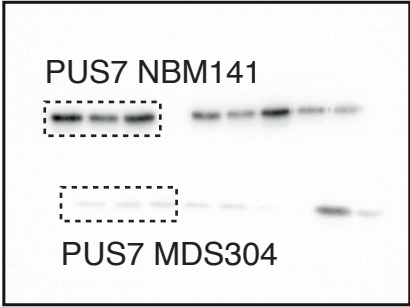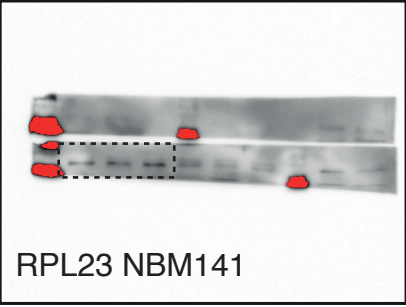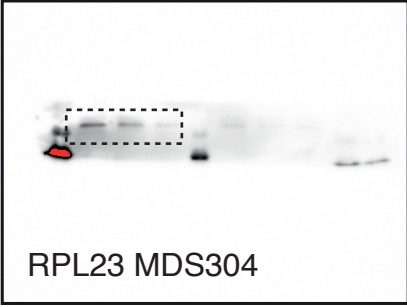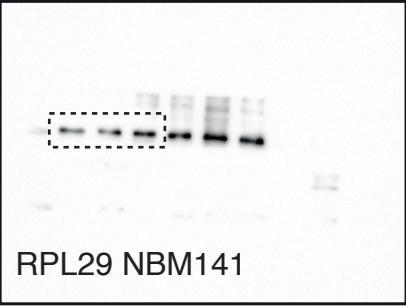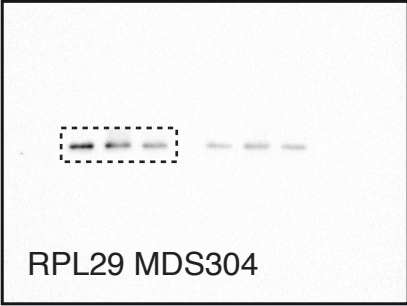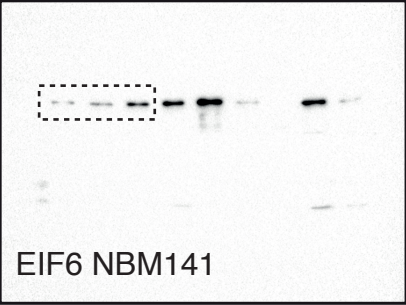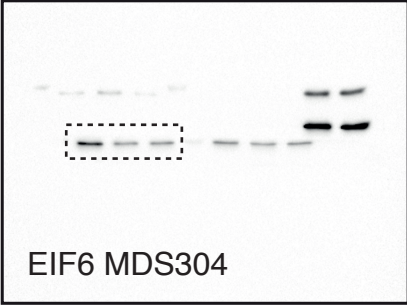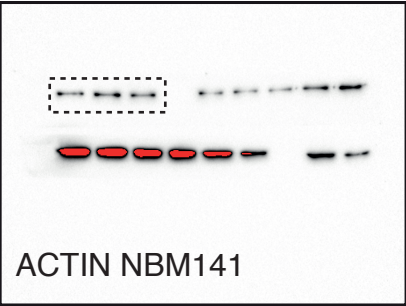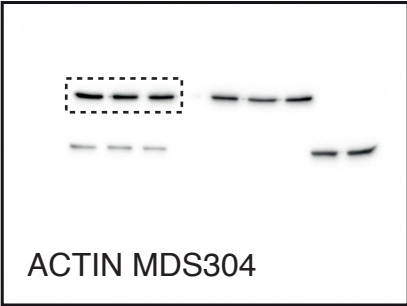

Supplement: Source Data Extended Data Fig. 8 — Unprocessed western blots and/or gels. [file 41556_2022_852_MOESM22_ESM.pdf]
