# Supplementary material for: Pseudouridine-modified tRNA fragments repress aberrant protein synthesis and predict leukaemic progression in myelodysplastic syndrome
Source: Nat Cell Biol. 2022 Mar 15;24(3):299–306. doi: 10.1038/s41556-022-00852-9 (PMC8924001; doi:10.1038/s41556-022-00852-9)

Related to Figure 3

Related to Figure 3d

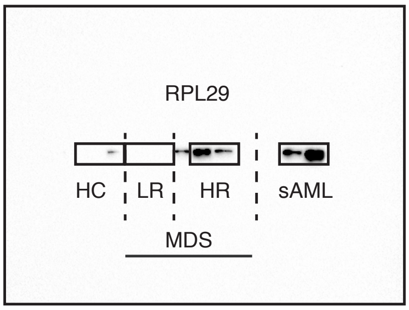

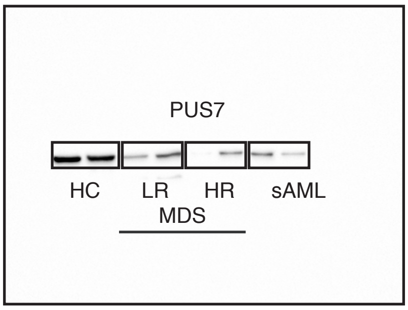

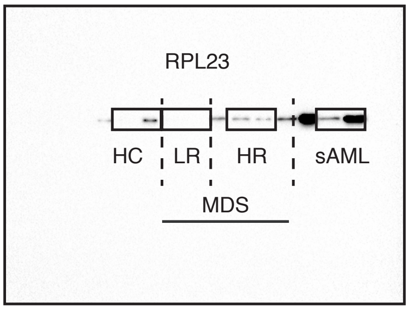

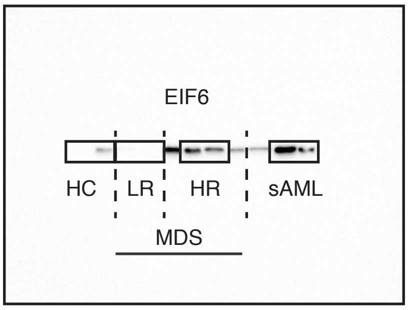

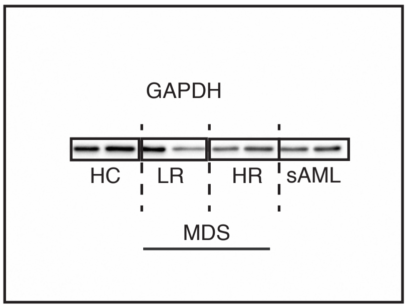

Related to Figure 3e

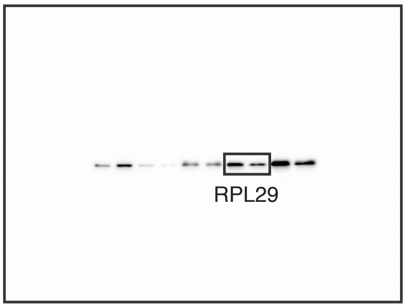

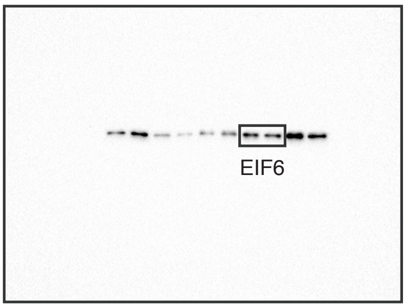

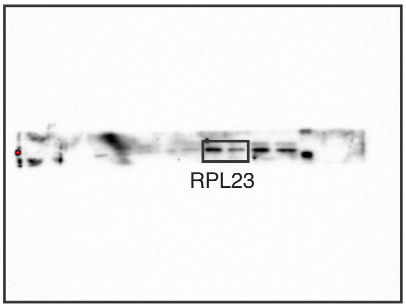

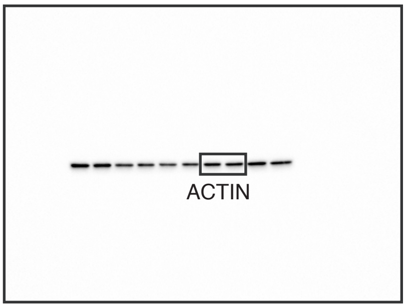

Related to Figure 3

Related to Figure 3f

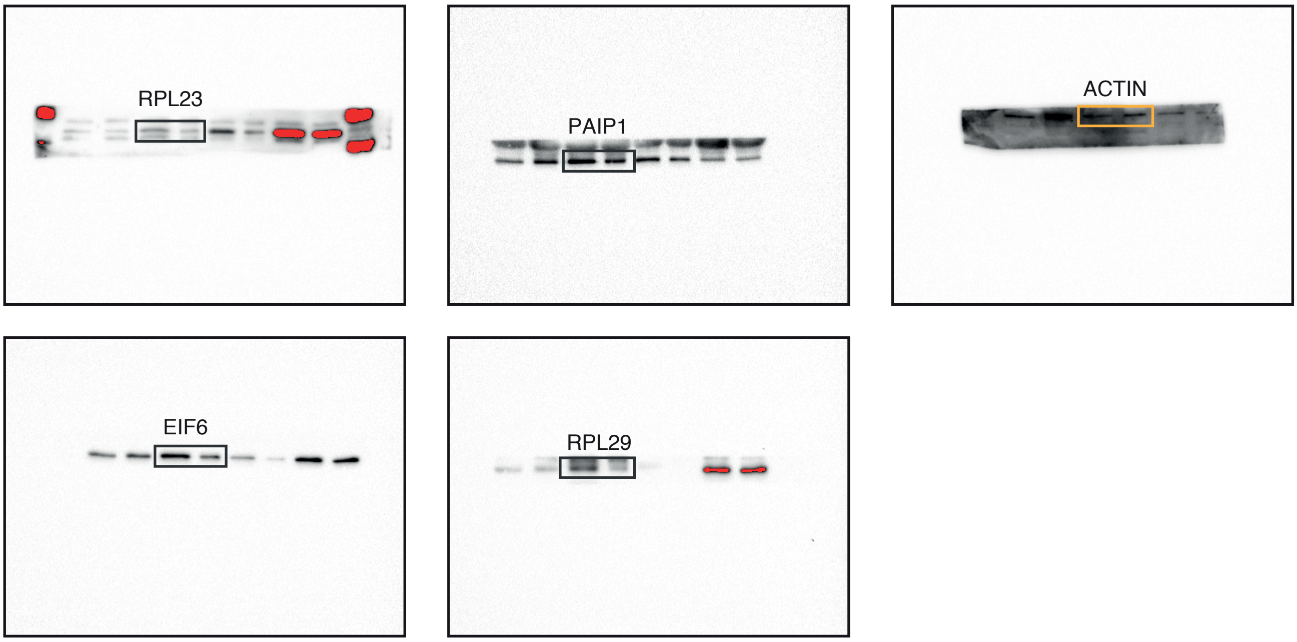

Related to Figure 3g

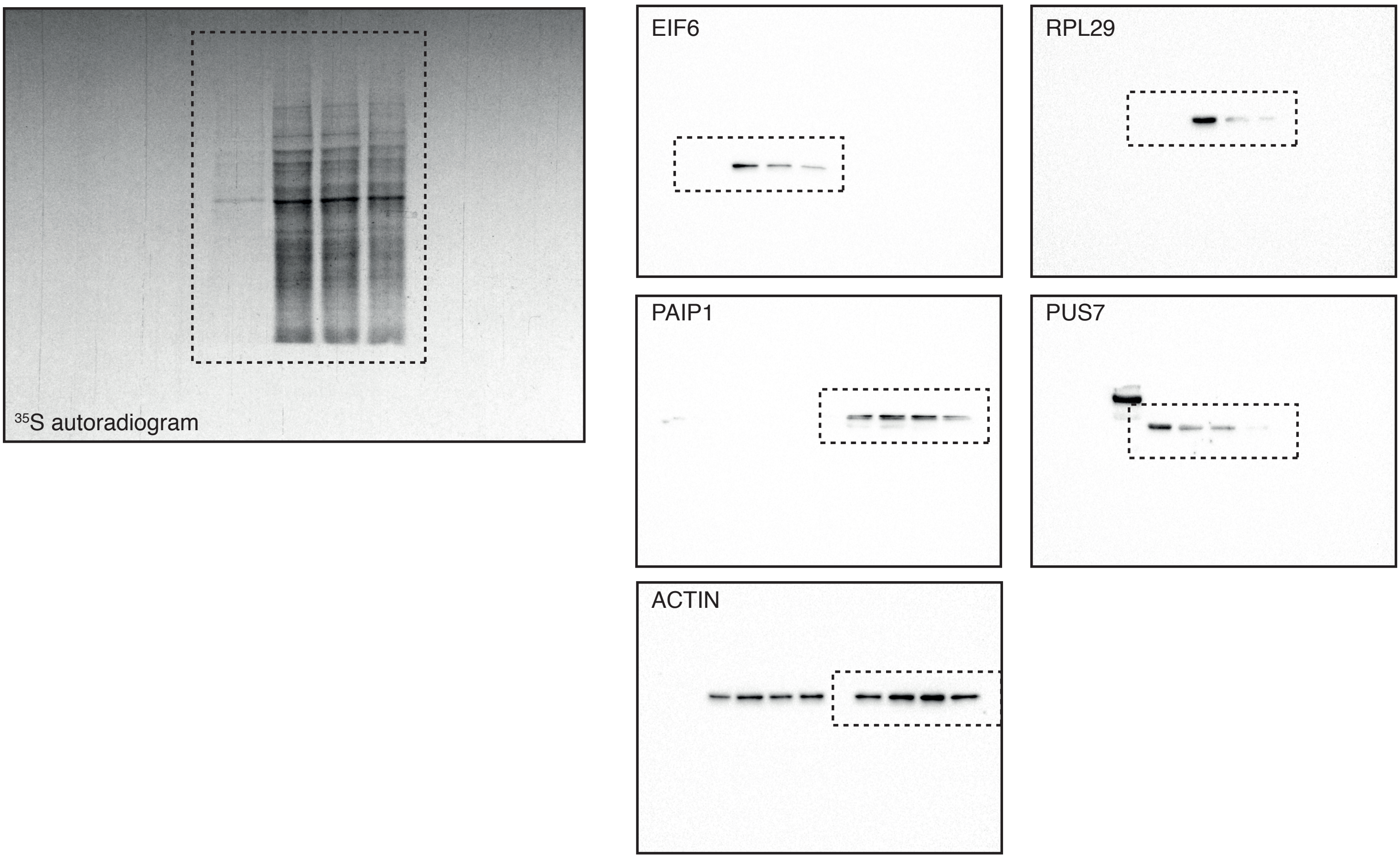

Supplement: Source Data Fig. 3 — Unprocessed western blots and/or gels. [file 41556_2022_852_MOESM9_ESM.pdf]
