# Supplementary material for: Pseudouridine-modified tRNA fragments repress aberrant protein synthesis and predict leukaemic progression in myelodysplastic syndrome
Source: Nat Cell Biol. 2022 Mar 15;24(3):299–306. doi: 10.1038/s41556-022-00852-9 (PMC8924001; doi:10.1038/s41556-022-00852-9)
Supplement: Supplementary file 3 — Peer Review Information [file 41556_2022_852_MOESM3_ESM.pdf]

natureresearch

Peer Review Information

Journal: Nature Cell Biology
Manuscript Title: Pseudouridine-modified tRNA fragments repress aberrant protein synthesis and predict leukemic progression in myelodysplastic syndrome
Corresponding author name(s): Marios Dimitriou, Cristian Bellodi

Reviewer Comments & Decisions:

Decision Letter, initial version:

Subject: Decision on Nature Cell Biology submission NCB-B44478
Message:

Dear Dr Bellodi,

Please accept our sincere apologies for the length of time your manuscript has been under consideration at our journal, which was due to the difficulties in securing a full panel of referees during the winter holidays. Thank you for your patience during this time.

Your manuscript "Pseudouridine-modified tRNA fragments repress malignant protein synthesis and predict leukemic progression in myelodysplastic syndrome", has now been seen by 3 referees, who are experts in small RNAs and RNA modifications (referee 1); translation and lymphoma (referee 2); and myelodysplastic syndrome (referee 3), and whose comments are pasted below. In light of their advice, we regret that we cannot offer to publish the study in Nature Cell Biology.

As you will see, although the reviewers find this work interesting, they raise serious concerns that question the strength of the data and of the novel conclusions that can be drawn at this stage.

We are very sorry that we could not be more positive on this occasion, but we thank you for the opportunity to consider this work.

With kind regards,
Jie Wang

naturereseach

Jie Wang, PhD
Senior Editor
Nature Cell Biology

Tel: +44 (0) 207 843 4924
email: jie.wang@nature.com

Reviewers' comments:

Reviewer #1 (Remarks to the Author):

A. Summary
This study describes a previously unknown mechanism of translational control mediated by mTOG, a family of tRNA fragments. Here, the authors seek to mechanistically dissect this pathway and interrogate its possible role in tumorigenesis.

B. Originality and significance
This work builds on an earlier study by the authors. This on its own is not a major concern as the pathway described is distinct. However, I don't think the findings are in their current state rigorous enough to be of broad significance.

C. Data & methodology
My main concern with this study is that in its current form, it has made up its mind from the very beginning about the story that it wants to tell. Therefore, numerous alternative hypotheses have not been considered, and the pathway structure is not always rigorously established. More specific concerns are discussed below.

D. Appropriate use of statistics
There are some concerns about the visualization and analyses of the presented data. Below, I have listed some of these:
Fig 1C: From the figure, it seems that the number of replicates is 2, SD or SEM is meaningless for 2 data points.
Fig 1D: Not convincing. Effect size small and U-test (which is the best method for comparing 4 vs 4) would not be significant.
Fig 2B: Consideration of statistical significance is missing. Repeat the analysis with a specialized comparative tool (e.g. xtail or babel) and show volcano plots. Especially for the 3 selected genes.
Fig 2f: analysis is not done correctly. Normalizing reps to 1 has resulted in WT values to be 1.0 with SD of 0. Artificially low SD in the WT cohort impacts ANOVA p-values.

In general, dynamite plots should be avoided.

This list is by no means comprehensive. I suggest every figure be examined for correct application of statistical analyses.

E. Conclusions
Overall, the conclusions are supported by the observations. However, my biggest concern with this study is that in many places throughout the paper protein levels and translation efficiency are used interchangeably. Almost every experiment presented requires inclusion of mRNA measurements to exclude transcript levels as a co-variate, which is missing. Absent this information, it is difficult to tease apart indirect from direct impact of translation.

F. Suggested improvements:
In addition to the above (i.e. inclusion mRNA measurements; e.g.
in fig 2e and g: repeat with qPCR for the mRNAs to show that the effects are due to translational changes), another major concern is that the study is quite disjointed. There is molecular part and a cancer part. The two don't communicate. For example, the components of the pathway described in the first sections are not then rigorously studied for their requirement and sufficiency in the cancer side. I would propose the following experiments:

Fig 3a: The levels of tRNAs should be included as a co-variate (here and throughout the study); also included should be the genes discussed here (e.g. PAIP1); there should a significant interaction term in a multivariate cox-ph analysis.

Fig 3d: Show RNA results; show results are PAIP1 dependent.

Fig 4: The most important piece of data missing here is epistasis . This is needed to show that the mTOG's effect is mediated through the identified pathway.

Does exogenous mTOG has an impact of tRNA content of the cell?

Reviewer #2 (Remarks to the Author):

A rather innovative topic and also a follow up on the authors' recent Cell paper that reports a role of mTOG, a small tRNA fragment with potential roles in stem cell biology that are mediated by mTOG binding to PABPC1.

The authors claim the following main points:

1. A selective effect of mTOG on oncogenic translation programs.
However, their ribosme profiling study indicates a broad, not a selective effect on translation.

2. Effect is mediated by interaction of mTOG with PABPC1 that impair binding of PAIP1 and inhibition of the eIF4F complex.

EMSA and a MS technique and pulldowns are used to show confirm and map the first interaction. It is not clear what other proteins mTOG would bind, and therefore what is the specific role of PABPC1 and the specific binding site. The effect on PAIP1 is compared to PAIP2 (not affected) establishing a level of selectivity. The authors also use PUS7 k/o cells. It is not clear to what extent PUS7 ko cells have a specific defect in mTOG versus more broad changes in . Together, the authors show binding and a potential mechanism, they do not address the sufficiency/requirement and potential role of other binding partners.

3. Loss of this interaction increases translation of mRNAs with specific features referred to as PES and that include TOP sequence elements.

The authors report a ribosome profiling study on PUS k/o cells. They report a "global increase" and also refer to >2000 differentially translated mRNAs with a higher fold-change than others. The authors do not discuss the effect of Pus7 loss on gene expression, the statists of this analysis is questionable with a q value of 0.25. Theactual q/FDR values are not provided. The inset in 2B appears to show a general shift in translation and not a specific effect. Among mRNAs that show increased translation the authors identify an enrichment of GC rich mRNAs with short UTRs and TOP and TOP like mRNAs. It is not examined which RNAs are relatively less translated.

4. PES and TOP mRNAs include many ribosomal proteins and other translation factors and have been linked to mTOR signaling and a global increase in translation capacity.

This is consistent with a global effect on translation and contradict a selective effect on an oncogenic translation program claimed above. as this program is not thought to be cancer specific, but rather a broad effect on all/most mRNAs. The authors highlight the role of ribosomal protein RPL23 that had been linked to delayed leukemia development. Genetic epistasis studies confirm effects on various

ribosomal proteins. These data do not, in my mind, support the proposed malignant translation program but are more consistent with a global effect on translation.

5. A role for mTOG ⍰ in MDS-derived HSCs and their malignant progression.
The authors indicate mTOG ⍰ represses protein production without effect on viability or proliferation, which seems hard to believe. Effects on translation on specific proteins seem variable and actin is among the most altered proteins. Colony assays indicate mTOG ⍰ enhances colonies. Engraftment in animals are less convincing than described as 2/5 mTOG ⍰ animals seem to have rather low engraftment and ¼ controls has good engraftment. This is not convincing.

6. A clinical association between mTOG ⍰ and disease severity/progression in MDS/AML.

The link to monosomy7 is interesting. However, another gene (SAMD9) has been clearly implicated in mono7 MDS. https://www.ncbi.nlm.nih.gov/pmc/articles/PMC5540771/
PUS7 is indeed on Chr. 7 and has been linked to a developmental disorder. Is there direct genetic evidence that this pathway and its key enzymes are involved in leukemia? The authors cite a general review and their own work. However, their cell paper only shows reduced PUS7 in monosomy7 cells not a role in disease. The authors report an association with severity in a retrospective and targeted analysis. This is consistent with but does not prove a functional role as claimed and likely many other genes are equally associated.

Reviewer #3 (Remarks to the Author):

The authors present compelling data that stem-cell enriched tRNA derived fragments (mTOG) and their role in regulating protein translation. This submission builds upon the group's previous findings on pseudouridylation of mTOGs in embryonic stem cells (Guzzi, Cell 2018). They showed that the pseudouridylation "writer" PUS7 modifies and activates a novel network of mTOGs targeting the translation initiation complex. PUS7 inactivation in embryonic stem cells impairs tRF-mediated translation regulation, leading to increased protein biosynthesis and defective germ layer specification. In this submission, they extended these mechanistic studies to pre-leukemic hematopoietic cells. Herein, they showed that mTOG targets PABC1, which hinders the recruitment of PAIP1 and translation of transcripts sharing 5-UTR pyrimidine-enriched sequences. Lastly, they show that mTOG dysreulgation is implicated in MDS HSPCs and may affect leukemic transformation. Overall, the new mechanistic studies describing a link between mTOG, PABPC1, and PAIP1 are convincing and supported by rigorous studies (Figure 1-2). However, the potential role of mTOG dysregulation in the pathogenesis of MDS is weak and not supported by the current data.

- It is unclear how transient transfection of the pseudouridylated mTOGs achieves durable suppression of translation in primary MDS cells over several days (Fig 3-4)? What is the transfection efficiency? How long do the MDS cells maintain expression of the oligos?
- The concept that MDS erythroid cells exhibit increased translation is not necessarily consistent with prior literature. It is counter intuitive that decreasing protein translation will result in increased MDS erythroid differentiation (Fig 4). In fact, many subtypes of MDS exhibit aberrant ribosome protein expression and as a result reduced translation rates.
- The immunoblots in Figure 3d should be on a single gel in order to draw conclusions on subtle changes in protein expression between the groups.
- There are significant concerns with the MDS xenograft model. Why is there no engraftment of HR-MDS samples when treated with SCR oligo (Fig 4g)? Given that the engraftment is effectively at zero, it is also impossible to interpret the data in panel 4i. It is also counterintuitive as to why there is increased engraftment of HR-MDS cells treated with mTOG oligos. Based the proposed importance of mTOG-PABC1-PAIP1 in MDS, I would expect that expression of mTOG oligos would eliminate/decrease the HR-MDS clone, as would be the goal therapeutically, not to increase its engraftment potential.

**Although we cannot offer to publish your paper in Nature Cell Biology, the work may be appropriate for another journal in the Nature Research portfolio. If you wish to explore suitable journals and transfer your manuscript to a journal of your choice, please use our manuscript transfer portal. If you transfer to Nature-branded journals or to the Communications journals, you will not have to re-supply manuscript metadata and files. This link can only be used once and remains active until used.
All Nature Research journals are editorially independent, and the decision to consider your manuscript will be taken by their own editorial staff. For more information, please see our manuscript transfer FAQ page. Note that any decision to opt in to In Review at the original journal is not sent to the receiving journal on transfer. You can opt in to In Review at receiving journals that support this service by choosing to modify your manuscript on transfer. In Review is available for primary research manuscript types only.

**For Nature Research general information and news for authors, see http://npg.nature.com/authors.

Author Rebuttal to Initial comments

Response to the reviewers NCB-B44478

Response to reviewer #1

We thank the reviewer for recognizing the novelty and significance of our study, and for suggesting additional experiments, which greatly helped us improve our manuscript. As suggested, we rigorously review all the statistical analysis, included complete mRNA quantifications, and measured tRNA levels upon mTOG treatment. Most importantly, we have performed additional experiments that broaden the significance of our findings by strengthening the links between the molecular and clinical parts of the paper, which unambiguously demonstrate the importance of the PUS7/mTOG/PAIP1 pathway for the regulation of central metabolic and phenotypic features of malignant MDS-HSPCs.

Response to the specific comments.

1. The reviewer states that "*There are some concerns about the visualization and analyses of the presented data*". Specifically, he/she suggests that "*every figure be examined for correct application of statistical analyses*".

As requested by the reviewer, we revised and updated data presentation and analysis for several panels including Figs 1c-d, 2b, 2e-g, 3d-e, 4b, 4e, 4h-i and Extended data figs. 1a, 2a-d, 3c, and 8a. Specifically, we have addressed the individual points below:

1.1 *Fig 1c: From the figure, it seems that the number of replicates is 2, SD or SEM is meaningless for 2 data points*.

In Figure 1c we have now included three replicates and applied a t-test with Welch correction, which provides a statistically significant p-value (0.0415) between control RNA and mTOG-∴ϑ. Conversely, no statistical significance is observed when control RNA and SCR-∴ϑ are compared.

1.2 *Fig 1D: Not convincing. Effect size small and U-test (which is the best method for comparing 4 vs 4) would not be significant*.

As requested, we applied the Mann-Whitney U test for the WT vs PUS7-KO comparison, which shows significant difference with p=0.0286 as stated in the text (page 4), whereas no statistically significant differences are observed when comparing WT and PUS7-KO cells treated with mTOG-∴ϑ (p>0.99). We updated the figure accordingly.

1.3 *Fig 2B: Consideration of statistical significance is missing. Repeat the analysis with a specialized comparative tool (e.g. xtail or babel) and show volcano plots. Especially for the 3selected genes*.

We included the consideration of statistical significance related to ribosome profiling on page 4. Furthermore, we would like to clarify that the rationale for selecting candidate genes was ultimately based on the remarkable enrichment for functional categories such as "translation" and "translation initiation" observed in PUS7-KO cells as well as on the relevance for MDS pathogenesis. As suggested by the reviewer, we tried Xtail and Babel on our datasets; however, we did not obtain meaningful results using these pipelines. We speculate that thesecomparative tools are not particularly suitable for analysis of less than 3 replicates due to a low statistical power and, in situation of unidirectional global changes in translation (increaseor decrease) as discussed by McGlincy and Ingolia Methods 2017.

1.4 *Fig 2f: analysis is not done correctly. Normalizing reps to 1 has resulted in WT values to be 1.0 with SD of 0. Artificially low SD in the WT cohort impacts ANOVA p-values. In general,dynamite plots should be avoided*.

Following the reviewer's suggestion, we updated Fig. 2f with *de novo* protein synthesis shownas the $\log_2$ FC PUS7-KO/WT, which eliminates the normalization bias introduced by having the WT replicates set to 1 and a low SD. To provide complete information on the results, we included the individual data points in this and all the other bar plots throughout the main and supplementary figures.

2. The reviewer points that "*my biggest concern with this study is that in many places throughout the paper protein levels and translation efficiency are used interchangeably*". Furthermore, he/she adds "*every experiment presented requires inclusion of mRNA measurements to exclude transcript levels as a co-variate*".

As suggested by the reviewer, we have included the mRNA measurements along with the protein analysis in Figs. 2e, 2g, 3d,g, which demonstrates the specific translation regulation for each individual candidate gene regulated downstream of the PUS7/mTOGs/PAIP1 axis in hESC, human MDS-L cells and primary patient-derived MDS bone marrow cells (Fig. 3d-h). As stated in the manuscript (page 4), genes with transcription changes (|log2 FC mRNA KO/WT| > 1) were excluded from the analysis of translationally regulated genes in PUS7-KOcells (Fig. 2b). Moreover, we have now included additional experiments illustrating that translation control by mTOG-∴ϑ involves the 5' PES motif embedded with the 5' UTRs of RPL23, RPL29 and EIF6 transcripts identified using ribosome profiling (Figs 2h-j and Extended Data Fig. 3d).

3. The reviewer points that "*another major concern is that the study is quite disjointed. Thereis molecular part and a cancer part. The two don't communicate*". He/she adds:" *the components of the pathway described in the first sections are not then rigorously studied for their requirement and sufficiency in the cancer side*".

Following the reviewer suggestion, we have extended our analysis of PUS7, mTOG and PAIP1 to extensively validate the molecular and functional epistasis between the pathway components in patient-derived primary MDS stem cells and in the human MDS-derived cell line, MDS-L

(Tohyama et al., Br J Haematol. 1995) (Fig. 3 e-h and 4b-e). These new results (highlighted on page 7) tightly link the molecular components identified in the first section to the clinical findings presented in Figs 3 and 4.

3.2 The reviewer raised the issue that "*Fig 3a: The levels of tRNAs should be included as a co-variate (here and throughout the study*).

The analysis in Fig. 3a was performed using limited amount of total RNA obtained from small population of primary MDS hematopoietic stem and progenitor cells. As such, we are unable to comprehensively investigate tRNA levels in this patient cohort. However, we have included northern blot analysis for tRNA-Tyr (GUA), a well-established PUS7 target (Behn-Ansmant et al., RNA 2003, Urban et al., J Biol Chem 2009), in PUS7-KO hESCs and MDS-L cells in the absence or presence of mTOGs. Consistent with our previous tRNA quantification (Guzzi et al., Cell 2018) and codon usage analysis (Extended Data Fig. 2d) illustrating no significant differences in *PUS7-KO* cells, we found that PUS7 loss and/or mTOG transfection did not alter the levels of tRNA-Tyr (GUA) in any of the cell system tested (Extended Data Figs. 2h, 7a). Of note, similar results were obtained comparing primary $CD34^-$ bone marrow mononuclear cells (BMMNC) from two distinct HR-MDS patients with low PUS7 and mTOG levels, to two age-matched healthy donors (Extended Data Fig. 7a).

3.3 He adds "*also included should be the genes discussed here (e.g. PAIP1); there should a significant interaction term in a multivariate cox-ph analysis*".

As requested, we analyzed PAIP1 and PABPC1 mRNA levels by ddqPCR within the same MDS patient cohort (n=50) used for PUS7 and mTOG quantification and applied the multivariate Cox model controlled for age (Supplementary Table 6). This analysis shows thatPUS7, mTOG and PABPC1 have independent and additive effects on survival. Notably, PABPC1 was not correlated with mTOG, whereas PAIP1 positively correlated with mTOG (R=0.35, p=0.013) and PABPC1 (R=0.36, p=0.011). However, PAIP1 did not reach statistical significance in the Univariate Cox´s model (p=0.155). Therefore, it was excluded from the multivariate analysis. Together these findings suggest that dysregulation of the pathway either at the level of PUS7/mTOG or through perturbations of other components such as PABPC1, leads to the same prognostic effects in MDS.

3.4 The reviewer requests that "*Fig 3d: Show RNA results; show results are PAIP1 dependent*".

We employed siRNA pools to deplete PAIP1 and selectively rescue increased bulk protein synthesis and translation of PES-containing mRNAs in primary patient derived MDS cells as well as in PUS7-depleted MDS-L cells (Fig. 3f-h). Consistent with our findings in hESCs, we show that mTOG translation-repressive function is exquisitely dependent on PAIP1 in these cells.

3.5 The reviewer comments "*Fig 4: The most important piece of data missing here is epistasis.This is needed to show that the mTOG's effect is mediated through the identified pathway*".

As suggested, we performed additional experiments illustrating that PAIP1 depletion significantly improves differentiation of HR-MDS stem cells in CFU assays and phenocopy the effects of mTOG transduction in these cells (Fig. 4b). Of note, we observed similar effects in response to mTOG and siPAIP1 transfection in MDS-L cells (Fig. 4c), which further highlightsan epistatic interaction between these pathway components in MDS.

3.6 The reviewer asks: “*Does exogenous mTOG has an impact of tRNA content of the cell?*”.

See point above 3.2, we assessed the effects of mTOG transfection on tRNA-Tyr (GUA) levels and found no differences in either hESCs or MDS-L cells (Extended Data Figs. 2h and 7a).

Response to Reviewer #2

We are pleased that the reviewer finds that our work represents “*A rather innovative topic*” and highlights that our new story “*reports a role of mTOG, a small tRNA fragment with potentialroles in stem cell biology that are mediated by mTOG $\lambda\vartheta'$ binding to PABPC1*”. We thank thereviewer for his/her comments, which helped us strengthen the manuscript. In response to thereviewer’s concerns, we have address each of the issues raised.

Response to the specific comments.

1. The reviewer states: “*A selective effect of mTOG $\lambda\vartheta'$ on oncogenic translation programs*” andadds “*however, their ribosome profiling study indicates a broad, not a selective effect on translation*”.

The ribosome profiling analysis is consistent with our previous finding that PUS7 and mTOG impairments are associated with perturbation of bulk protein synthesis (Guzzi et al., Cell 2018). Furthermore, we provide compelling evidence that mTOG-$\lambda\vartheta'$ directly fine tunes translation of functionally distinct mRNA subsets enriched for ribosomal proteins and translation factors (Fig. 2c). Critically, our new results indicate that this effect is at least in part mediated through the regulation of the PES motif embedded in the 5’ UTR of these mRNAs (Fig. 2d, 2h-j, Extended data Fig. 3d), which may provide an additional layer of regulation to control ribosomebiogenesis and translation. Since dysregulation of ribosome biogenesis and translation is associated with malignant transformation downstream of prominent oncogenic signaling pathways including PI3K/AKT/mTOR, MYC and RAS/MAPK (Gentilella et al., Biochim Biophys Acta. 2015; van Riggelen et al., Nat Rev Cancer 2010; Shaw et al., Nature 2006; Truitt et al.,Nat Rev Cancer 2016), our findings highlight a key role for dysregulation of PUS7/mTOG in driving aberrant protein synthesis in distinct stem and cancer cell populations.

2.1 The reviewer comments “*Effect is mediated by interaction of mTOG $\lambda\lambda'$ with PABPC1 thatimpair binding of PAIP1 and inhibition of the eIF4F complex*”. Furthermore, he/she points that:”*It is not clear*

what other proteins mTOG λλ′ would bind, and therefore what is the specific roleof PABPC1 and the specific binding site. The effect on PAIP1 is compared to PAIP2 (not affected) establishing a level of selectivity".

We previously showed that several proteins may bind mTOG-λλ′ (Guzzi et al., Cell 2018); however, our *in vitro* and *in vivo* analysis compellingly demonstrate that the molecular interactions between mTOG, PABPC1 and PAIP1 (Fig. 1) are sufficient and critically requiredto enable control of bulk and mRNA-specific translation downstream of PUS7 in hESCs, MDS-L cells and HSPCs (Fig. 2e,g, 3c, 3e-g, Extended Data Fig. 8b). Additionally, we provide new evidence that depletion of PAIP1 not only restores translation but also improves differentiationof PUS7-depleted MDS-L cells and HR-MDS HSPCs, thus functionally phenocopying mTOG-λλ′ transduction in these cells (Figs. 4b,c). Taken together, these results provide a mechanistic explanation to our previous findings that mTOG-PABPC1 interaction is specific and directly dictates protein synthesis and stem cell lineage commitment downstream of PUS7 in embryonic and hematopoietic stem cells.

2.2 The reviewer points "*The authors also use PUS7 k/o cells. It is not clear to what extent PUS7 ko cells have a specific defect in mTOG λλ′ versus more broad changes in λλ*".

Although it is possible that PUS7 loss may affect λλ′ modifications of other RNAs including the anticodon position U35 in tRNA-Tyr (GUA) (Urban et al., J Biol Chem 2009; Behm-Ansmant et al., RNA 2003), our results that PUS7 selectively targets λλ′ to control mTOGs activity in hESCs and HSPCs, provide a unique biologically relevant context for the function of the PUS7/mTOG axis in translation control. Importantly, we demonstrate that reintroduction of mTOG-λλ′ fully restores protein synthesis and stem cell differentiation in PUS7-KO hESCs andHSPC (Guzzi et al., Cell 2018). Importantly, we provide evidence that similar mTOG effects were selectively observed in primary HR-MDS HSPCs and upon PUS7 depletion in the MDS-derived cell line, MDS-L, characterized by aberrantly high levels of translation (Figs. 3c, 3g,hand 4b-e).

2.3 The reviewer raised the issue that "*Together, the authors show binding and a potential mechanism, they do not address the sufficiency/requirement and potential role of other bindingpartners*".

Our molecular analysis illustrates that mTOG-λλ′ binding to PABPC1 RRM2-3 and regulationof PAIP1 as a primary mechanism for translation control in hESCs and HSPCs. Our results that mTOG-λλ′ transduction, depletion of PABPC1 (Guzzi et al., Cell 2018 and this manuscript Extended Data Fig. 3a) or PAIP1 (Figs. 2f,g and 3f,g) selectively restores protein synthesis in PUS7-KO cells, provide strong evidence that the epistatic interaction between these components are functionally sufficient to define a distinct molecular pathway governing translation initiation in stem cells.

3.1 The reviewer states "*The authors report a ribosome profiling study on PUS k/o cells. Theyreport a "global increase" and, also refer to >2000 differentially translated mRNAs with a higherfold-change than others. The authors do not discuss the effect of Pus7 loss on geneexpression, the statistic of this analysis is questionable with a q value of 0.25. The actual q/FDR values are not provided*".

Our analysis of translation was based on the criterion that no significant changes in mRNA expression (-1< log_2 FC mRNA KO/WT <1) were detected for the individual genes, we specify this in the text (page 4) and in the methods. Our results are consistent with our previous studies that PUS7 depletion primarily alters translation in the absence of transcriptional effects (Guzziet al., Cell 2018). Importantly, we have improved our Ribo-seq analysis mapping the reads onthe more recent GENCODE 35 release and applying more stringent cut-offs for translation efficiency (Log_2 FC TE KO/WT > 1) and FDR < 0.2 (Fig. 2b,c). As requested, we have included the FDR values for the ribosome profiling study in Supplementary Table S2.

3.2 He/she adds "*The inset in 2B appears to show a general shift in translation and not a specific effect. Among mRNAs that show increased translation the authors identify an enrichment of GC rich mRNAs with short UTRs and TOP and TOP like mRNAs*" and asks "*It is not examined which RNAs are relatively less translated*".

We examined 949 transcripts relatively less translated with log_2 FC TE < 0.6415 correspondingto the bottom 15% TE FC and FDR < 0.2 in PUS7-KO cells, which classified within functional categories related to transcription and splicing. Notably, we could not find enrichment for ribosome/translation-related functional categories nor 5' PES-containing mRNAs (p>0.05) among these relatively less translated transcript fractions in PUS7-KO cells.

4.1 The reviewer remarks that "*PES and TOP mRNAs include many ribosomal proteins and other translation factors and have been linked to mTOR signaling and a global increase in translation capacity*". Furthermore, he/she points that "*This is consistent with a global effect on translation and contradict a selective effect on an oncogenic translation program claimed above. as this program is not thought to be cancer specific, but rather a broad effect on all/mostmRNAs*".

We agree that several 5' PES/TOP containing mRNAs identified in our ribosome profiling analysis have been linked to mTOR signaling and regulation of global protein synthesis. Indeed, we previously showed that PUS7 and mTOG impairments were associated with perturbation of bulk translation (Guzzi et al., Cell 2018). Building on these original observations, our ribosome profiling analysis reveals remarkable changes at the translation level of individual 5' PES-containing transcripts (Figs. 2e, 2g-j and Extended Data Fig. 3d). These data are in line with recent evidence highlighting that regulation of these 5'TOP/TOP- like mRNAs downstream of mTORC1 is complex and likely involved multiple cell-type specificmechanisms (Philippe et al., PNAS; Cockman et al., Biomolecules 2020). Importantly, we findthat 5' PES-containing mRNAs including RPL23, RPL29, eIF6 are exquisitely dependent on mTOG-4′′ and PAIP1 in PUS7-depleted hESCs, MDS-L and HR-MDS HSPCs (Fig. 2e-j, 3e-h,Extended Data Figs. 3d and 8b). Furthermore, this defines a unique dependency for translation programs that selectively steer the metabolism of distinct stem and cancer cell populations. Lastly, our results may provide a mechanistic explanation to findings that several 5' TOP mRNAs are weakly sensitive to mTORC1 activity and LARP1-mediated regulation (Philippe et al., PNAS 2020).

4.2 The reviewer adds "*The authors highlight the role of ribosomal protein RPL23 that had been linked*

to delayed leukemia development. Genetic epistasis studies confirm effects on various ribosomal proteins. These data do not, in my mind, support the proposed malignant translation program but are more consistent with a global effect on translation".

As stated above (see reviewer #2 point 1), the defects in 5'PES mRNA translation reported in our study highlight ribosome biogenesis and protein biosynthesis as major cancer-promoting programs directly altered downstream of PUS7/mTOG in HR-MDS.

5.1 A role for mTOG 4′′ in MDS-derived HSCs and their malignant progression. The reviewerpoints "*The authors indicate mTOG 4′′ represses protein production without effect on viabilityor proliferation, which seems hard to believe*".

We would like to emphasize that mTOG-4′′ transduction (or PAIP1 depletion) does not block protein synthesis but rather restores translation in HR-MDS HSPCs to levels observed in normal hematopoietic and LR-MDS HSPC and without effects on viability and proliferation (Figs. 3c and Extended data Fig. 6b-e). This is consistent with our previous findings in hESCsand HSPCs that mTOG has no effects on viability and proliferation (Guzzi et al., Cell 2018), and with results that differences in protein synthesis are uncoupled from changes in HSPCs cell division (Signer et al., Genes & Development 2016).

5.2 He/she remarks that" Effects *on translation on specific proteins seem variable and actin isamong the most altered proteins*".

Following the reviewer suggestion, we have included another housekeeping gene, GAPDH, as a control for the protein analysis of PUS7/mTOG-regulated mRNAs in primary MDS patient samples (Fig. 3d).

5.3 The reviewer comments "*Colony assays indicate mTOG 4′′ enhances colonies. Engraftment in animals are less convincing than described as 2/5 mTOG 4′′ animals seem to have rather low engraftment and ¼ controls has good engraftment*".

In response to this point and comments from referee #3, we have expanded our analysis *in vivo* including xenotransplantation of $CD34^{+}$ cells derived from two additional HR-MDS and one extra LR-MDS patients (Fig. 4f-j and Extended data Fig. 10a,b). Our new results illustrate engraftment levels comparable to those achieved in previous studies (Medeyouf et al., Cell Stem Cell 2014). Notably, our findings are consistent with our original observations that mTOG selectively enhanced engraftment (>2 fold) and "non-leukemic" multilineage differentiation of HR MDS-HSPC but not in LR-MDS (Fig. 4g-i and Extended data Fig 10a,b). These effects coupled with significant reductions in the frequency of $CD123^{+}$ leukemic stem cells (Fig. 4j), compellingly support our findings that mTOG target malignant stem cell populations characterized by abnormally high protein synthesis levels (Stevens et al., Nat Comm 2018).

6. The referee remarks that" *The link to monosomy7 is interesting. However, another gene (SAMD9) has been clearly implicated in mono7*

MDS.https://www.ncbi.nlm.nih.gov/pmc/articles/PMC5540771/ PUS7 is indeed on Chr. 7 andhas been linked to a developmental disorder". He/she adds "*Is there direct genetic evidence that this pathway and its key enzymes are involved in leukemia? The authors cite a general review and their own work. However, their cell paper only shows reduced PUS7 in monosomy7cells not a role in disease. The authors report an association with severity in a retrospective and targeted analysis. This is consistent with but does not prove a functional role as claimed and likely many other genes are equally associated*".

We thank the reviewer for raising this important point. Importantly, our analysis illustrates that the clinical effects of mTOG are independent from other genetic alteration in MDS (Fig. Extended Data Fig. 5). Based on our results that dysregulation of PUS7 and mTOG occurs even in the absence of chromosome 7 loss (Extended Data Fig. 4b,c) and overwhelming evidence that aberrant translation promotes cancer development (Robichaud et al., Cold Spring Harb Perspect Biol. 2019), we speculate that perturbation of the PUS7/mTOG/PABPC1 molecular pathways may provide a broader mechanism for leukemic transformation associated with stem cell and protein synthesis dysfunctions.

Response to Reviewer #3

We are delighted that the reviewer finds that "*The authors present compelling data that stem-cell enriched tRNA derived fragments (mTOG) and their role in regulating protein translation*".We thank the reviewer for recognizing that our "*new mechanistic studies describing a link between mTOG, PABPC1, and PAIP1 are convincing and supported by rigorous studies*" andfor his/her constructive comments. As suggested, we have strengthened our analysis of the contribution of mTOG for the regulation of key molecular and phenotypic properties of high- risk MDS (Figs. 3, 4 and Extended Data Figs. 6, 9 and 10).

Response to the specific comments.

1. The reviewer asks:" *It is unclear how transient transfection of the pseudouridylated mTOGsachieves durable suppression of translation in primary MDS cells over several days (Fig 3-4)? What is the transfection efficiency? How long do the MDS cells maintain expression of the oligos?*".

In agreement with on our previous mTOG characterization in PUS7-KO hESCs that synthetic mTOG-4′′ levels are sustained over several days (Guzzi et al. Cell 2018), we detected robust mTOG expression from 3-to-15 days post transient transfection of $CD34^+$ primary donor and MDS-patient derived HSPCs in culture (expansion) and upon *in vitro* erythroid and myeloid differentiation (Extended Data Figs. 6a and 9a). In our hands, mTOG-4′′ transient transfection yields > 10-fold excess of these small RNAs as compared to their endogenous levels, which ensures long-lasting effects on translation and differentiation.

2. The reviewer states that" *The concept that MDS erythroid cells exhibit increased translationis not*

necessarily consistent with prior literature. It is counter intuitive that decreasing proteintranslation will result in increased MDS erythroid differentiation (Fig 4). In fact, many subtypesof MDS exhibit aberrant ribosome protein expression and as a result reduced translation rates".

Our protein synthesis analysis illustrates higher translation rates in HR-MDS HSPC, which agrees with previous findings (Stevens et al., Nat Comm 2018). However, we did not assess translation in MDS-derived erythroid cells and apologize for the misleading heatmap (now removed from the figure) that reported our FC translation changes in HSPCs for each HR- MDS patient and healthy control analyzed. Moreover, we would like to emphasize that mTOG-4′′ treatment (or siPAIP1), selectively restores translation rates in HR-MDS HSPCs to those of healthy controls/LR-MDS patients, thus favoring erythroid differentiation of these cells (Fig. 4e). These results are consistent with our previous published findings that increased translation in PUS7-depleted human HSPC impairs erythroid/myeloid differentiation, which isrescued solely upon mTOG-4′′ transduction (Guzzi et al., Cell 2018).

3. The referee points that" The *immunoblots in Figure 3d should be on a single gel in order todraw conclusions on subtle changes in protein expression between the groups*".

Indeed, the protein analysis in Fig. 3d was done on a single gel (Source Data Fig. 8). In the figure, we simply separated the different patient categories with squares to better highlight the differences between the groups.

4. The reviewer comments" There *are significant concerns with the MDS xenograft model*" and asks" Why *is there no engraftment of HR-MDS samples when treated with SCR oligo (Fig4g)?".*

Previous studies have shown variable MDS engraftment levels in NSG, NSG-S and MISTRG models (Medyouf et al., Cell Stem Cell 2014; Rouault et al., Leukemia 2017; Song et al., Nat Comm 2019). We would also like to remark that our engraftment analysis was carried out as early as 8 weeks post-transplantation to ensure lasting mTOG-4′′ efficacy *in vivo* (Fig. Extended data fig. 6a). Importantly, we have included xenotransplants using low expressing PUS7/mTOG HR-MDS CD34^{+} cells from two additional independent patients. The results from these experiments show engraftment levels comparable to those previously reported (Medyouf et al., Cell Stem Cell 2014). Taken together, these findings strengthen our analysisand demonstrate that mTOG-4′′ transduction restores translation and significantly improves (~2 fold) HR-MDS HSPC multilineage engraftment in multiple HR-MDS patients (Fig. 4g-i).

4.1 He/she adds that "*Given that the engraftment is effectively at zero, it is also impossible tointerpret the data in panel 4i. It is also counterintuitive as to why there is increased engraftmentof HR-MDS cells treated with mTOG oligos. Based the proposed importance of mTOG- PABC1-PAIP1 in MDS, I would expect that expression of mTOG oligos would eliminate/decrease the HR-MDS clone, as would be the goal therapeutically, not to increase its engraftment potential*".

Although we do not have direct evidence that mTOG affects MDS clonal distribution, our results indicate that treatment with these small RNAs robustly target in 3/3 patients a critical population

of CD123+ malignant HR-MDS HSPC characterized by abnormally higher levels of protein biosynthesis (Fig. 4j) (Stevens et al., Nat Comm 2018). Taken together, our *in vitro* and *in vivo* results indicate that mTOG-4′′ restores multilineage engraftment mostly increasingdifferentiation toward lymphoid (CD19+ B cells) but not myeloid/leukemic (CD33+) lineages as in low-risk MDS patients (Fig. 4h,i and Extended data Fig. 10). These results are consistentwith previous findings that non-leukemic grafts are multilineage and enriched in CD19+ B cells(Slush et al., Nature, 2014), strongly suggesting that mTOG-mediated therapeutic inhibition ofoncogenic translation may be beneficial for the treatment of difficult-to-treat high risk MDS patients.

Decision Letter, first revision:

Date:	16th September 21 20:23:05
Last Sent:	16th September 21 20:23:05
Triggered By:	Jie Wang
From:	jie.wang@nature.com
To:	Cristian.Bellodi@med.lu.se
Subject:	Decision on Nature Cell Biology submission NCB-B44478A-Z
Message:	Dear Dr Bellodi,

Thank you for your email asking us to reconsider our decision on your manuscript, "Pseudouridine-modified tRNA fragments repress malignant protein synthesis and predict leukemic progression in myelodysplastic syndrome". We are always willing to hear the authors' perspective, but we must first prioritize decisions on new submissions. We appreciate your patience while we considered this appeal.

I have now discussed your manuscript and the referees’ comments and your rebuttal, in detail with my colleagues, and we would be willing to reconsider a revised manuscript provided the following issues can be addressed, and that nothing similar is accepted for publication at Nature Cell Biology or published elsewhere in the meantime.

In particular, please provide an updated rebuttal with responses to the referee comments "verbatim", not to key comments selected from the reports.

In addition, please pay close attention to our guidelines on statistical and methodological reporting (listed below) as failure to do so may delay the reconsideration of the revised manuscript. In particular please provide:

- a Supplementary Figure including unprocessed images of all gels/blots in the form of a multi-page pdf file. Please ensure that blots/gels are labeled and the sections presented in the figures are clearly indicated.

- a Supplementary Table including all numerical source data in Excel format, with data for different figures provided as different sheets within a single Excel file. The file should include source data giving rise to graphical representations and statistical descriptions in the paper and for all instances where the figures present representative experiments of multiple independent repeats, the source data of all repeats should be provided.

On resubmission please provide the completed Editorial Policy Checklist (found here https://www.nature.com/documents/nr-editorial-policy-checklist.pdf), and Reporting Summary (found here https://www.nature.com/documents/nr-reporting-summary.pdf). This is essential for reconsideration of the manuscript and these documents will be available to editors and referees in the event of peer review. For more information see below. Please also ensure that the presentation of statistical information in the revised submission complies with Nature Cell Biology's statistical guidelines (see below).

Please use the link below to submit the complete manuscript files and include a point-by-point response to the complete reviewer comments, verbatim as provided in their reports.

[REDACTED]

Please let us know how you wish to proceed and when we can expect your revised manuscript.

With kind regards,

Jie Wang

Jie Wang, PhD
Senior Editor
Nature Cell Biology

Tel: +44 (0) 207 843 4924
email: jie.wang@nature.com

GUIDELINES FOR EXPERIMENTAL AND STATISTICAL REPORTING

REPORTING REQUIREMENTS – To improve the quality of methods and statistics reporting in our papers we have recently revised the reporting checklist we introduced in 2013. We are now asking all life sciences authors to complete two items: an Editorial Policy Checklist (found here

https://www.nature.com/documents/nr-editorial-policy-checklist.pdf) that verifies compliance with all required editorial policies and a reporting summary (found here https://www.nature.com/documents/nr-reporting-summary.pdf) that collects information on experimental design and reagents. These documents are available to referees to aid the evaluation of the manuscript. Please note that these forms are dynamic ‘smart pdfs’ and must therefore be downloaded and completed in Adobe Reader. We will then flatten them for ease of use by the reviewers. If you would like to reference the guidance text as you complete the template, please access these flattened versions at http://www.nature.com/authors/policies/availability.html.

STATISTICS – Wherever statistics have been derived the legend needs to provide the n number (i.e. the sample size used to derive statistics) as a precise value (not a range), and define what this value represents. Error bars need to be defined in the legends (e.g. SD, SEM) together with a measure of centre (e.g. mean, median). Box plots need to be defined in terms of minima, maxima, centre, and percentiles. Ranges are more appropriate than standard errors for small data sets. Wherever statistical significance has been derived, precise p values need to be provided and the statistical test used needs to be stated in the legend. Statistics such as error bars must not be derived from n<3. For sample sizes of n<5 please plot the individual data points rather than providing bar graphs. Deriving statistics from technical replicate samples, rather than biological replicates is strongly discouraged. Wherever statistical significance has been derived, precise p values need to be provided and the statistical test stated in the legend.

Information on how many times each experiment was repeated independently with similar results needs to be provided in the legends and/or Methods for all experiments, and in particular wherever representative experiments are shown.

We strongly recommend the presentation of source data for graphical and statistical analyses as a separate Supplementary Table, and request that source data for all independent repeats are provided when representative experiments of multiple independent repeats, or averages of two independent experiments are presented. This supplementary table should be in Excel format, with data for different figures provided as different sheets within a single Excel file. It should be labelled and numbered as one of the supplementary tables, titled “Statistics Source Data”, and mentioned in all relevant figure legends.

Author Rebuttal, first revision:

Point-by-point response to reviewers' comments

Reviewer #1:

A. Summary

This study describes a previously unknown mechanism of translational control mediated by mTOG, a family of tRNA fragments. Here, the authors seek to mechanistically dissect this pathway and interrogate its possible role in tumorigenesis.

B. Originality and significance

This work builds on an earlier study by the authors. This on its own is not a major concern as the pathway described is distinct. However, I don't think the findings are in their current state rigorous enough to be of broad significance.

C. Data & methodology

My main concern with this study is that in its current form, it has made up its mind from the very beginning about the story that it wants to tell. Therefore, numerous alternative hypotheseshave not been considered, and the pathway structure is not always rigorously established. More specific concerns are discussed below.

We thank the reviewer for recognizing the novelty and significance of our study and for suggesting additional experiments, which greatly helped us improve our manuscript. As suggested, we rigorously reviewed all the statistical analyses, included complete mRNA quantifications, and measured tRNA levels upon mTOG treatment. Most importantly, we have performed additional experiments that broaden the significance of our findings by strengthening the links between the molecular and clinical parts of the paper, which unambiguously demonstrate the importance of the PUS7/mTOG/PAIP1 pathway for the regulation of central metabolic and phenotypic features of malignant MDS-HSPCs.

D. Appropriate use of statistics

There are some concerns about the visualization and analyses of the presented data. Below,I have listed some of these:

Fig 1C: From the figure, it seems that the number of replicates is 2, SD or SEM is meaninglessfor 2 data points.

Fig 1D: Not convincing. Effect size small and U-test (which is the best method for comparing 4 vs 4) would not be significant.

Fig 2B: Consideration of statistical significance is missing. Repeat the analysis with a specialized comparative tool (e.g. xtail or babel) and show volcano plots. Especially for the 3 selected genes.

Fig 2f: analysis is not done correctly. Normalizing reps to 1 has resulted in WT values to be

1.0 with SD of 0. Artificially low SD in the WT cohort impacts ANOVA p-values.In general, dynamite plots should be avoided.
This list is by no means comprehensive. I suggest every figure be examined for correct application of statistical analyses.

As recommended by the reviewer, we revised and updated data presentation and analysis for several panels, including Figs 1c-d, 2b, 2e-g, 3d-e, 4b, 4e, 4h-i, and Extended data figs. 1a, 2a-d, 3c, and 8a. Specifically, we have addressed the individual points below:

In figure 1c, we have included three replicates and applied a t-test with Welch correction, which provides a statistically significant p-value (0.0415) between control RNA and mTOG-∴ϑ. Conversely, no statistical significance is observed when control RNA and SCR-∴ϑ are compared.

In figure 4d, we applied the Mann-Whitney U test for the WT vs. PUS7-KO comparison, which shows a significant difference with p=0.0286 as stated in the text (page 4). In contrast, no statistically significant differences are observed when comparing WT and PUS7-KO cells treated with mTOG-λλ′ (p>0.99). We updated the figure accordingly.

For figure 2b, we included the consideration of statistical significance related to ribosome profiling on page 4. Furthermore, we would like to clarify that the rationale for selecting candidate genes is based on the remarkable enrichment for functional categories such as “translation” and “translation initiation” observed in PUS7-KO cells and the relevance for MDS pathogenesis. As suggested by the reviewer, we tried Xtail and Babel on our datasets; however, we did not obtain meaningful results using these pipelines. We speculate that these comparative tools are not particularly suitable for analysis of fewer than three replicates due to low statistical power and, in a situation of unidirectional global changes in translation (increase or decrease) as discussed by McGlincy and Ingolia Methods 2017.

Following the reviewer’s suggestion, we updated Fig. 2f with *de novo* protein synthesis shownas the $\log_2$ FC PUS7-KO/WT, which eliminates the normalization bias introduced by having the WT replicates set to 1 and a low SD. We included the individual data points in this figure and all the other bar plots throughout the primary and supplementary figures to provide complete information on the results.

E. Conclusions

Overall, the conclusions are supported by the observations. However, my biggest concern with this study is that in many places throughout the paper protein levels and translation efficiency are used interchangeably. Almost every experiment presented requires inclusion ofmRNA measurements to exclude transcript levels as a co-variate, which is missing. Absent this information, it is difficult to tease apart indirect from direct impact of translation.

As suggested by the reviewer, we have included the mRNA measurements along with the protein analysis in Figs. 2e, 2g, 3d, 3g, which demonstrate the specific translation regulation

for each candidate gene regulated downstream of the PUS7/mTOGs/PAIP1 axis in hESC, human MDS-L cells, and primary patient-derived MDS bone marrow cells (Fig. 3d-h). As stated in the manuscript (page 4), genes with transcription changes (|log2 FC mRNA KO/WT| > 1) were excluded from the analysis of translationally regulated genes in PUS7-KO cells (Fig. 2b). Moreover, we have included additional experiments illustrating that translation control by mTOG-$\lambda\lambda'$ involves the 5' PES motif embedded with the 5' UTRs of RPL23, RPL29, and EIF6 transcripts identified using ribosome profiling (Figs 2h-j and Extended Data Fig. 3d).

F. Suggested improvements:

In addition to the above (i.e., inclusion mRNA measurements; e.g. in fig 2e and g: repeat with qPCR for the mRNAs to show that the effects are due to translational changes), another major concern is that the study is quite disjointed. There is molecular part and a cancer part. The two don't communicate. For example, the components of the pathway described in the first sections are not then rigorously studied for their requirement and sufficiency in the cancer side. I would propose the following experiments:

Following the reviewer suggestion, we have extended our analysis of PUS7, mTOG, and PAIP1 to extensively validate the molecular and functional epistasis between the pathway components in patient-derived primary MDS stem cells and the human MDS-derived cell line, MDS-L (Tohyama et al., Br J Haematol. 1995) (Fig. 3 e-h and 4b-e). These new results (highlighted on page 7) tightly link the molecular components identified in the first section to the clinical findings presented in Figs 3 and 4.

Fig 3a: The levels of tRNAs should be included as a co-variate (here and throughout the study); also included should be the genes discussed here (e.g. PAIP1); there should a significant interaction term in a multivariate cox-ph analysis.

The analysis in Fig. 3a was performed using a limited amount of total RNA obtained from a small population of primary MDS hematopoietic stem and progenitor cells. As such, we are unable to comprehensively investigate tRNA levels in this patient cohort. However, we have included northern blot analysis for tRNA-Tyr (GUA), a well-established PUS7 target (Behn-Ansmant et al., RNA 2003, Urban et al., J Biol Chem 2009), in PUS7-KO hESCs and MDS-L cells in the absence or presence of mTOGs. Consistent with our previous tRNA quantification (Guzzi et al., Cell 2018) and codon usage analysis (Extended Data Fig. 2d) illustrating no significant differences in *PUS7-KO* cells, we found that PUS7 loss and mTOG transfection did not alter the levels of tRNA-Tyr (GUA) in any of the cell system tested (Extended Data Figs. 2h, 7a). Of note, similar results were obtained comparing primary $CD34^-$ bone marrow mononuclear cells (BMMNC) from two distinct HR-MDS patients with low PUS7 and mTOG levels to two age-matched healthy donors (Extended Data Fig. 7a).

As requested, we analyzed PAIP1 and PABPC1 mRNA levels by ddqPCR within the same MDS patient cohort (n=50) used for PUS7 and mTOG quantification and applied the multivariate Cox model controlled for age (Supplementary Table 6). This analysis shows that PUS7, mTOG, and PABPC1 have independent and additive effects on survival. Notably, PABPC1 was not correlated with mTOG, whereas PAIP1 positively correlated with mTOG

(R=0.35, p=0.013) and PABPC1 (R=0.36, p=0.011). However, PAIP1 did not reach statistical significance in the Univariate Cox´s model (p=0.155). Therefore, it was excluded from the multivariate analysis. Together these findings suggest that dysregulation of the pathway eitherat the level of PUS7/mTOG or through perturbations of other components such as PABPC1 leads to the same prognostic effects in MDS.

Fig 3d: Show RNA results; show results are PAIP1 dependent.

We employed siRNA pools to deplete PAIP1 and selectively rescue increased bulk protein synthesis and translation of PES-containing mRNAs in primary patient-derived MDS cells and PUS7-depleted MDS-L cells (Fig. 3f-h). Consistent with our findings in hESCs, we show that mTOG translation-repressive function is exquisitely dependent on PAIP1 in these cells.

Fig 4: The most important piece of data missing here is epistasis. This is needed to show that the mTOG's effect is mediated through the identified pathway.

As suggested, we performed additional experiments illustrating that PAIP1 depletion significantly improves differentiation of HR-MDS stem cells in CFU assays and phenocopy the effects of mTOG transduction in these cells (Fig. 4b). Of note, we observed similar effects in response to mTOG and siPAIP1 transfection in MDS-L cells (Fig. 4c), which further highlightsan epistatic interaction between these pathway components in MDS.

Does exogenous mTOG has an impact of tRNA content of the cell?

As mentioned above, we assessed the effects of mTOG transfection on tRNA-Tyr (GUA) levels and found no differences in either hESCs or MDS-L cells (Extended Data Figs. 2h and7a).

Reviewer #2:

A rather innovative topic and also a follow up on the authors' recent Cell paper that reports a role of mTOG, a small tRNA fragment with potential roles in stem cell biology that are mediatedby mTOG $\lambda\lambda'$ binding to PABPC1.

We are pleased that the reviewer finds that our work represents "*A rather innovative topic*" and highlights that our new story "*reports a role of mTOG, a small tRNA fragment with potentialroles in stem cell biology that are mediated by mTOG $\lambda\lambda'$ binding to PABPC1*". We thank the reviewer for his/her comments, which helped us strengthen the manuscript. In response to thereviewer's concerns, we have address each of the issues raised.

The authors claim the following main points:

1. A selective effect of mTOG 4′′ on oncogenic translation programs. However, their ribosomeprofiling study indicates a broad, not a selective effect on translation.

The ribosome profiling analysis is consistent with our previous finding that PUS7 and mTOG impairments are associated with perturbation of bulk protein synthesis (Guzzi et al., Cell 2018). Furthermore, we provide compelling evidence that mTOG-4′′ directly fine-tunes translation of functionally distinct mRNA subsets enriched for ribosomal proteins and translation factors (Fig. 2c). Critically, our new results indicate that this effect is at least in part mediated through the regulation of the PES motif embedded in the 5' UTR of these mRNAs (Fig. 2d, 2h-j, Extended data Fig. 3d), which may provide an additional layer of regulation to control ribosome biogenesis and translation. Since dysregulation of ribosome biogenesis and translation is associated with malignant transformation downstream of prominent oncogenic signaling pathways, including PI3K/AKT/mTOR, MYC, and RAS/MAPK (Gentilella et al., BiochimBiophys Acta. 2015; van Riggelen et al., Nat Rev Cancer 2010; Shaw et al., Nature 2006; Truitt et al., Nat Rev Cancer 2016), our findings highlight a key role for dysregulation of PUS7/mTOG in driving aberrant protein synthesis in distinct stem and cancer cell populations.

2. Effect is mediated by interaction of mTOG 4′′ with PABPC1 that impair binding of PAIP1 andinhibition of the eIF4F complex.EMSA and a MS technique and pulldowns are used to show confirm and map the first interaction. It is not clear what other proteins mTOG 4′′ would bind, and therefore what is the specific role of PABPC1 and the specific binding site. The effect onPAIP1 is compared to PAIP2 (not affected) establishing a level of selectivity.

We previously showed that several proteins might bind mTOG-4′′ (Guzzi et al., Cell 2018); however, our *in vitro* and *in vivo* analysis compellingly demonstrate that the molecular interactions between mTOG, PABPC1, and PAIP1 (Fig. 1) are sufficient and critically requiredto enable control of bulk and mRNA-specific translation downstream of PUS7 in hESCs, MDS-L cells and HSPCs (Fig. 2e,g, 3c, 3e-g, Extended Data Fig. 8b). Additionally, we provide new evidence that depletion of PAIP1 restores translation and improves differentiation of PUS7-depleted MDS-L cells and HR-MDS HSPCs, thus functionally phenocopying mTOG-4′′ transduction in these cells (Figs. 4b,c). Taken together, these results provide a mechanistic explanation to our previous findings that mTOG-PABPC1 interaction is specific and directly dictates protein synthesis and stem cell lineage commitment downstream of PUS7 in embryonic and hematopoietic stem cells.

The authors also use PUS7 k/o cells. It is not clear to what extent PUS7 ko cells have a specific defect in mTOG 4′′ versus more broad changes in 4′′. Together, the authors show binding anda potential mechanism, they do not address the sufficiency/requirement and potential role of other binding partners.

Although it is possible that PUS7 loss may affect 4′′ modifications of other RNAs, including the anticodon position U35 in tRNA-Tyr (GUA) (Urban et al., J Biol Chem 2009; Behm-Ansmant et al., RNA 2003), our results that PUS7 selectively targets 4′′ to control mTOGs activity in hESCs and HSPCs, provide a unique biologically relevant context for the function of the

PUS7/mTOG axis in translation control. Importantly, we demonstrate that reintroduction of mTOG-4′′ fully restores protein synthesis and stem cell differentiation in PUS7-KO hESCs and HSPC (Guzzi et al., Cell 2018). Importantly, we provide evidence that similar mTOG effects were selectively observed in primary HR-MDS HSPCs and upon PUS7 depletion in the MDS-derived cell line, MDS-L, characterized by aberrantly high levels of translation (Figs. 3c, 3g,h, and 4b-e). Our molecular analysis illustrates that mTOG-4′′ binding to PABPC1 RRM2-3 and regulation of PAIP1 as a primary mechanism for translation control in hESCs and HSPCs. Our results that mTOG-4′′ transduction, depletion of PABPC1 (Guzzi et al., Cell 2018 and this manuscript Extended Data Fig. 3a) or PAIP1 (Figs. 2f,g, and 3f,g) selectively restores protein synthesis in PUS7-KO cells provide strong evidence that the epistatic interaction between these components is functionally sufficient to define a distinct molecular pathway governing translation initiation in stem cells.

3. Loss of this interaction increases translation of mRNAs with specific features referred to asPES and that include TOP sequence elements.

The authors report a ribosome profiling study on PUS k/o cells. They report a “global increase” and also refer to >2000 differentially translated mRNAs with a higher fold-change than others. The authors do not discuss the effect of Pus7 loss on gene expression, the statists of this analysis is questionable with a q value of 0.25. The actual q/FDR values are not provided.

Our analysis of translation was based on the criterion that no significant changes in mRNA expression ($-1< \log_2$ FC mRNA KO/WT <1) were detected for the individual genes, we specify this in the text (page 4) and the methods. Our results are consistent with our previous studies that PUS7 depletion primarily alters translation in the absence of transcriptional effects (Guzziet al., Cell 2018). Significantly, we have improved our Ribo-seq analysis mapping the reads on the more recent GENCODE 35 release and applying more stringent cut-offs for translation efficiency (Log_2 FC TE KO/WT > 1) and FDR < 0.2 (Fig. 2b,c). As requested, we have included the FDR values for the ribosome profiling study in Supplementary Table S2.

The inset in 2B appears to show a general shift in translation and not a specific effect. Among mRNAs that show increased translation the authors identify an enrichment of GC rich mRNAs with short UTRs and TOP and TOP like mRNAs. It is not examined which RNAs are relatively less translated.

We examined 949 relatively less translated transcripts with $\log_2$ FC TE < 0.6415 correspondingto the bottom 15% TE FC and FDR < 0.2 in PUS7-KO cells, classified within functional categories related to transcription and splicing. Notably, we could not find enrichment for ribosome/translation-related functional categories nor 5’ PES-containing mRNAs (p>0.05) among these relatively less translated transcript fractions in PUS7-KO cells.

4. PES and TOP mRNAs include many ribosomal proteins and other translation factors andhave been linked to mTOR signaling and a global increase in translation capacity.

This is consistent with a global effect on translation and contradict a selective effect on an oncogenic translation program claimed above. as this program is not thought to be cancer specific, but rather a broad effect on all/most mRNAs.

We agree that several 5’ PES/TOP containing mRNAs identified in our ribosome profiling analysis have been linked to mTOR signaling and regulation of global protein synthesis. Indeed, we previously showed that PUS7 and mTOG impairments were associated with perturbation of bulk translation (Guzzi et al., Cell 2018). Building on these original observations, our ribosome profiling analysis reveals remarkable changes at the translation level of individual 5’ PES-containing transcripts (Figs. 2e, 2g-j and Extended Data Fig. 3d). These data are in line with recent evidence highlighting that regulation of these 5’TOP/TOP-like mRNAs downstream of mTORC1 is complex and likely involves multiple cell-type-specific mechanisms (Philippe et al., PNAS; Cockman et al., Biomolecules 2020). Importantly, we find that 5’ PES-containing mRNAs including RPL23, RPL29, eIF6 are exquisitely dependent on mTOG-4′′ and PAIP1 in PUS7-depleted hESCs, MDS-L and HR-MDS HSPCs (Fig. 2e-j, 3e-h, Extended Data Figs. 3d and 8b). Furthermore, this defines a unique dependency for translation programs that selectively steer the metabolism of distinct stem and cancer cell populations. Our results may provide a mechanistic explanation to findings that several 5’ TOP mRNAs are weakly sensitive to mTORC1 activity and LARP1-mediated regulation (Philippe et al., PNAS 2020).

The authors highlight the role of ribosomal protein RPL23 that had been linked to delayed leukemia development. Genetic epistasis studies confirm effects on various ribosomal proteins. These data do not, in my mind, support the proposed malignant translation programbut are more consistent with a global effect on translation.

As stated above (reviewer #2 point 1), the defects in 5’PES mRNA translation reported in our study highlight ribosome biogenesis and protein biosynthesis as major cancer-promoting programs directly altered downstream of PUS7/mTOG in HR-MDS.

5. A role for mTOG 4′′ in MDS-derived HSCs and their malignant progression.
The authors indicate mTOG 4′′ represses protein production without effect on viability or proliferation, which seems hard to believe. Effects on translation on specific proteins seem variable and actin is among the most altered proteins.

We would like to emphasize that mTOG-4′′ transduction (or PAIP1 depletion) does not block protein synthesis but instead restores translation in HR-MDS HSPCs to levels observed in normal hematopoietic and LR-MDS HSPC and without effects on viability and proliferation (Figs. 3c and Extended data Fig. 6b-e). This is consistent with our previous findings in hESCs and HSPCs that mTOG has no effects on viability and proliferation (Guzzi et al., Cell 2018), and with results that differences in protein synthesis are uncoupled from changes in HSPCs cell division (Signer et al., Genes & Development 2016). Following the reviewer's suggestion, we have included another housekeeping gene, GAPDH, as a control for the protein analysis of PUS7/mTOG-regulated mRNAs in primary MDS patient samples (Fig. 3d).

Colony assays indicate mTOG 4′′ enhances colonies. Engraftment in animals are less convincing than described as 2/5 mTOG 4′′ animals seem to have rather low engraftment and ¼ controls has good engraftment. This is not convincing.

In response to this point and comments from referee #3, we have expanded our analysis *in vivo,* including xenotransplantation of $CD34^+$ cells derived from two additional HR-MDS and one extra LR-MDS patients (Fig. 4f-j and Extended data Fig. 10a,b). Our new results illustrate engraftment levels comparable to those achieved in previous studies (Medeyouf et al., Cell Stem Cell 2014). Notably, our findings are consistent with our original observations that mTOG selectively enhanced engraftment (>2 fold) and “non-leukemic” multilineage differentiation of HR MDS-HSPC but not in LR-MDS (Fig. 4g-i and Extended data Fig 10a,b). These effects, coupled with significant reductions in the frequency of $CD123^+$ leukemic stem cells (Fig. 4j), compellingly support our findings that mTOG target malignant stem cell populations characterized by abnormally high protein synthesis levels (Stevens et al., Nat Comm 2018).

6. A clinical association between mTOG 4′′ and disease severity/progression in MDS/AML.

The link to monosomy7 is interesting. However, another gene (SAMD9) has been clearly implicated in mono7 MDS. https://www.ncbi.nlm.nih.gov/pmc/articles/PMC5540771/ PUS7 is indeed on Chr. 7 and has been linked to a developmental disorder. Is there direct genetic evidence that this pathway and its key enzymes are involved in leukemia? The authors cite a general review and their own work. However, their cell paper only shows reduced PUS7 in monosomy7 cells not a role in disease. The authors report an association with severity in a retrospective and targeted analysis. This is consistent with but does not prove a functional role as claimed and likely many other genes are equally associated.

We thank the reviewer for raising this important point. Importantly, our analysis illustrates that the clinical effects of mTOG are independent from other genetic alteration in MDS (Fig. Extended Data Fig. 5). Based on our results that dysregulation of PUS7 and mTOG occurs even in the absence of chromosome 7 loss (Extended Data Fig. 4b,c) and overwhelming evidence that aberrant translation promotes cancer development (Robichaud et al., Cold Spring Harb Perspect Biol. 2019), we speculate that perturbation of the PUS7/mTOG/PABPC1 molecular pathways may provide a broader mechanism for leukemic transformation associated with stem cell and protein synthesis dysfunctions.

Reviewer #3:

The authors present compelling data that stem-cell enriched tRNA derived fragments (mTOG) and their role in regulating protein translation. This submission builds upon the group’s previous findings on pseudouridylation of mTOGs in embryonic stem cells (Guzzi, Cell 2018).

They showed that the pseudouridylation "writer" PUS7 modifies and activates a novel networkof mTOGs targeting the translation initiation complex. PUS7 inactivation in embryonic stem cells impairs tRF-mediated translation regulation, leading to increased protein biosynthesis and defective germ layer specification. In this submission, they extended these mechanistic studies to pre-leukemic hematopoietic cells. Herein, they showed that mTOG targets PABC1,which hinders the recruitment of PAIP1 and translation of transcripts sharing 5-UTR pyrimidine-enriched sequences. Lastly, they show that mTOG dysreulgation is implicated in MDS HSPCs and may affect leukemic transformation. Overall, the new mechanistic studies describing a link between mTOG, PABPC1, and PAIP1 are convincing and supported by rigorous studies (Figure 1-2). However, the potential role of mTOG dysregulation in the pathogenesis of MDS is weak and not supported by the current data.

We are delighted that the reviewer finds that “*The authors present compelling data that stem-cell enriched tRNA derived fragments (mTOG) and their role in regulating protein translation*”.We thank the reviewer for recognizing that our “*new mechanistic studies describing a link between mTOG, PABPC1, and PAIP1 are convincing and supported by rigorous studies*” andfor his/her constructive comments. As suggested, we have strengthened our analysis of the contribution of mTOG for the regulation of key molecular and phenotypic properties of high- risk MDS (Figs. 3, 4, and Extended Data Figs. 6, 9, and 10).

- It is unclear how transient transfection of the pseudouridylated mTOGs achieves durable suppression of translation in primary MDS cells over several days (Fig 3-4)? What is the transfection efficiency? How long do the MDS cells maintain expression of the oligos?

In agreement with our previous mTOG characterization in PUS7-KO hESCs that synthetic mTOG-4′′ levels are sustained over several days (Guzzi et al. Cell 2018), we detected robust mTOG expression from 3-to-15 days post transient transfection of $CD34^+$ primary donor and MDS-patient derived HSPCs in culture (expansion) and upon *in vitro* erythroid and myeloid differentiation (Extended Data Figs. 6a and 9a). In our hands, mTOG-4′′ transient transfection yields > 10-fold excess of these small RNAs compared to their endogenous levels, which ensures long-lasting effects on translation and differentiation.

- The concept that MDS erythroid cells exhibit increased translation is not necessarilyconsistent with prior literature. It is counter intuitive that decreasing protein translation will result in increased MDS erythroid differentiation (Fig 4). In fact, many subtypes of MDS exhibit aberrant ribosome protein expression and as a result reduced translation rates.

Our protein synthesis analysis illustrates higher translation rates in HR-MDS HSPC, which agrees with previous findings (Stevens et al., Nat Comm 2018). However, we did not assess translation in MDS-derived erythroid cells and apologize for the misleading heatmap (now removed from the figure) that reported our FC translation changes in HSPCs for each HR-MDS patient and healthy control analyzed. Moreover, we would like to emphasize that mTOG-4′′ treatment (or siPAIP1) selectively restores translation rates in HR-MDS HSPCs to healthy

controls/LR-MDS patients, thus favoring erythroid differentiation of these cells (Fig. 4e). These results are consistent with our previously published findings that increased translation in PUS7-depleted human HSPC impairs erythroid/myeloid differentiation, which is rescued solelyupon mTOG-4′′ transduction (Guzzi et al., Cell 2018).

- The immunoblots in Figure 3d should be on a single gel in order to draw conclusions on subtle changes in protein expression between the groups.

Indeed, the protein analysis in Fig. 3d was done on a single gel (Source_data_blots_pg. 3). In the figure, we simply separated the different patient categories with squares to better highlight the differences between the groups.

- There are significant concerns with the MDS xenograft model. Why is there no engraftmentof HR-MDS samples when treated with SCR oligo (Fig 4g)?

Previous studies have shown variable MDS engraftment levels in NSG, NSG-S, and MISTRG models (Medyouf et al., Cell Stem Cell 2014; Rouault et al., Leukemia 2017; Song et al., Nat Comm 2019). We would also like to remark that our engraftment analysis was carried out as early as eight weeks post-transplantation to ensure lasting mTOG-4′′ efficacy *in vivo* (Fig. Extended data fig. 6a). Importantly, we have included xenotransplants using low expressing PUS7/mTOG HR-MDS CD34$^+$ cells from two additional independent patients. The results from these experiments show engraftment levels comparable to those previously reported (Medyouf et al., Cell Stem Cell 2014). These findings strengthen our analysis and demonstratethat mTOG-4′′ transduction restores translation and significantly improves (~2 fold) HR-MDS HSPC multilineage engraftment in multiple HR-MDS patients (Fig. 4g-i).

Given that the engraftment is effectively at zero, it is also impossible to interpret the data in panel 4i. It is also counterintuitive as to why there is increased engraftment of HR-MDS cells treated with mTOG oligos. Based the proposed importance of mTOG-PABC1-PAIP1 in MDS,I would expect that expression of mTOG oligos would eliminate/decrease the HR-MDS clone,as would be the goal therapeutically, not to increase its engraftment potential.

Although there is no direct evidence that mTOG affects MDS clonal distribution, our results indicate that treatment with these small RNAs robustly target in 3/3 patients a criticalpopulation of CD123$^+$ malignant HR-MDS HSPC characterized by abnormally higher levels ofprotein biosynthesis (Fig. 4j) (Stevens et al., Nat Comm 2018). Taken together, our *in vitro* and *in vivo* results indicate that mTOG-4′′ restores multilineage engraftment, mainly increasingdifferentiation toward lymphoid (CD19+ B cells) but not myeloid/leukemic (CD33+) lineages as in low-risk MDS patients (Fig. 4h,i and Extended data Fig. 10). These results are consistentwith previous findings that non-leukemic grafts are multilineage and enriched in CD19$^+$ B cells(Slush et al., Nature, 2014), strongly suggesting that mTOG-mediated therapeutic inhibition ofoncogenic translation may be beneficial for the treatment of difficult-to-treat high-risk MDS patients.

Decision Letter, second revision:

Subject: Decision on Nature Cell Biology submission NCB-B44478B
Message:

*Please delete the link to your author homepage if you wish to forward this email to co-authors.

Dear Dr Bellodi,

Your manuscript, "Pseudouridine-modified tRNA fragments repress malignant protein synthesis and predict leukemic progression in myelodysplastic syndrome", has now been seen by the three original referees. As you will see from their comments, while reviewers 1 and 3 are in general satisfied with the revision, reviewer 2 continued to question whether the pseudouridine-modified tRNA fragments impact malignancy-specific translation and requested for additional in vivo studies to strengthen the link to MDS.

Nature Cell Biology editors discuss the referee reports in detail within the editorial team, including the chief editor, to identify key referee points that should be addressed with priority, and requests that are overruled as being beyond the scope of the current study. To guide the scope of the revisions, I have listed these points below. We are committed to providing a fair and constructive peer-review process, so please feel free to contact me if you would like to discuss any of the referee comments further.

a) With regard to the malignancy specificity concern, we have consulted with reviewer 1, who has overlapping expertise in translation. The feedback of this reviewer is included in the remarks to authors as extra comments. Considering the input from reviewer 1 and our own editorial discussion, we advise you to address this concern textually by acknowledging that the downstream effector genes of pseudouridine-modified tRNA fragments responsible for the cancer phenotype warrant future studies and toning down the claim that these fragments repress malignant protein synthesis.

Reviewer 2:
“The profiling data are good, but again they show a global effect on translation, not -as the title/abstract suggest- a malignancy specific effect. I raised the question before and the answer appears to be that all cap-dependent translation promotes cancer and is therefore cancer specific. I am not convinced by the argument or the reference to the RPL23 study”.

b) With regard to the request for further in vivo data, although we agree they would strengthen the link to MDS, this request represents a new point raised in this round and we consider it to be beyond the scope of the present study. Thus, addressing it experimentally will not be necessary for reconsideration of the manuscript at this journal. However, it would be necessary to revise the text to make sure the description of in vitro studies on MDS stem cells and the rescue studies is sufficient.

Reviewer 2:
“The link to MDS is based on retrospective associations, this is nice but hardly "compelling" evidence of a role in the disease. I find the description of in vitro studies on MDS stem cells and the rescue studies insufficient to understand. for the in vivo studies I would like to see evidence of mTOG expression and the sufficiency and requirements of the proposed molecular effects.”

c) We ask you to address the concern by Reviewer 1 regarding the large error bar of the mRNA measurements by adding more biological replicates:
“Having said that, I had asked the authors to provide mRNA measurements to rule out transcriptional changes as the source of observed modulations in protein levels. While they have done this, the error bars on their measurements, at times, exceeds what is customary (for example Fig 3h). When error bars are this large, it is not unexpected that the differences will not be statistically significant. Time/resources permitting, the authors should consider tightening these error bars.”

d) All other referee concerns pertaining to clarifications and textual changes should also be addressed.

e) Finally please pay close attention to our guidelines on statistical and methodological reporting (listed below) as failure to do so may delay the reconsideration of the revised manuscript. In particular please provide:

- a Supplementary Figure including unprocessed images of all gels/blots in the form of a multi-page pdf file. Please ensure that blots/gels are labeled and the sections presented in the figures are clearly indicated.

- a Supplementary Table including all numerical source data in Excel format, with data for different figures provided as different sheets within a single Excel file. The file should include source data giving rise to graphical representations and statistical descriptions in the paper and for all instances where the figures present representative experiments of multiple independent repeats, the source data of all repeats should be provided.

We therefore invite you to take these points into account when revising the manuscript. In addition, when preparing the revision please:

- ensure that it conforms to our format instructions and publication policies (see below and www.nature.com/nature/authors/).

- provide a point-by-point rebuttal to the full referee reports verbatim, as provided at the end of this letter.

- provide the completed Editorial Policy Checklist (found here https://www.nature.com/authors/policies/Policy.pdf),and Reporting Summary (found here https://www.nature.com/authors/policies/ReportingSummary.pdf). This is essential for reconsideration of the manuscript and these documents will be available to editors and referees in the event of peer review. For more information see http://www.nature.com/authors/policies/availability.html or contact me.

Nature Cell Biology is committed to improving transparency in authorship. As part of our efforts in this direction, we are now requesting that all authors identified as 'corresponding author' on published papers create and link their Open Researcher and Contributor Identifier (ORCID) with their account on the Manuscript Tracking System (MTS), prior to acceptance. ORCID helps the scientific community achieve unambiguous attribution of all scholarly contributions. You can create and link your ORCID from the home page of the MTS by clicking on 'Modify my Springer Nature account'. For more information please visit please visit www.springernature.com/orcid.

Please submit the revised manuscript files and the point-by-point rebuttal to the referee comments using this link:

[REDACTED]

*This url links to your confidential home page and associated information about manuscripts you may have submitted or be reviewing for us. If you wish to forward this email to co-authors, please delete the link to your homepage.

We would like to receive the revision within four weeks. If submitted within this time period, reconsideration of the revised manuscript will not be affected by related studies published elsewhere, or accepted for publication in Nature Cell Biology in the meantime. We would be happy to consider a revision even after this timeframe, but in that case we will consider the published literature at the time of resubmission when assessing the file.

We hope that you will find our referees' comments, and editorial guidance helpful. Please do not hesitate to contact me if there is anything you would like to discuss.

Best wishes,

Jie Wang

Jie Wang, PhD
Senior Editor
Nature Cell Biology

Tel: +44 (0) 207 843 4924
email: jie.wang@nature.com

Reviewers' Comments:

Reviewer #1:
Remarks to the Author:
I acknowledge that the authors had given it their best effort to address the points I had raised. So, while there were pieces of data I would've liked to see (and the analysis of Ribo-seq data not powered enough), I understand that sufficient material may not be at hand. Therefore, I recommend publication of this study.

Having said that, I had asked the authors to provide mRNA measurements to rule out transcriptional changes as the source of observed modulations in protein levels. While they have done this, the error bars on their measurements, at times, exceeds what is customary (for example Fig 3h). When error bars are this large, it is not unexpected that the differences will not be statistically significant. Time/resources permitting, the authors should consider tightening these error bars.

Minor:
Fig S4G, the points are missing and converted to crossed-out boxes. This happens when ggplot is imported to AI. Setting useDingbats to false in ggsave will solve this (or changing the font in AI)

Extra comments:
In some ways, my comment in the first round stating that there were two separate stories here, a translation story and a cancer story, was a similar criticism. But I'm not sure what reviewer 2 means by global; at least in case of Ribo-seq, the authors describe a regulon of a few thousand genes that are affected by this pathway. That number, while large, is not global (or if there is a global effect, it is more pronounced for these targets). What may be missing here (and liklely what reviewer 2 is asking), is the downstream effector genes that are responsible for the cancer phenotype. It goes without saying that the vast majority of changes observed are likely neutral and there are only a few genes whose increased expression is driving tumorigenesis. While this paper doesn't have these, and would've been a more complete story with them known, I think the identified master regulators are sufficiently well-characterized to warrant publication. In sum, I agree with reviewer 2 in general that the specific

downstream effects that are relevant to tumorigenesis are not described, but I think that this may be a high bar as the paper already contains a ton of new information and insights.

Reviewer #2:
Remarks to the Author:
The revised MS by Guzzi et al leaves many questions unanswered. I find data in Fig. 1 about binding to PABPC1 clear and convincing. It remains unclear what else the mTOG RNA would bind.
(PES is not introduced in the MS and I am not sure what it stands for, unless I missed it).

The profiling data are good, but again they show a global effect on translation, not -as the title/abstract suggest- a malignancy specific effect. I raised the question before and the answer appears to be that all cap-dependent translation promotes cancer and is therefore cancer specific. I am not convinced by the argument or the reference to the RPL23 study.

The link to MDS is based on retrospective associations, this is nice but hardly "compelling" evidence of a role in the disease. I find the description of in vitro studies on MDS stem cells and the rescue studies insufficient to understand. for the in vivo studies I would like to see evidence of mTOG expression and the sufficiency and requirements of the proposed molecular effects.

Limited data have been added to address prior concerns, and I am sorry to say, I find the MS cursory and not ready to publish in its revised form.

Reviewer #3:
Remarks to the Author:
The authors have done a solid job addressing my original concerns. In particular, they have sufficiently clarified their findings using the MDS primary samples (Figure 4). I have no additional comments.

GUIDELINES FOR SUBMISSION OF NATURE CELL BIOLOGY ARTICLES

READABILITY OF MANUSCRIPTS – Nature Cell Biology is read by cell biologists from diverse backgrounds, many of whom are not native English speakers. Authors should aim to communicate their findings clearly, explaining technical jargon that might be unfamiliar to non-specialists, and avoiding non-standard abbreviations. Titles and abstracts should concisely communicate the main findings of the study, and the background, rationale, results and conclusions should be clearly explained in the manuscript in a manner accessible to a broad cell biology audience. Nature Cell Biology uses British spelling.

ARTICLE FORMAT

TITLE – should be no more than 100 characters including spaces, without punctuation and avoiding technical terms, abbreviations, and active verbs..

AUTHOR NAMES – should be given in full.

AUTHOR AFFILIATIONS – should be denoted with numerical superscripts (not symbols) preceding the names. Full addresses should be included, with US states in full and providing zip/post codes. The corresponding author is denoted by: "Correspondence should be addressed to [initials]."

ABSTRACT – should not exceed 150 words and should be unreferenced. This paragraph is the most visible part of the paper and should briefly outline the background and rationale for the work, and accurately summarize the main results and conclusions. Key genes, proteins and organisms should be specified to ensure discoverability of the paper in online searches.

TEXT – the main text consists of the Introduction, Results, and Discussion sections and must not exceed 3500 words including the abstract. The Introduction should expand on the background relating to the work. The Results should be divided in subsections with subheadings, and should provide a concise and accurate description of the experimental findings. The Discussion should expand on the findings and their implications. All relevant primary literature should be cited, in particular when discussing the background and specific findings.

ACKNOWLEDGEMENTS – should be kept brief. Professional titles and affiliations are unnecessary. Grant numbers can be listed.

AUTHOR CONTRIBUTIONS – must be included after the Acknowledgements, detailing the contributions of each author to the paper (e.g. experimental work, project planning, data analysis etc.). Each author should be listed by his/her initials.

FINANCIAL AND NON-FINANCIAL COMPETING INTERESTS – the authors must include one of three declarations: (1) that they have no financial and non-financial competing interests; (2) that they have financial and non-financial competing interests; or (3) that they decline to respond, after the Author Contributions section. This statement will be published with the article, and in cases where financial and non-financial competing interests are declared, these will be itemized in a web supplement to the article. For further details please see https://www.nature.com/licenceforms/nrg/competing-interests.pdf.

REFERENCES – are limited to a total of 70 in the main text and Methods combined,. They must be numbered sequentially as they appear in the main text, tables and figure legends and Methods and must follow the precise style of Nature Cell Biology references. References only cited in the Methods should be numbered consecutively following the last reference cited in the main text. References only associated with Supplementary Information (e.g. in supplementary legends) do not count toward the total reference limit and do not need to be cited in numerical continuity with references in the main text. Only published papers can be cited, and each publication cited should be included in the numbered reference list, which should include the manuscript titles. Footnotes are not permitted.

METHODS – Nature Cell Biology publishes methods online. The methods section should be provided as a separate Word document, which will be copyedited and appended to the manuscript PDF, and incorporated within the HTML format of the paper.

Methods should be written concisely, but should contain all elements necessary to allow interpretation and replication of the results. As a guideline, Methods sections typically do not exceed 3,000 words. The Methods should be divided into subsections listing reagents and techniques. When citing previous methods, accurate references should be provided and any alterations should be noted. Information must be provided about: antibody dilutions, company names, catalogue numbers and clone numbers for monoclonal antibodies; sequences of RNAi and cDNA probes/primers or company names and catalogue numbers if reagents are commercial; cell line names, sources and information on cell line identity and authentication. Animal studies and experiments involving human subjects must be reported in detail, identifying the committees approving the protocols. For studies involving human subjects/samples, a statement must be included confirming that informed consent was obtained. Statistical analyses and information on the reproducibility of experimental results should be provided in a section titled "Statistics and Reproducibility".

All Nature Cell Biology manuscripts submitted on or after March 21 2016, must include a Data availability statement as a separate section after Methods but before references, under the heading "Data Availability". For Springer Nature policies on data availability see http://www.nature.com/authors/policies/availability.html; for more information on this particular policy see http://www.nature.com/authors/policies/data/data-availability-statements-data-citations.pdf. The Data availability statement should include:

• Accession codes for primary datasets (generated during the study under consideration and designated as "primary accessions") and secondary datasets (published datasets reanalysed during the study under consideration, designated as "referenced accessions"). For primary accessions data should be made public to coincide with publication of the manuscript. A list of data types for which submission to community-endorsed public repositories is mandated (including sequence, structure, microarray, deep sequencing data) can be found here http://www.nature.com/authors/policies/availability.html#data.

• Unique identifiers (accession codes, DOIs or other unique persistent identifier) and hyperlinks for datasets deposited in an approved repository, but for which data deposition is not mandated (see here for details http://www.nature.com/sdata/data-policies/repositories).

• At a minimum, please include a statement confirming that all relevant data are available from the authors, and/or are included with the manuscript (e.g. as source data or supplementary information), listing which data are included (e.g. by figure panels and data types) and mentioning any restrictions on availability.

• If a dataset has a Digital Object Identifier (DOI) as its unique identifier, we strongly encourage including this in the Reference list and citing the dataset in the Methods.

We recommend that you upload the step-by-step protocols used in this manuscript to the Protocol Exchange. More details can found at www.nature.com/protocolexchange/about.

DISPLAY ITEMS – main display items are limited to 6-8 main figures and/or main tables. For Supplementary Information see below.

FIGURES – Colour figure publication costs $395 per colour figure. All panels of a multi-panel figure must be logically connected and arranged as they would appear in the final version. Unnecessary figures and figure panels should be avoided (e.g. data presented in small tables could be stated briefly in the text instead).

All imaging data should be accompanied by scale bars, which should be defined in the legend.

Cropped images of gels/blots are acceptable, but need to be accompanied by size markers, and to retain visible background signal within the linear range (i.e. should not be saturated). The boundaries of panels with low background have to be demarked with black lines. Splicing of panels should only be considered if unavoidable, and must be clearly marked on the figure, and noted in the legend with a statement on whether the samples were obtained and processed simultaneously. Quantitative comparisons between samples on different gels/blots are discouraged; if this is unavoidable, it has be performed for samples derived from the same experiment with gels/blots were processed in parallel, which needs to be stated in the legend.

Figures should be provided at approximately the size that they are to be printed at (single column is 86 mm, double column is 170 mm) and should not exceed an A4 page (8.5 x 11"). Reduction to the scale that will be used on the page is not necessary, but multi-panel figures should be sized so that the whole figure can be reduced by the same amount at the smallest size at which essential details in each panel are visible. In the interest of our colour-blind readers we ask that you avoid using red and green for contrast in figures. Replacing red with magenta and green with turquoise are two possible colour-safe alternatives. Lines with widths of less than 1 point should be avoided. Sans serif typefaces, such as Helvetica (preferred) or Arial should be used. All text that forms part of a figure should be rewritable and removable.

We accept files from the following graphics packages in either PC or Macintosh format:

- For line art, graphs, charts and schematics we prefer Adobe Illustrator (.AI), Encapsulated PostScript (.EPS) or Portable Document Format (.PDF). Files should be saved or exported as such directly from the application in which they were made, to allow us to restyle them according to our journal house style.

- We accept PowerPoint (.PPT) files if they are fully editable. However, please refrain from adding PowerPoint graphical effects to objects, as this results in them outputting poor quality raster art. Text used for PowerPoint figures should be Helvetica (preferred) or Arial.

- We do not recommend using Adobe Photoshop for designing figures, but we can accept Photoshop generated (.PSD or .TIFF) files only if each element included in the figure (text, labels, pictures, graphs, arrows and scale bars) are on separate layers. All text should be editable in ‘type layers’ and line-art such as graphs and other simple schematics should be preserved and embedded within 'vector smart objects’ - not flattened raster/bitmap graphics.

- Some programs can generate Postscript by 'printing to file' (found in the Print dialogue). If using an application not listed above, save the file in PostScript format or email our Art Editor, Allen Beattie for advice (a.beattie@nature.com).

Regardless of format, all figures must be vector graphic compatible files, not supplied in a flattened raster/bitmap graphics format, but should be fully editable, allowing us to highlight/copy/paste all text and move individual parts of the figures (i.e. arrows, lines, x and y axes, graphs, tick marks, scale bars etc). The only parts of the figure that should be in pixel raster/bitmap format are photographic images or 3D rendered graphics/complex technical illustrations.

All placed images (i.e. a photo incorporated into a figure) should be on a separate layer and independent from any superimposed scale bars or text. Individual photographic images must be a minimum of 300+ DPI (at actual size) or kept constant from the original picture acquisition and not decreased in resolution post image acquisition. All colour artwork should be RGB format.

FIGURE LEGENDS – must not exceed 350 words for each figure to allow fit on a single printed NCB page together with the figure. They must include a brief title for the whole figure, and short descriptions of each panel with definitions of the symbols used, but without detailing methodology.

TABLES – main tables should be provided as individual Word files, together with a brief title and legend. For supplementary tables see below.

SUPPLEMENTARY INFORMATION – Supplementary information is material directly relevant to the conclusion of a paper, but which cannot be included in the printed version in order to keep the manuscript concise and accessible to the general reader. Supplementary information is an integral part of a Nature Cell Biology publication and should be prepared and presented with as much care as the main display item, but it must not include non-essential data or text, which may be removed at the editor's discretion. All supplementary material is fully peer-reviewed and published online as part of the HTML version of the manuscript. Supplementary Figures and Supplementary Notes are appended at the end of the main PDF of the published manuscript.

Supplementary items should relate to a main text figure, wherever possible, and should be mentioned sequentially in the main manuscript, designated as Supplementary Figure, Table, Video, or Note, and numbered continuously (e.g. Supplementary Figure 1, Supplementary Figure 2, Supplementary Table 1, Supplementary Table 2 etc.).

Unprocessed scans of all key data generated through electrophoretic separation techniques need to be presented in a supplementary figure that should be labeled and numbered as the final supplementary figure, and should be mentioned in every relevant figure legend. This figure does not count towards the total number of figures and is the only figure that can be displayed over multiple pages, but should be

provided as a single file, in PDF or TIFF format. Data in this figure can be displayed in a relatively informal style, but size markers and the figures panels corresponding to the presented data must be indicated.

The total number of Supplementary Figures (not including the "unprocessed scans" Supplementary Figure) should not exceed the number of main display items (figures and/or tables (see our Guide to Authors and March 2012 editorial http://www.nature.com/ncb/authors/submit/index.html#suppinfo; http://www.nature.com/ncb/journal/v14/n3/index.html#ed). No restrictions apply to Supplementary Tables or Videos, but we advise authors to be selective in including supplemental data.

Each Supplementary Figure should be provided as a single page and as an individual file in one of our accepted figure formats and should be presented according to our figure guidelines (see above). Supplementary Tables should be provided as individual Excel files. Supplementary Videos should be provided as .avi or .mov files up to 50 MB in size. Supplementary Figures, Tables and Videos much be accompanied by a separate Word document including titles and legends.

GUIDELINES FOR EXPERIMENTAL AND STATISTICAL REPORTING

REPORTING REQUIREMENTS – To improve the quality of methods and statistics reporting in our papers we have recently revised the reporting checklist we introduced in 2013. We are now asking all life sciences authors to complete two items: an Editorial Policy Checklist (found here https://www.nature.com/authors/policies/Policy.pdf) that verifies compliance with all required editorial policies and a Reporting Summary (found here https://www.nature.com/authors/policies/ReportingSummary.pdf) that collects information on experimental design and reagents. These documents are available to referees to aid the evaluation of the manuscript. Please note that these forms are dynamic 'smart pdfs' and must therefore be downloaded and completed in Adobe Reader. We will then flatten them for ease of use by the reviewers. If you would like to reference the guidance text as you complete the template, please access these flattened versions at http://www.nature.com/authors/policies/availability.html.

STATISTICS – Wherever statistics have been derived the legend needs to provide the n number (i.e. the sample size used to derive statistics) as a precise value (not a range), and define what this value represents. Error bars need to be defined in the legends (e.g. SD, SEM) together with a measure of centre (e.g. mean, median). Box plots need to be defined in terms of minima, maxima, centre, and percentiles. Ranges are more appropriate than standard errors for small data sets. Wherever statistical significance has been derived, precise p values need to be provided and the statistical test used needs to be stated in the legend. Statistics such as error bars must not be derived from n<3. For sample sizes of n<5 please plot the individual data points rather than providing bar graphs. Deriving statistics from technical replicate samples, rather than biological replicates is strongly discouraged. Wherever statistical

significance has been derived, precise p values need to be provided and the statistical test stated in the legend.

Information on how many times each experiment was repeated independently with similar results needs to be provided in the legends and/or Methods for all experiments, and in particular wherever representative experiments are shown.

We strongly recommend the presentation of source data for graphical and statistical analyses as a separate Supplementary Table, and request that source data for all independent repeats are provided when representative experiments of multiple independent repeats, or averages of two independent experiments are presented. This supplementary table should be in Excel format, with data for different figures provided as different sheets within a single Excel file. It should be labelled and numbered as one of the supplementary tables, titled "Statistics Source Data", and mentioned in all relevant figure legends.

--------- Please don't hesitate to contact NCB@nature.com should you have queries about any of the above requirements ---------

Author Rebuttal, second revision:

Point-by-point response to reviewers' comments

Reviewer #1:

Remarks to the Author:
I acknowledge that the authors had given it their best effort to address the points I had raised. So, while there were pieces of data I would've liked to see (and the analysis of Ribo-seq data not powered enough), I understand that sufficient material may not be at hand.
Therefore, I recommend publication of this study.

Having said that, I had asked the authors to provide mRNA measurements to rule out transcriptional changes as the source of observed modulations in protein levels. While they have done this, the error bars on their measurements, at times, exceeds what is customary (for example Fig 3h). When error bars are this large, it is not unexpected that the differenceswill not be statistically significant. Time/resources permitting, the authors should consider tightening these error bars.

Minor:
Fig S4G, the points are missing and converted to crossed-out boxes. This happens when ggplot is imported to AI. Setting useDingbats to false in ggsave will solve this (or changing the font in AI)

We are thankful to the reviewer for his/her comments and supporting the publication of our study. Following the reviewer's suggestion, we performed three additional replicates and tighten the error bars for the mRNA quantification shown in Figure 3h. Importantly, the analysis shows no statistically significant differences in mRNA levels across conditions, which is consistent with the results in our study. Furthermore, we addressed the reviewer's minor point regarding Fig.S4G and replaced the graph as suggested.

Extra comments:
In some ways, my comment in the first round stating that there were two separate stories here, a translation story and a cancer story, was a similar criticism. But I'm not sure what reviewer 2 means by global; at least in case of Ribo-seq, the authors describe a regulon of afew thousand genes that are affected by this pathway. That number, while large, is not global (or if there is a global effect, it is more pronounced for these targets). What may be missing here (and liklely what reviewer 2 is asking), is the downstream effector genes that are responsible for the cancer phenotype. It goes without saying that the vast majority of changes observed are likely neutral and there are only a few genes whose increased expression is driving tumorigenesis. While this paper doesn't have these, and would've beena more complete story with them known, I think the identified master regulators are sufficiently well-characterized to warrant publication. In sum, I agree with reviewer 2 in general that the specific downstream effects that are relevant to tumorigenesis are not described, but I think that this may be a high bar as the paper already contains a ton of new information and insights.

We thank the reviewer for providing additional comments and for acknowledging that "*the identified master regulators are sufficiently well-characterized to warrant publication*".

Reviewer #2:

Remarks to the Author:
The revised MS by Guzzi et al leaves many questions unanswered. I find data in Fig. 1 about binding to PABPC1 clear and convincing. It remains unclear what else the mTOG RNA would bind. (PES is not introduced in the MS and I am not sure what it stands for, unless I missed it).
The profiling data are good, but again they show a global effect on translation, not -as thetitle/abstract suggest- a malignancy specific effect. I raised the question before and

the answer appears to be that all cap-dependent translation promotes cancer and is thereforecancer specific. I am not convinced by the argument or the reference to the RPL23 study.

The link to MDS is based on retrospective associations, this is nice but hardly "compelling" evidence of a role in the disease. I find the description of in vitro studies on MDS stem cells and the rescue studies insufficient to understand. for the in vivo studies I would like to see evidence of mTOG expression and the sufficiency and requirements of the proposed molecular effects.

Limited data have been added to address prior concerns, and I am sorry to say, I find the MS cursory and not ready to publish in its revised form.

As recommended by the editors, we included a statement in the discussion on line 274 “Future work will be required to decipher the oncogenic translational programs, **including specific downstream effector genes** governed by PUS7 and mTOG and the impact on MDS etiology”. Moreover, we tempered our claims on “malignant protein synthesis” changingthe title “…tRNA fragments repress **aberrant** protein synthesis. ....” and the abstract on lines 28-29 “…mTOG, selectively inhibit **aberrant** protein synthesis…”. Lastly, we clarified in lines139-140 that RPL29, RPL23, and EIF6 among the transcripts identified by ribosome profilingwere “…**further examined as prototype 5’PES mRNAs potentially regulated by mTOG-λϑ’…**“, and not because they were the specific drivers of MDS transformation.

Furthermore, we improved the description of the *in vitro* studies on MDS and revised the texton line 210 to “…mTOG-λϑ’ selectively **modulated** protein synthesis in MDS-HSPC…”. On line 222, we specified that the rescue of 5’PES mRNA translation in MDS-L cells included **RPL29 and EIF6** and at the end of page 6 on line 226 that “…epistatic interaction between mTOG-λϑ’ and PAIP1 critical **for balancing translation** in MDS cells”. Finally, we added the sentence “…**Of note, RPL23, RPL29 and EIF6 protein levels were selectively modulatedonly in the colonies generated from HR-MDS cells transduced with mTOG-λϑ’ and siPAIP1 (Extended data Fig. 8b)**” on line 238 that better defines the observed rescue effects.

Reviewer #3:

Remarks to the Author:
The authors have done a solid job addressing my original concerns. In particular, they have sufficiently clarified their findings using the MDS primary samples (Figure 4). I have no additional comments.

We are pleased that the reviewer is satisfied with our work and are grateful for his/her helpful comments and suggestions, which helped us strengthen the manuscript.

natureresearch

Decision Letter, third revision:

Subject: Your manuscript, NCB-B44478C
Message:

Our ref: NCB-B44478C

23rd November 2021

Dear Dr. Bellodi,

Thank you for submitting your revised manuscript "Pseudouridine-modified tRNA fragments repress aberrant protein synthesis and predict leukemic progression in myelodysplastic syndrome" (NCB-B44478C). It has now been seen by our editors. We find that the paper has improved in revision, and therefore we'll be happy in principle to publish it in Nature Cell Biology, pending minor revisions to comply with our editorial and formatting guidelines.

The current version of your manuscript is in a PDF format. Please email us a copy of the file in an editable format (Microsoft Word or LaTex)-- we can not proceed with PDFs at this stage.

We are now performing detailed checks on your paper and will send you a checklist detailing our editorial and formatting requirements in about a week. Please do not upload the final materials and make any revisions until you receive this additional information from us.

Thank you again for your interest in Nature Cell Biology Please do not hesitate to contact me if you have any questions.

Sincerely,

Jie Wang, PhD
Senior Editor
Nature Cell Biology

Tel: +44 (0) 207 843 4924
email: jie.wang@nature.com

Decision letter, final requests:

Date: 8th December 21 00:37:36
Last Sent: 8th December 21 00:37:36
Triggered By: Ziqian Li
From: ziqian.li@nature.com
To: Cristian.Bellodi@med.lu.se
CC: ncb@springernature.com, jie.wang@nature.com
BCC: ziqian.li@nature.com
Subject: NCB: Your manuscript, NCB-B44478C
Message: Our ref: NCB-B44478C

8th December 2021

Dear Dr. Bellodi,

Thank you for your patience as we've prepared the guidelines for final submission of your Nature Cell Biology manuscript, "Pseudouridine-modified tRNA fragments repress aberrant protein synthesis and predict leukemic progression in myelodysplastic syndrome" (NCB-B44478C). Please carefully follow the step-by-step instructions provided in the attached file, and add a response in each row of the table to indicate the changes that you have made. Ensuring that each point is addressed will help to ensure that your revised manuscript can be swiftly handed over to our production team.

We would like to start working on your revised paper, with all of the requested files and forms, as soon as possible (preferably within one week). Please get in contact with us if you anticipate delays.

When you upload your final materials, please include a point-by-point response to any remaining reviewer comments.

If you have not done so already, please alert us to any related manuscripts from your group that are under consideration or in press at other journals, or are being written up for submission to other journals (see: https://www.nature.com/nature-research/editorial-policies/plagiarism#policy-on-duplicate-publication for details).

In recognition of the time and expertise our reviewers provide to Nature Cell Biology's editorial process, we would like to formally acknowledge their contribution to the external peer review of your manuscript entitled "Pseudouridine-modified tRNA fragments repress aberrant protein synthesis and predict leukemic progression in myelodysplastic syndrome". For those reviewers who give their assent, we will be publishing their names alongside the published article.

Nature Cell Biology offers a Transparent Peer Review option for new original research manuscripts submitted after December 1st, 2019. As part of this initiative, we encourage our authors to support increased transparency into the peer review process by agreeing to have the reviewer comments, author rebuttal letters, and editorial decision letters published as a Supplementary item. When you submit your final files please clearly state in your cover letter whether or not you would like to participate in this initiative. Please note that failure to state your preference will result in delays in accepting your manuscript for publication.

Cover suggestions

As you prepare your final files we encourage you to consider whether you have any images or illustrations that may be appropriate for use on the cover of Nature Cell Biology.

Covers should be both aesthetically appealing and scientifically relevant, and should be supplied at the best quality available. Due to the prominence of these images, we do not generally select images featuring faces, children, text, graphs, schematic drawings, or collages on our covers.

We accept TIFF, JPEG, PNG or PSD file formats (a layered PSD file would be ideal), and the image should be at least 300ppi resolution (preferably 600-1200 ppi), in CMYK colour mode.

If your image is selected, we may also use it on the journal website as a banner image, and may need to make artistic alterations to fit our journal style.

Please submit your suggestions, clearly labeled, along with your final files. We'll be in touch if more information is needed.

Nature Cell Biology has now transitioned to a unified Rights Collection system which will allow our Author Services team to quickly and easily collect the rights and permissions required to publish your work. Approximately 10 days after your paper is formally accepted, you will receive an email in providing you with a link to complete the grant of rights. If your paper is eligible for Open Access, our Author Services team will also be in touch regarding any additional information that may be required to arrange payment for your article.

Please note that Nature Cell Biology is a Transformative Journal (TJ). Authors may publish their research with us through the traditional subscription access route or make their paper immediately open access through payment of an article-processing charge (APC). Authors will not be required to make a final decision about access to their article until it has been accepted. Find out more about Transformative Journals

Authors may need to take specific actions to achieve compliance with funder and institutional open access mandates. For submissions from January 2021, if your research is supported by a funder that requires immediate open access (e.g. according to Plan S principles) then you should select the gold OA route, and we will direct you to the compliant route where possible. For authors selecting the subscription publication route our standard licensing terms will need to be accepted, including our self-archiving policies. Those standard licensing terms will supersede any other terms that the author or any third party may assert apply to any version of the manuscript.

Please note that you will not receive your proofs until the publishing agreement has been received through our system.

For information regarding our different publishing models please see our Transformative Journals page. If you have any questions about costs, Open Access requirements, or our legal forms, please contact ASJournals@springernature.com.

Please use the following link for uploading these materials:
[REDACTED]

If you have any further questions, please feel free to contact us. Many thanks!

Best regards,

Ziqian Li
Editorial Assistant
Nature Cell Biology

On behalf of

Jie Wang, PhD
Senior Editor
Nature Cell Biology

Tel: +44 (0) 207 843 4924
email: jie.wang@nature.com

natureresearch

Final Decision Letter:

Subject: Decision on Nature Cell Biology submission NCB-B44478D

Message:

Dear Dr Bellodi,

I am pleased to inform you that your manuscript, "Pseudouridine-modified tRNA fragments repress aberrant protein synthesis and predict leukemic progression in myelodysplastic syndrome", has now been accepted for publication in Nature Cell Biology.

Thank you for sending us the final manuscript files to be processed for print and online production, and for returning the manuscript checklists and other forms. Your manuscript will now be passed to our production team who will be in contact with you if there are any questions with the production quality of supplied figures and text.

Over the next few weeks, your paper will be copyedited to ensure that it conforms to Nature Cell Biology style. Once your paper is typeset, you will receive an email with a link to choose the appropriate publishing options for your paper and our Author Services team will be in touch regarding any additional information that may be required.

After the grant of rights is completed, you will receive a link to your electronic proof via email with a request to make any corrections within 48 hours. If, when you receive your proof, you cannot meet this deadline, please inform us at rjsproduction@springernature.com immediately.

You will not receive your proofs until the publishing agreement has been received through our system.

Due to the importance of these deadlines, we ask that you please let us know now whether you will be difficult to contact over the next month. If this is the case, we ask you provide us with the contact information (email, phone and fax) of someone who will be able to check the proofs on your behalf, and who will be available to address any last-minute problems.

If you have any questions about our publishing options, costs, Open Access requirements, or our legal forms, please contact ASJournals@springernature.com

Once your paper has been scheduled for online publication, the Nature press office will be in touch to confirm the details. An online order form for reprints of your paper is available at https://www.nature.com/reprints/author-reprints.html. All co-authors, authors' institutions and authors' funding agencies can order reprints using the form appropriate to their geographical region.

Publication is conditional on the manuscript not being published elsewhere and on there being no announcement of this work to any media outlet until the online publication date in Nature Cell Biology.

Please note that Nature Cell Biology is a Transformative Journal (TJ). Authors may publish their research with us through the traditional subscription access route or make their paper immediately open access through payment of an article-processing charge (APC). Authors will not be required to make a final decision about access to their article until it has been accepted. Find out more about Transformative Journals

Authors may need to take specific actions to achieve compliance with funder and institutional open access mandates. For submissions from January 2021, if your research is supported by a funder that requires immediate open access (e.g. according to Plan S principles) then you should select the gold OA route, and we will direct you to the compliant route where possible. For authors selecting the subscription publication route our standard licensing terms will need to be accepted, including our self-archiving policies. Those standard licensing terms will supersede any other terms that the author or any third party may assert apply to any version of the manuscript.

To assist our authors in disseminating their research to the broader community, our SharedIt initiative provides you with a unique shareable link that will allow anyone (with or without a subscription) to read the published article. Recipients of the link with a subscription will also be able to download and print the PDF.

If your paper includes color figures, please be aware that in order to help cover some of the additional cost of four-color reproduction, Nature Research charges our authors a fee for the printing of their color figures. Please contact our offices for exact pricing and details.

As soon as your article is published, you will receive an automated email with your shareable link.

If you have not already done so, we strongly recommend that you upload the step-by-step protocols used in this manuscript to the Protocol Exchange (www.nature.com/protocolexchange), an open online resource established by Nature Protocols that allows researchers to share their detailed experimental know-how. All uploaded protocols are made freely available, assigned DOIs for ease of citation and are fully searchable through nature.com. Protocols and the Nature and Nature research journal papers in which they are used can be linked to one another, and this link is clearly and prominently visible in the online versions of both papers. Authors who performed the specific experiments can act as primary authors for the Protocol as they will be best placed to share the methodology details, but the Corresponding Author of the present research paper should be included as one of the authors. By uploading your Protocols to Protocol Exchange, you are enabling researchers to more readily reproduce or adapt the methodology you use, as well as increasing the visibility of your protocols and papers. You

can also establish a dedicated page to collect your lab Protocols. Further information can be found at www.nature.com/protocolexchange/about

You can use a single sign-on for all your accounts, view the status of all your manuscript submissions and reviews, access usage statistics for your published articles and download a record of your refereeing activity for the Nature journals.

Please feel free to contact us if you have any questions.

With kind regards,

Jie Wang, PhD
Senior Editor
Nature Cell Biology

Tel: +44 (0) 207 843 4924
email: jie.wang@nature.com

Click here if you would like to recommend Nature Cell Biology to your librarian
http://www.nature.com/subscriptions/recommend.html#forms

** Visit the Springer Nature Editorial and Publishing website at www.springernature.com/editorial-and-publishing-jobs for more information about our career opportunities. If you have any questions please click here.**
